# Dissecting genomic regions and underlying sheath blight resistance traits in rice ( *Oryza sativa* L.) using a genome‐wide association study

**DOI:** 10.1002/pld3.540

**Published:** 2023-11-23

**Authors:** R Naveenkumar, Annamalai Anandan, Seenichamy Rathinam Prabhukarthikeyan, Anumalla Mahender, Ganesan Sangeetha, Shyam Saran Vaish, Pawan Kumar Singh, Waseem Hussain, Jauhar Ali

**Affiliations:** ^1^ Crop Improvement Division ICAR‐National Rice Research Institute (NRRI) Cuttack Odisha India; ^2^ Institute of Agricultural Sciences Banaras Hindu University Varanasi Uttar Pradesh India; ^3^ Division of Plant Pathology, School of Agricultural Sciences Karunya Institute of Technology and Sciences Coimbatore Tamil Nadu India; ^4^ ICAR‐Indian Institute of Seed Science Bangalore Karnataka India; ^5^ Crop Protection Division ICAR‐National Rice Research Institute Cuttack Odisha India; ^6^ Rice Breeding Innovation Platform International Rice Research Institute (IRRI) Los Baños Laguna Philippines; ^7^ Division of Crop Protection ICAR‐Indian Institute of Horticultural Research Bangalore Karnataka India

**Keywords:** association mapping, disease score, molecular genetic diversity, quantitative trait loci, rice

## Abstract

The productivity of rice is greatly affected by the infection of the plant pathogenic fungus *Rhizoctonia solani*, which causes a significant grain yield reduction globally. There exist a limited number of rice accessions that are available to develop sheath blight resistance (ShB). Our objective was to identify a good source of the ShB resistance, understand the heritability, and trait interactions, and identify the genomic regions for ShB resistance traits by genome‐wide association studies (GWAS). In the present study, a set of 330 traditional landraces and improved rice varieties were evaluated for ShB resistance and created a core panel of 192 accessions used in the GWAS. This panel provides a more considerable amount of genetic variance and found a significant phenotypic variation among the panel of rice accessions for all the agro‐morphological and disease‐resistance traits over the seasons. The infection rate of ShB and disease reaction were calculated as percent disease index (PDI) and area under the disease progress curve (AUDPC). The correlation analysis showed a significant positive association between PDIs and AUPDC and a negative association between PDI and plant height, flag leaf length, and grain yield. The panel was genotyped with 133 SSR microsatellite markers, resulting in a genome coverage of 314.83 Mb, and the average distance between markers is 2.53 Mb. By employing GLM and MLM (Q + K) models, 30 marker–trait associations (MTAs) were identified with targeted traits over the seasons. Among these QTLs, eight were found to be novel and located on 2, 4, 8, 10, and 12 chromosomes, which explained the phenotypic variation ranging from 5% to 15%. With the GWAS approach, six candidate genes were identified. Os05t0566400, Os08t0155900, and Os09t0567300 were found to be associated with defense mechanisms against ShB. These findings provided insights into the novel donors of IC283139, IC 277248, Sivappuchithirai Kar, and Bowalia. The promising genomic regions on 10 of 12 chromosomes associated with ShB would be useful in developing rice varieties with durable disease resistance.

## 1 INTRODUCTION

Sheath blight disease of rice is caused by basidiomycetous necrotrophic fungi *Rhizoctonia solani* causes significant risk to rice cultivation across the globe. It is the second most prevalent disease next to the rice blast (Molla et al., 2020). The disease is mainly widespread in Asia, Africa, and America, and it causes severe yield losses in rice fields (Li et al., 2021; Savary et al., 1995). *R. solani* is a soil‐borne pathogen that survives in the soil as sclerotia or mycelium in the infected plant debris of the host plant and continues its life cycle in the necrotrophic phase (Neha et al.*,* 2016). The fungal pathogen has a broad range of hosts across 32 families of plants (Gangopadhyay & Chakraborty, 1982) and infects 200 plant species (Dey et al., 2016). The pathogen is genetically more diverse with 14 different anastomosis groups (AG) that had been reported from *R. solani* isolates that is, AG1 to AG13 and AGB1 (Carling et al., 2002). Among the AG1, *R. solani* isolates were further subdivided into three subgroups, namely, IA, IB, and IC, of which *R. solani* AG1 IA isolates were responsible for sheath blight disease of rice (Goswami et al.*,* 2019; Taheri et al., 2007), resulting in 50% yield loss, adversely disturbing the quality of straw by decreasing fodder value (Dey et al., 2016).

The lack of established resistant sources against *R. solani* instigate rice farmers to manage the disease by adapting cultural practices and chemical fungicides (Goswami et al., 2019). Besides, the excessive use of chemical fungicides remains in the soil and increases the chances of an unfriendly environment and several health issues. Moreover, there is a chance of developing resistance to pathogens against the continuous use of fungicides. The development of resistant rice genotypes against sheath blight disease needs to be improved due to the unsuccessful in the identification of stable resistant donors (Yadav et al., 2015). The high genetic variability of *R. solani*, a broad range of host compatibility, and the ability to survive across seasons by forming sclerotia that remain dormant in the soil make it difficult to manage the pathogen (Molla et al., 2020).

Several studies explored the identification of quantitative trait loci (QTLs) resistance to sheath blight disease. To date, around 100 QTLs conferring resistance to sheath blight disease have been mapped in rice using different types of mapping population, namely, F_2_ (Che et al., 2003; Yadav et al., 2015; Zou et al., 2000), F_2:3_ (Bal et al., 2020; Sharma et al., 2009; Wen et al., 2015), F_4_ (Li et al., 1995), recombinant inbred lines (RILs) (Channamallikarjuna et al., 2010; Goad et al., 2020; Han et al., 2003; Liu et al., 2009, 2014; Pinson et al., 2005), near‐isogenic lines (NILs) (Yin et al., 2009), back‐cross breeding populations (Sato et al., 2004; Taguchi‐Shiobara et al., 2013; Yadav et al., 2015; Zuo et al., 2008), chromosomal segment substitution lines (Zhu et al. 2014), and double haploids (DHs) (Kunihiro et al., 2002; Nelson et al., 2012; Xu et al., 2011). Recently, focusing gene tagging efforts from traditional QTL mapping to linkage disequilibrium (LD) and from biparental crosses to selection of a natural population of lines‐based association study became a powerful tool in mapping the genes of interest (Goldstein & Weale, 2001), and this leads to the most effective utilization of *ex situ* conserved natural genetic diversity of crop germplasm resources throughout the world. A few recent studies have reported identifying gene/QTL resistance against sheath blight disease using genome‐wide association (GWA) studies. Jia et al. (2012) identified 10 QTLs responsible for sheath blight resistance by GWA study using 155 genome‐wide SSR markers in 217 sub‐core entries of USDA rice core collection. Similarly, Chen et al. (2019) performed a GWA study in 299 rice genotypes of RDP1 (rice diversity panel 1) with 44,000 high‐density single nucleotide polymorphism (SNP) markers. They identified 11 SNP loci that were significantly associated with sheath blight resistance. Besides, Zhang et al. (2019) experimented on sheath blight resistance using 2,977,750 SNP loci in 563 rice accessions. They detected 132, 562, and 75 SNP loci associated with lesion height, relative lesion height (RLH), and culm length, respectively. However, stable QTLs identified across seasons/locations from multiple mapping populations would be desirable to use in the crop improvement program. To date, only two QTLs (*qSBR11‐1* and *qSb‐11* ^*LE*^) resistance to sheath blight were fine mapped (Channamallikarjuna et al., 2010; Zuo et al., 2013). Earlier sheath blight resistance mapping studies typically relied on lesion length to differentiate the population.

In the present investigation, we have collected 330 diverse sets of rice genotypes, including both traditional landraces and improved rice varieties. They were evaluated against ShB disease at Banaras Hindu University (BHU), Varanasi, India. To explore further, a panel consisting of 192 selected genotypes was formed to represent the initial population. The specific goal of this study was to (i) examine the phenotypic, genotypic variances and relationship between agro‐morphological and ShB disease‐associated parameters over the season, (ii) understand the molecular diversity pattern, population structure, and relationship between the assumed subpopulation of the genotypes of core panel based on the SSRs, and (iii) identify the genomic regions that are associated with ShB tolerance traits and further to reveals the potential candidate genes and their mechanism.

## 2 RESULTS

### 2.1 Experiment 1

**FIGURE 1:**
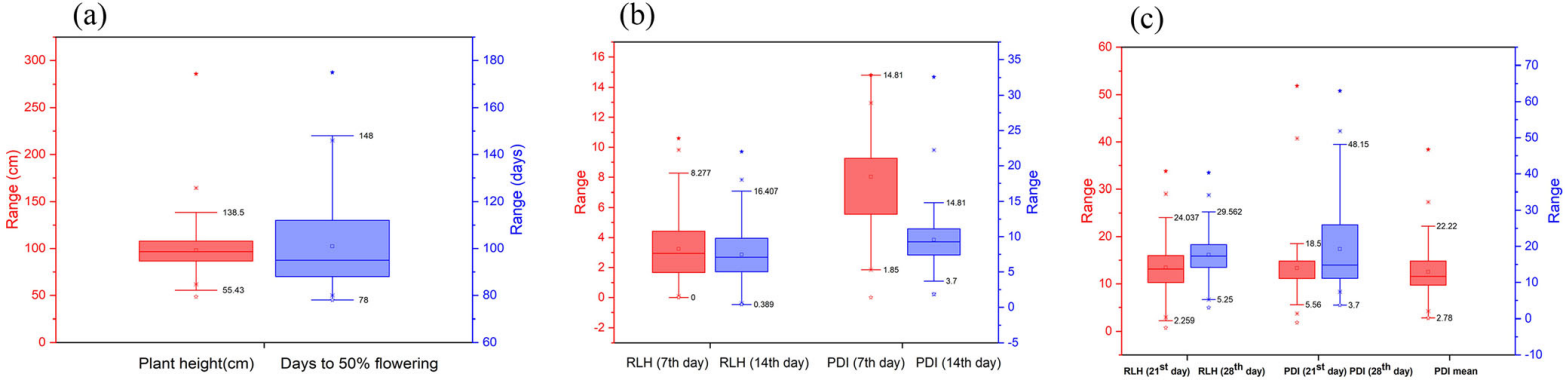
Box plot showing the population distribution for sheath blight‐related traits of 330 rice genotypes. (a) Plant height and days to 50% flowering. (b) RLH of 7th and 14th day, PDI of 7th and 14th day. (c) RLH of 21st and 28th day, PDI of 21st day, 28th day, and PDI mean. With the progression of days from 7 to 28 days, RLH, and PDI increase. The upper, median, and lower quartiles of boxes represent the 75th, 50th, and 25th percentiles of the population, respectively. The square box inside the quartile box represents mean, and asterix represents outliers.

In the present investigation, the summary of descriptive statistics and distribution pattern of data observed related to sheath blight traits of 330 genotypes are presented in Table 1. Among the different sheath blight‐related traits studied, 7th, 14th, 21st, and 28th day RLH and 7th and 14th day percent disease index (PDI) were negatively skewed (<−1), whereas other traits were positively skewed. Similarly, kurtosis values were positive for most of the traits, such as plant height and panicle length, 14th and 21st day PDI, mean PDI and 14th day PDI were >3, while 7th day PDI was found to be negative. It clearly showed the distribution of the population was leptokurtic, which indicates that germplasm was not more closely bunched around the mean. The coefficient of variation (CV) was considerable, and it was higher for RLH 7th day (64%) followed by 28th (52%) and 21st (49%) day PDI, while the least CV was found in 28th day RLH (16%). Higher CV indicated that the population in this study exhibited a higher variability. The mean, median, and mode were similar in all traits other than plant height, 7th day, and 14th day RLH, which indicates distribution frequency was normal for these traits (Figure 1).

**TABLE 1:** Descriptive statistics for preliminary screening of 330 rice germplasm on wet season 2018.

	Mean	SD	SE of mean	Skewness	Kurtosis	CV	Mode	Mini	1st quartile (Q1)	Median	3rd quartile (Q3)	Max
Plant height	98.10	21.98	1.21	3.79	29.28	0.22	83.10	48.43	86.55	96.68	107.93	286.00
No. of tillers/plant	8.22	2.90	0.16	0.57	0.37	0.35	6.17	2.00	6.17	7.92	10.00	18.00
Panicle length	20.78	4.19	0.23	3.45	24.12	0.20	17.90	11.82	18.48	20.63	22.58	56.77
Days to 50% f	100.87	17.14	0.94	1.10	0.66	0.17	94.00	78.00	88.00	95.00	112.00	175.00
RLH 7th day	3.24	2.06	0.11	0.94	0.96	0.64	0.00	0.00	1.66	2.95	4.42	10.59
RLH 14th day	7.45	3.57	0.20	0.60	0.86	0.48	1.64	0.39	5.04	7.09	9.78	21.99
RLH 21st day	13.40	4.88	0.27	0.74	2.05	0.36	10.71	0.74	10.28	13.12	15.97	33.78
RLH 28th day	17.63	5.61	0.31	0.54	1.27	0.32	15.73	3.01	14.14	17.31	20.45	40.28
PDI 7th day	8.03	2.75	0.15	−0.64	−0.08	0.34	9.26	0.00	5.56	9.26	9.26	14.81
PDI 14th day	9.55	3.24	0.18	1.74	11.41	0.34	11.11	1.85	7.41	9.26	11.11	32.58
PDI 21st day	13.30	6.58	0.36	2.70	9.86	0.49	11.11	1.85	11.11	11.11	14.81	51.85
PDI 28th day	19.22	10.07	0.55	1.39	2.21	0.52	11.11	3.70	11.11	14.81	25.93	62.96
PDI (mean)	12.52	4.74	0.26	1.66	5.05	0.38	11.11	2.78	9.72	11.57	14.81	38.37

**FIGURE 2:**
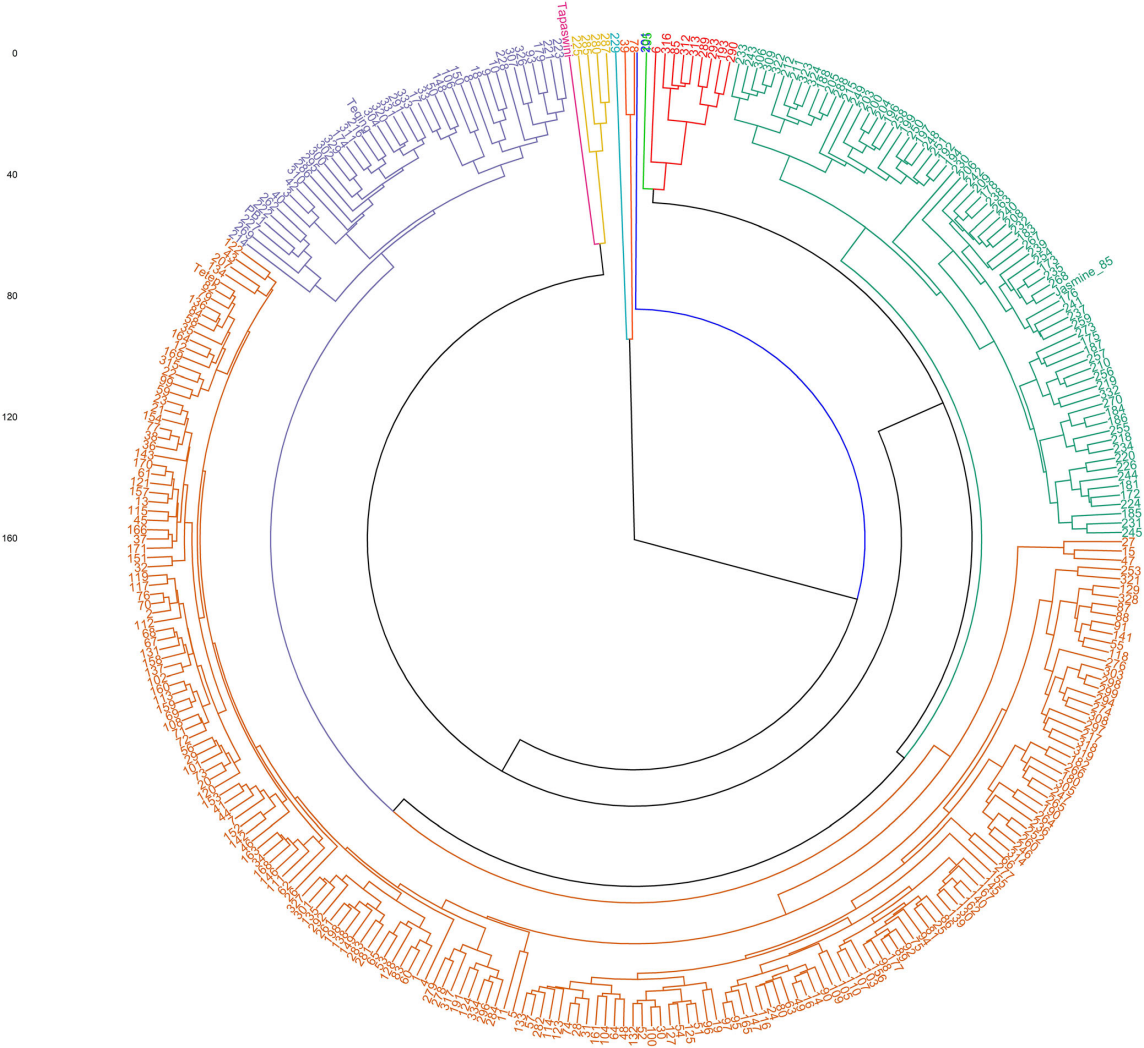
Hierarchical clustering of 330 rice genotypes based on morphological variation among the population screened against sheath blight disease. Three hundred and thirty genotypes grouped into three major clusters. Cluster 1 had five genotypes, cluster 2 had four genotypes, and cluster 3 subdivided into two sub‐clusters 3a and 3b. Sub‐cluster 3a (green and red color) contains 10 genotypes representing MR and R genotypes, and sub‐cluster 3b contains 71 genotypes subdivided again into 3b‐1 (green) and 3b‐2 (subdivided into two sub‐clusters 3b‐2A (brown; 199 genotypes represents MR and R) and 3b‐2B (purple; 41 genotypes represents MR and MS)).

The hierarchical cluster analysis was performed across 330 genotypes to find their similarity based on the disease reaction against sheath blight (Figure 2). They were classified into three major clusters. Cluster 1 had five genotypes, composed of moderately susceptible (MS) and susceptible genotypes (Tapaswini, 225, 285, 280, and 287), whereas cluster 2 contains four genotypes (229, 39, 78, and 201). On the other hand, cluster 3 grouped the rest of the genotypes with two sub‐clusters, namely, 3a and 3b. Cluster 3a (green and red color) contains 10 genotypes belongs to moderately resistant (MR) and resistant (R) reaction to the pathogen, whereas cluster 3b was further subdivided into two clusters such as 3b‐1 (green) and 3b‐2. Cluster 3b‐1 (green) had 71 genotypes, while 3b‐2 were further subdivided into two sub‐clusters, 3b‐2A and 3b‐2B. Cluster 3b‐2A (brown) consisted of 199 genotypes showing MR and R reaction to *R. solani*, and cluster 3b‐2B (purple) contains 41 genotypes showed MR and MS reaction to sheath blight disease (Figure 2).

### 2.2 Experiment 2

Population distribution of the core panel suggests that the population captured a wide range of variation over the seasons (dry and wet season 2019) (Table S3). The mean and median were similar over the seasons, which suggested that population frequency was normal. Among the traits studied in dry season 2019, plant height was negatively skewed (<−1), while other traits were positively skewed (Table S4). In wet season 2019, all the traits were positively skewed except flag leaf width, and ligule shape exhibited negative skewness (Table S5). Similarly, kurtosis values were ranged from −1 to +1 for all the traits over the seasons, other than days to 50% flowering (2.30) and 7th day PDI (6.37) in dry season 2019. In wet season 2019, kurtosis values of culm thickness (31.49), ligule color (15.54), auricle color (23.09), basal leaf sheath color (10.50), and 7th day PDI (8.66) suggested that the distribution frequency of the core panel population was platykurtic. The CV was considerable, and it differed from 11.60% (panicle length) to 40.87% (grain yield per plant) for dry season 2019. In wet season 2019, it ranged from 12.33% (flag leaf width) to 72.53% (apiculus color). It indicated that the panel population of this study showed higher variability.

Analysis of variance (ANOVA) revealed significant (*p* > .01) variation among genotypes (G), seasons (S), and G × S for all the traits observed other than flag leaf width and days to 50% flowering of treatments, culm thickness of season, and grain yield of G × S (Table 2). Heritability (h^2^) and genetic advance (GA) estimates give an insight into the extent of genetic control of the specific trait and phenotypic reliability in projecting its breeding value. Estimated h^2^ broad‐sense ranged from 0.50% (grain yield) to 42.42% for panicle length in season 1 and 3.63% (flag leaf width) to 42.73% (internodal length) in season 2. The GA was calculated as a percentage of mean and ranged from 0.06 (grain yield) to 44.94 (area under the disease progress curve [AUDPC]) in season 1, wherein season 2 it was ranged from 0.27 (PDI of 7th day) to 53.93 (AUDPC) (Table S6).

**TABLE 2:** Analysis of variance (ANOVA) for agronomic and sheath blight related traits in rice genotypes over the season.

Trait	Genotypes (G)	Season (S)	G × S	Error
Days to 50% flowering	741.95ns (97.7%)	206.26** (0.1%)	9.95** (1.3%)	3.25
Plant height	1073.52** (47.4%)	2679.89** (0.6%)	723.21** (32.0%)	224.81
Panicle length	34.55** (47.6%)	193.60** (1.4%)	22.66** (31.2%)	7.14
Tiller number	26.77** (41.4%)	95.89* (0.8%)	14.54* (22.5%)	11.33
Grain yield	99.21** (52.7%)	132.73* (0.4%)	19.85ns (10.5%)	34.15
Internodal length	30.92** (69.5%)	216.94** (2.6%)	—	12.4
Fag leaf length	39.47** (68.5%)	152.45** (1.4%)	—	17.35
Flag leaf width	0.05ns (50.1%)	0.67** (3.4%)	—	0.05
Culm thickness	1.19** (61.9%)	0.47ns (0.1%)	—	0.73
7th day PDI	13.93** (29.2%)	65.55** (0.7%)	14.19** (29.7%)	9.61
14th day PDI	115.86** (36.6%)	1619.88** 9 (2.7%)	85.40** (26.9%)	53.3
21st day PDI	175.24** (38.8%)	3027.88** (3.5%)	124.60** (27.6%)	67.76
28th day PDI	374.27** (72.1%)	783.78* (0.8%)	—	135.45
Mean PDI	86.88** (39.1%)	892.89** (2.1%)	66.00** (29.7%)	32.03
AUDPC	72856.70** (28.1%)	14507545.25** (29.2%)	61918.95** (23.8%)	24346.6

*Note*: Values in parenthesis explained percentage of variations.

Abbreviation: ns, nonsignificance.

* Significance value at 0.05%.

** Significance value at 0.01%.

**FIGURE 3:**
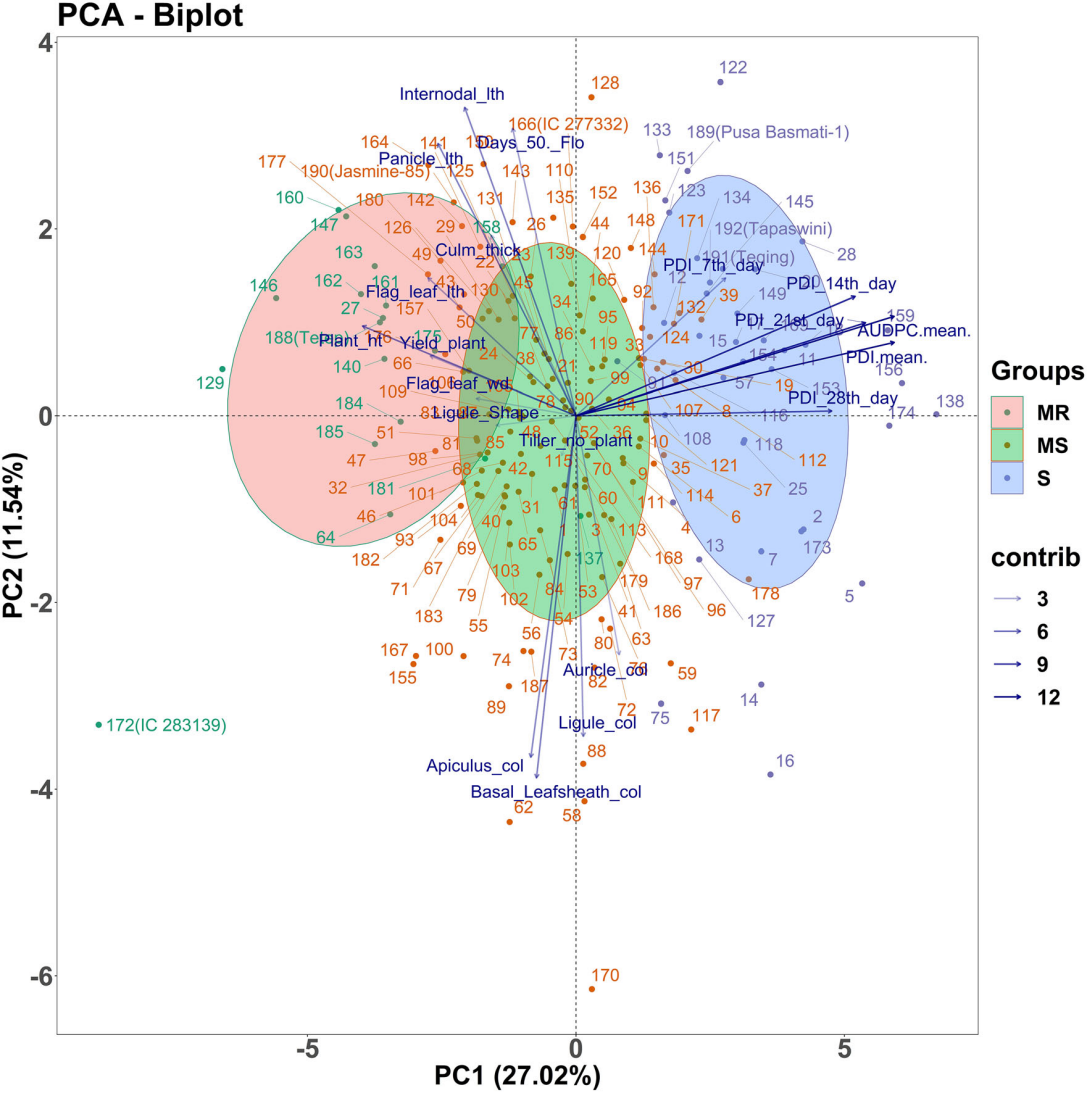
PCA biplot graph represents genotypes in two main principal components for traits associated with sheath blight disease over the season. The two components explained 27.02% and 11.54% of the variance, respectively. The direction and length of the vector indicate the traits' contribution to the first two components in the PCA. The transparency of the trait vectors represents the contribution to the variance in the dataset, ranging from 3% (lightest) to 12% (darkest).

The principal component analysis was performed to categorize genotypes based on their disease reaction and to find the association between the traits responsible for sheath blight resistance. The biplot analysis of season 1 (dry season) registered 41.33% of the variation by PC1 and 14.82% by PC2 (Figure S1). Among the different traits studied, AUDPC, and PDI of different time intervals contributed to a high level of variation compared to other traits, while morphological traits were plotted in opposite quadrant. On the other hand, a similar trend of variation was observed among the traits studied during season 2 (wet season) with a cumulative variability of 38.87% by PC1 and PC2 (Figure S2). The cultivar‐by‐trait biplot of season 1 categorizes 192 genotypes into three groups such as MR, MS, and S. whereas the biplot of season 2 categorizes them into HS, MR, MS, and S. However, the biplot analysis of pooled data depicted a similar trend of association between sheath blight disease‐related traits with 38.56% of variation toward the total variability (Figure 3).

**FIGURE 4:**
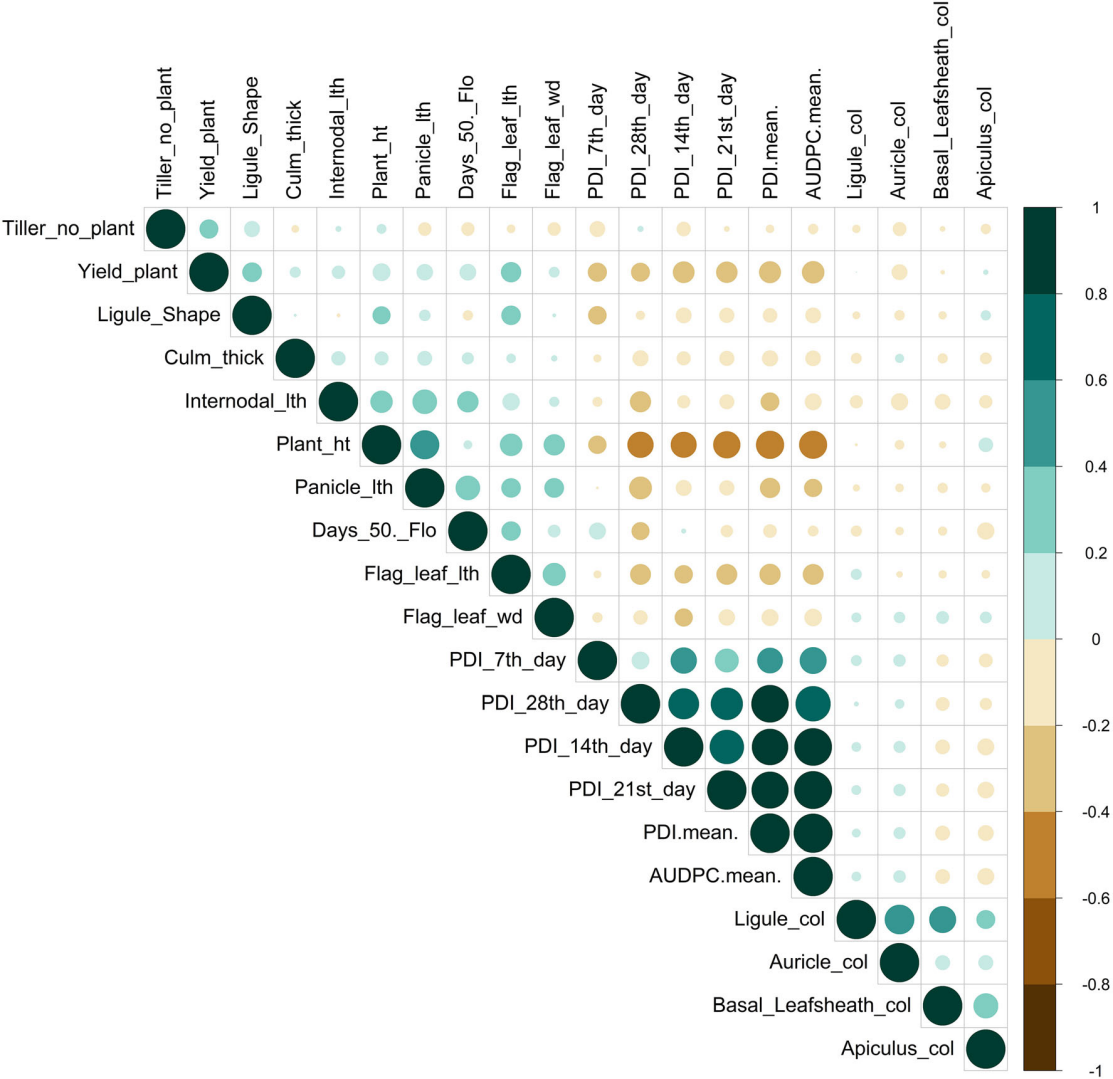
Pearson correlation matrix among the traits associated with sheath blight disease over the season. The color denotes the sort of correlation, where 1 represents complete positive correlation (dark green) and −1 represents complete negative correlation (dark brown) between two traits. Large circle denotes strong, and small circle denotes weaker correlation.

The Pearson correlation analysis was performed to find out the estimated correlation coefficient among the traits associated with traits responsible for sheath blight disease of rice for two seasons. During dry season 2019, a strong positive correlation was found between 21st day PDI and 14th day PDI (0.746), mean PDI (0.919), and AUPDC (0.841). However, a negative association was observed between plant height and disease‐related traits such as PDI and AUPDC (Figure S3). In the 2019 wet season, a similar trend of the strong positive association was observed among PDI of 14th, 21st, 28th, mean, and AUPDC (Figure S4). The estimated correlation coefficient of traits over the season concerned with sheath blight resistance reflected a similar trend of positive association between PDI at all levels of weekly interval and AUPDC. A negative association between PDI and plant height, flag leaf length, and grain yield was also observed (Figure 4).

### 2.3 Genetic diversity

In the present investigation, a shortlisted panel population of 192 rice genotypes of different provinces of India was studied to realize their genetic diversity using 133 sheath blight linked and general SSRs to decipher their genetic relatedness. The output of genetic kinship and diversity parameters are depicted in Table S7. The total number of alleles observed were 366 with 2.781 average alleles per locus. The allele number were differed from one (RM8217, RM188, RM435, RM5304) to seven (RM426) per loci. The major allele frequency values were ranged from 0.239 (RM426) to 1.000 (RM8217, RM188, RM435, RM5304) with an average of 0.642 per marker. Further, the polymorphic information content (PIC) value varied from 0.000 (RM8217, RM188, RM435, RM5304) to 0.794 (RM426) with the mean value of 0.383 per loci. The highest gene diversity observed was 0.819 for the primer RM426, while the least value of 0.000 was observed for three primers (RM8217, RM188, RM435, and RM5304). Heterozygosity values of 133 markers were found to be low and ranged from 0.0000 to 0.142 with an average of 0.013. The mean inbreeding coefficient value of 133 loci was 0.972 and ranged from 0.126 to 1.00 (Table S7).

### 2.4 Genetic relatedness by cluster analysis

**FIGURE 5:**
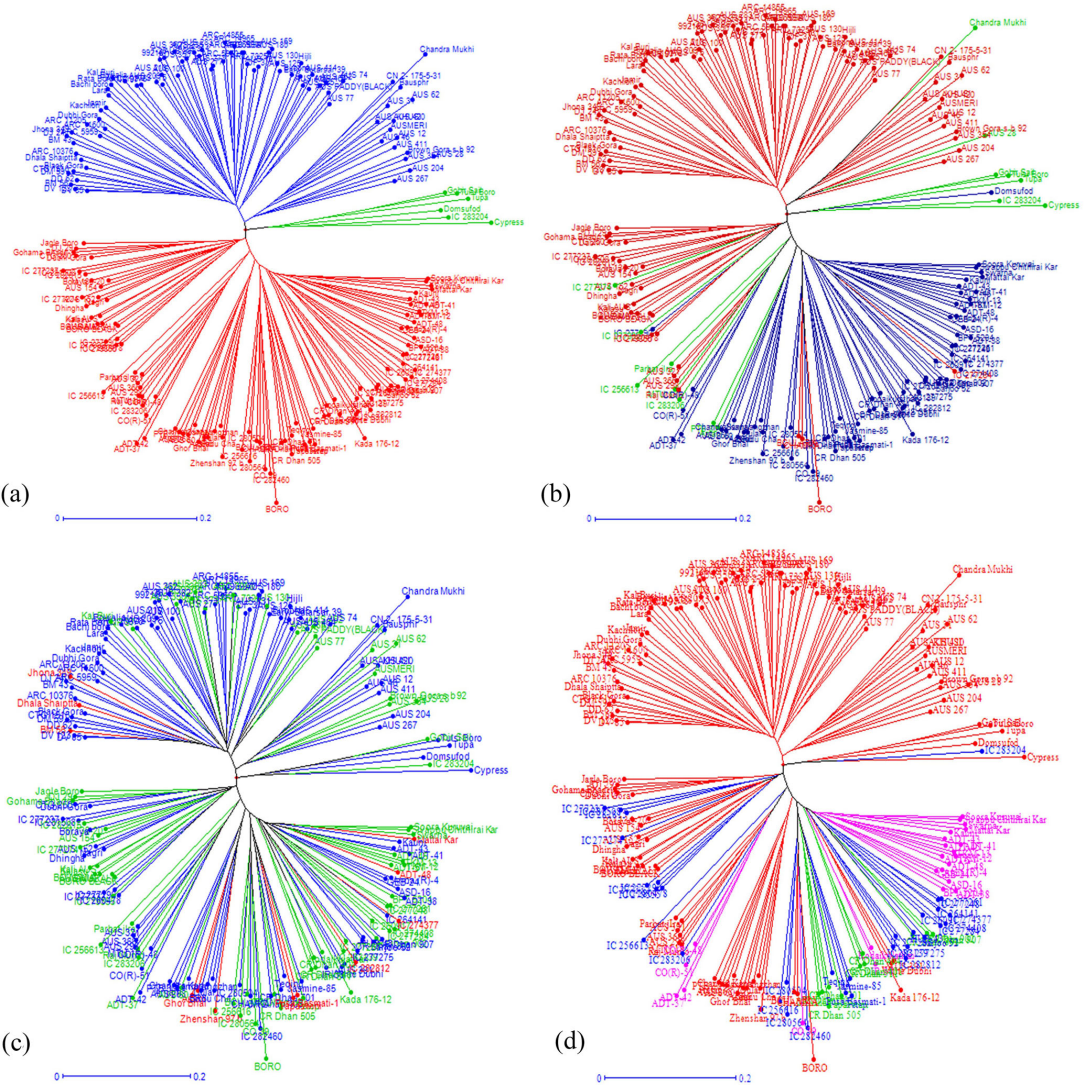
Unrooted tree using unweighted neighbor‐joining (UNJ) method depicting clustering pattern of core population 192 germplasm lines in response to 133 SSRs. (a) Darwin clustered genotypes into three major groups. Cluster 1 (red) comprised 107 genotypes, further subgrouped into clusters 1a and 1b with 82 and 25 genotypes, respectively. Cluster 2 (blue) consists of 79 genotypes and subgrouped into cluster 2a (63 genotypes) and 2b(16 genotypes). Cluster 3 (green) consist of six genotypes. (b) The output of the UNJ tree was utilized to depict the output of population structure analysis by using different colors to understand the similarity of the grouping of genotypes. Genotypes were colored as per the structure analysis, 70 genotypes were categorized as subpopulation 1 (blue color), and 107 as subpopulation 2 (red color), and 15 were considered as admixtures (green color). (c) Additionally, the phylogenetic tree was also utilized to appreciate the nature of disease reaction between and within clusters obtained from the field data using different colors. Moderately resistant (MR) genotypes were coded in red, moderately susceptible (MS) genotypes were in the green, while susceptible (S) genotypes were marked in purple. Genotypes within cluster 1, 49 MR, 49 MS, and nine susceptible genotypes were grouped. Similarly, cluster 2 contained 20 MR, 56 MS, and three susceptible, whereas two MR genotypes and four MS genotypes were grouped in cluster 3. (d) On the other hand, these clusters were marked as per their geographical origin of the genotypes to find the association between the eco‐geographical distribution of genotypes and genetic divergence. The figure 5d highlighted red as *AUS*, NRRI improved varieties in green, Tamil Nadu (TN) improved varieties and landraces in pink, and Uttar Pradesh (UP) improved varieties and landraces in blue.

Cluster analysis was performed to determine the genetic distance and dissimilarity matrix using the unweighted neighbor‐joining (UNJ) method. The Unrooted tree classified genotypes into three major clusters (Figure 5). Cluster 1 (red) consisted of 107 genotypes and further subdivided into clusters 1a and 1b with 82 and 25 genotypes, respectively. Similarly, cluster 2 (blue) consists of 79 genotypes and subdivided into cluster 2a with 63 genotypes and 2b with 16 genotypes. Cluster 3 (green) was the smallest and consisted of six genotypes (Figure 5a). The output of the UNJ tree was also marked in different shades based on their population structure result (Figure 5b), disease reaction (Figure 5c) and eco‐geographical origin (Figure 5d) to appreciate their genetic relatedness.

### 2.5 Analysis of molecular variance (AMOVA), Nei's genetic diversity, and principal coordinate analysis (PCoA)

**FIGURE 6:**
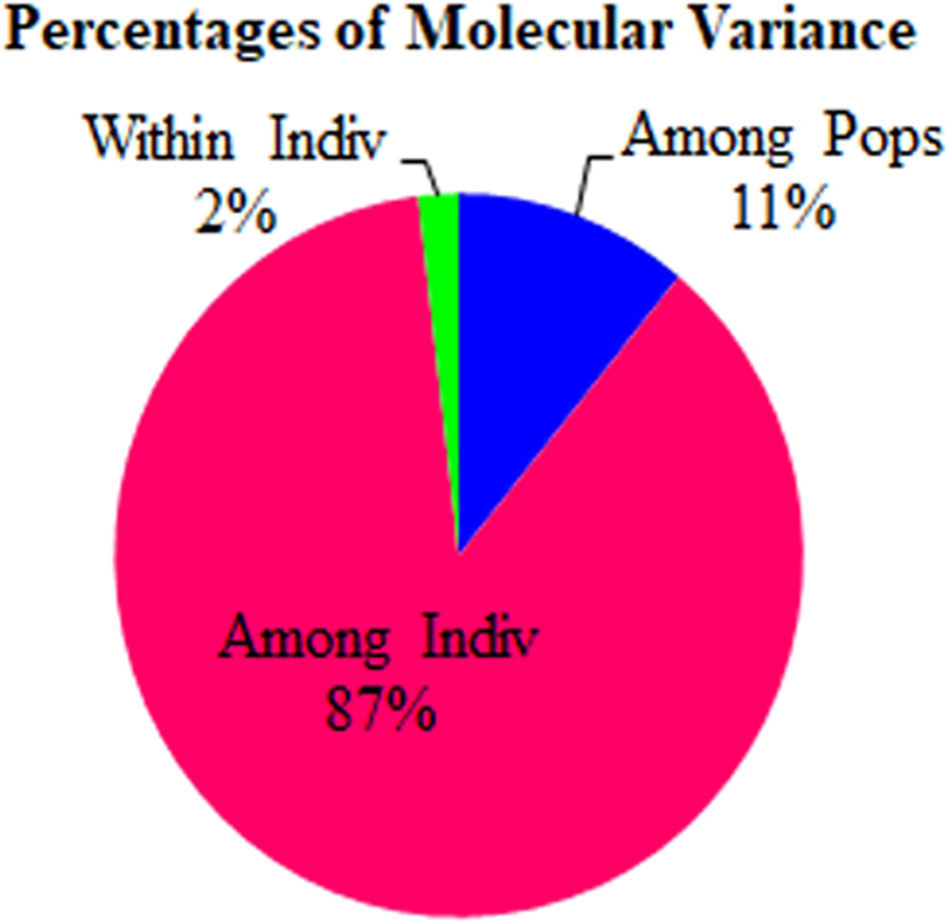
Analysis of molecular variance (AMOVA). AMOVA showed that maximum variation among the individuals, followed by between the populations and within individuals. The genetic variability estimated by the fixation index revealed (Fst = 0.11) indicates the existence of moderate genetic differentiation within the population.

**FIGURE 7:**
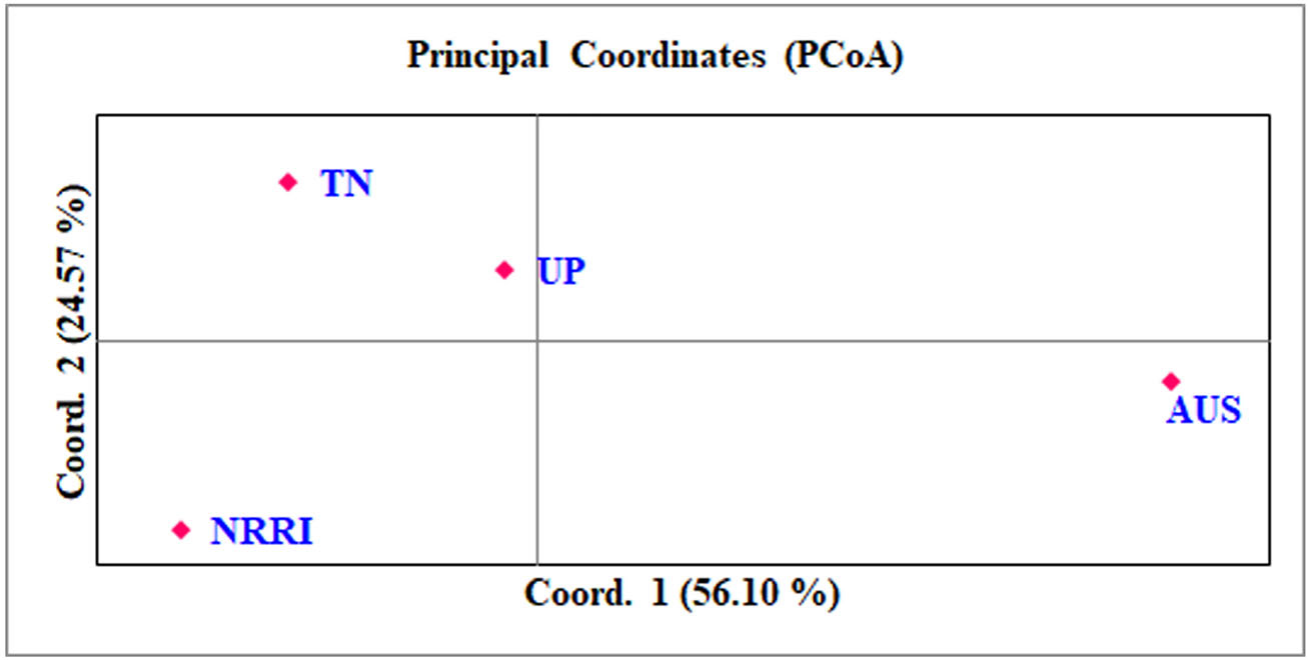
Nei genetic diversity among the assumed subpopulation using principal coordinate analyses (PCoA). The assumed four subpopulations were plotted separately across three quadrants. Indicated the panel population is found to be diverse.

To determine the genetic differentiation among the core population utilized for the present study, the 192 genotypes were further subdivided into four groups (NRRI improved varieties [11], Tamil Nadu [24], Uttar Pradesh [32], and *AUS* [125]) based on their eco‐geographical origin. The analysis of molecular variance (AMOVA) showed that maximum variation (87%) was found among the individuals, followed by between the populations (11%) and within individuals (2%) (Figure 6 and Table 3). The deviation from Hardy–Weinberg's prediction was calculated using Wright's F statistics. The F_IS_ and F_IT_ values for all the loci were 0.979 and 0.981, while F_ST_ was found to be 0.110 between the populations (Table 3). Similarly, the NM value of the assumed population was 2.02. To determine the Nei genetic diversity among the assumed subpopulation, a scatter plot was constructed using principal coordinate analysis (PCoA). The PCoA explained that the first two components accounted for 80.67% of the total genetic variation among the assumed population (Figure 7). Moderately resistant and moderately susceptible genotypes were distributed in all four quadrants, while most of the susceptible genotypes were grouped in quadrant 1. The highest pairwise Nei genetic distance was noticed between population 1 and population 2 (0.216), followed by population 1 with population 3 (0.193) and 4 (0.144).

**TABLE 3:** Analysis of molecular variance (AMOVA) between sub‐populations of the core panel and fixation indices for sheath blight resistance in rice.

Source of variation	df	Sum of squares	Mean sum of squares	Variance components	% of variation
Among populations	3	1050.43	350.14	4.18	11%
Among individual	188	12562.18	66.82	33.04	87%
Within individual	192	139.00	0.724	0.724	2%
Total	383	13751.62		37.95	100%

*F*‐statistics	Value	*p*‐value			
F_ST_	0.110	0.001			
F_IS_	0.979	0.001			
F_IT_	0.981	0.001			

### 2.6 Population structure analysis

**FIGURE 8:**
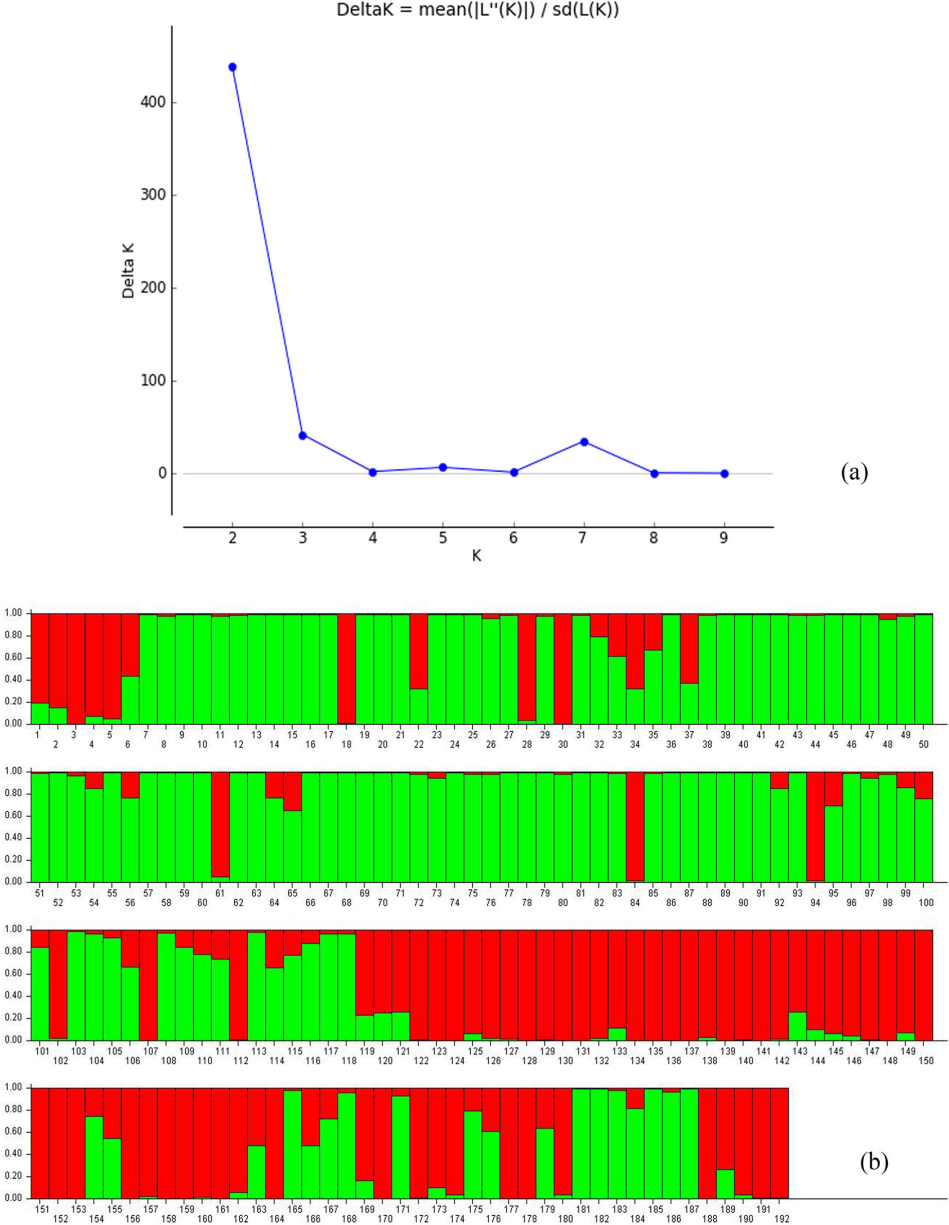
(a) Graph of ∆K value and ad hoc statistics related to the rate of change in the log probability of data between successive K values. (b) Distribution pattern of 192 rice germplasm based on SSRs with respective subpopulations.

The population structure of 192 genotypes was determined using a model‐based approach program Structure 2.3.4. The peak plateau of *ad hoc* measure ∆K was found to be K = 2 with ∆K value of 438 (Figure 8a). The model‐based approach classified the genotypes into two subpopulations (SP1 and SP2), and ancestry threshold of >70% was considered as pure, while <70% considered as admixtures (Figure 8b). Among the 192 genotypes studied, 70 genotypes were placed in SP1, 107 genotypes fitted in SP2, and 15 genotypes were grouped as an admixture. SP1 comprised 29 MR, 32 MS, and nine susceptible genotypes with a population proportion of 36.45% (Table S8). Similarly, 35 moderately resistant, 69 moderately susceptible, and three susceptible genotypes were fitted in SP2 with 55.72% of the population proportion. There was 7.81% membership proportion in admixtures with seven MR and eight MS genotypes. The fixation index (*F*st) values of the two populations were 0.1939 and 0.2823 for SP1 and SP2, respectively. Maximum allele frequency divergence among the populations was obtained in SP1 and SP2 (0.0827) based on net nucleotide distance computed using point estimates of P. The average distance (expected heterozygosity) among the individuals in the panel population was 0.2914 and 0.2764 for SP1 and SP2, respectively.

### 2.7 LD mapping

**FIGURE 9:**
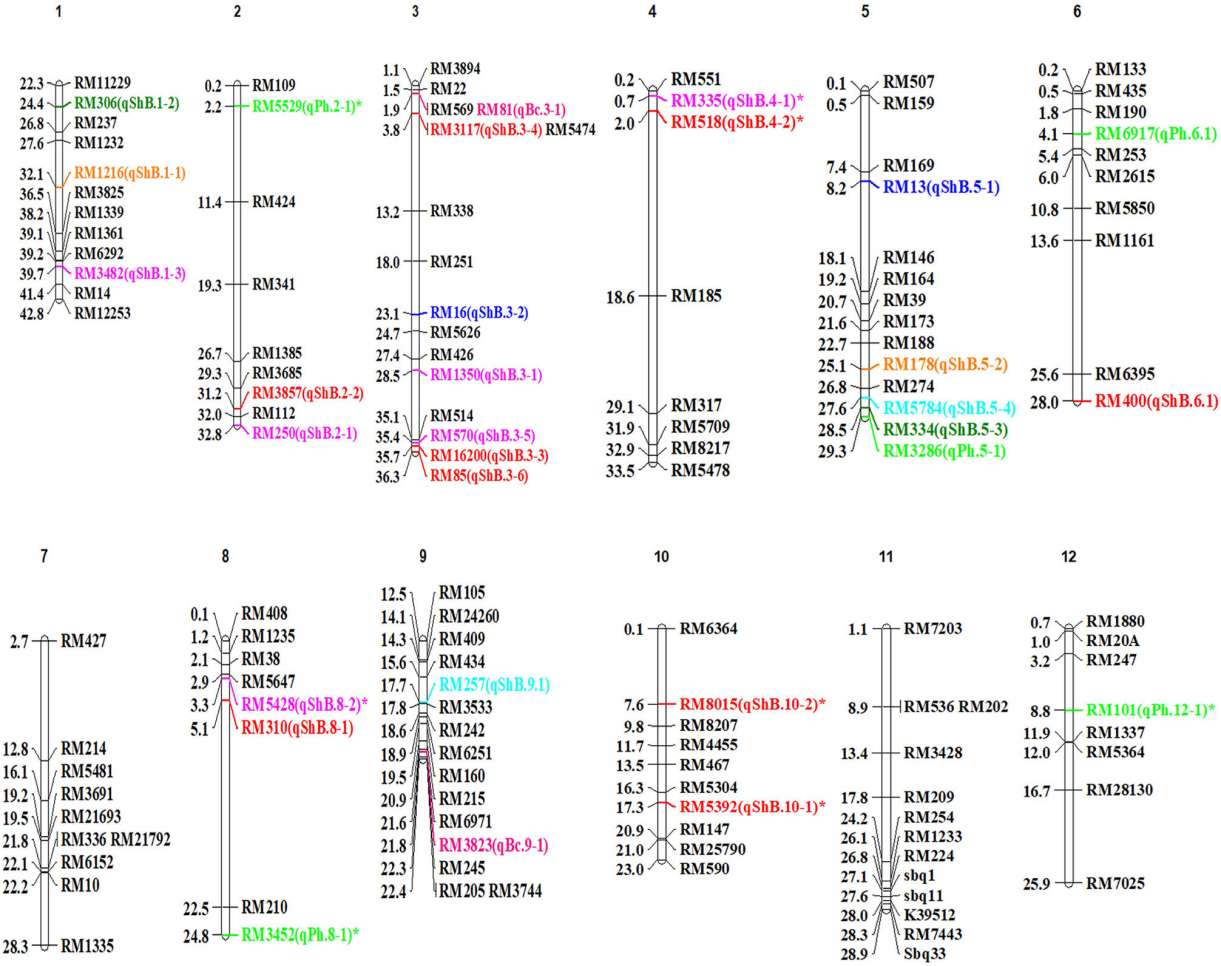
Molecular genetic map on the basis of rice chromosomes on the linkage disequilibrium study, which includes QTLs responsible for sheath blight resistance and other agronomic traits. (Thirty marker loci were associated with sheath blight disease‐related traits and agronomic traits such as plant height and basal leaf sheath color. Markers associated with sheath blight related traits were highlighted using different colors on the basis of number of traits associated. Marker highlighted in dark green associated with six traits, blue five, orange four, light pink three, sky blue two; red, single trait; light green, agronomic traits such as plant height; and dark pink, basal leaf sheath color. *Represents novel QTLs identified in this study.)

**FIGURE 10:**
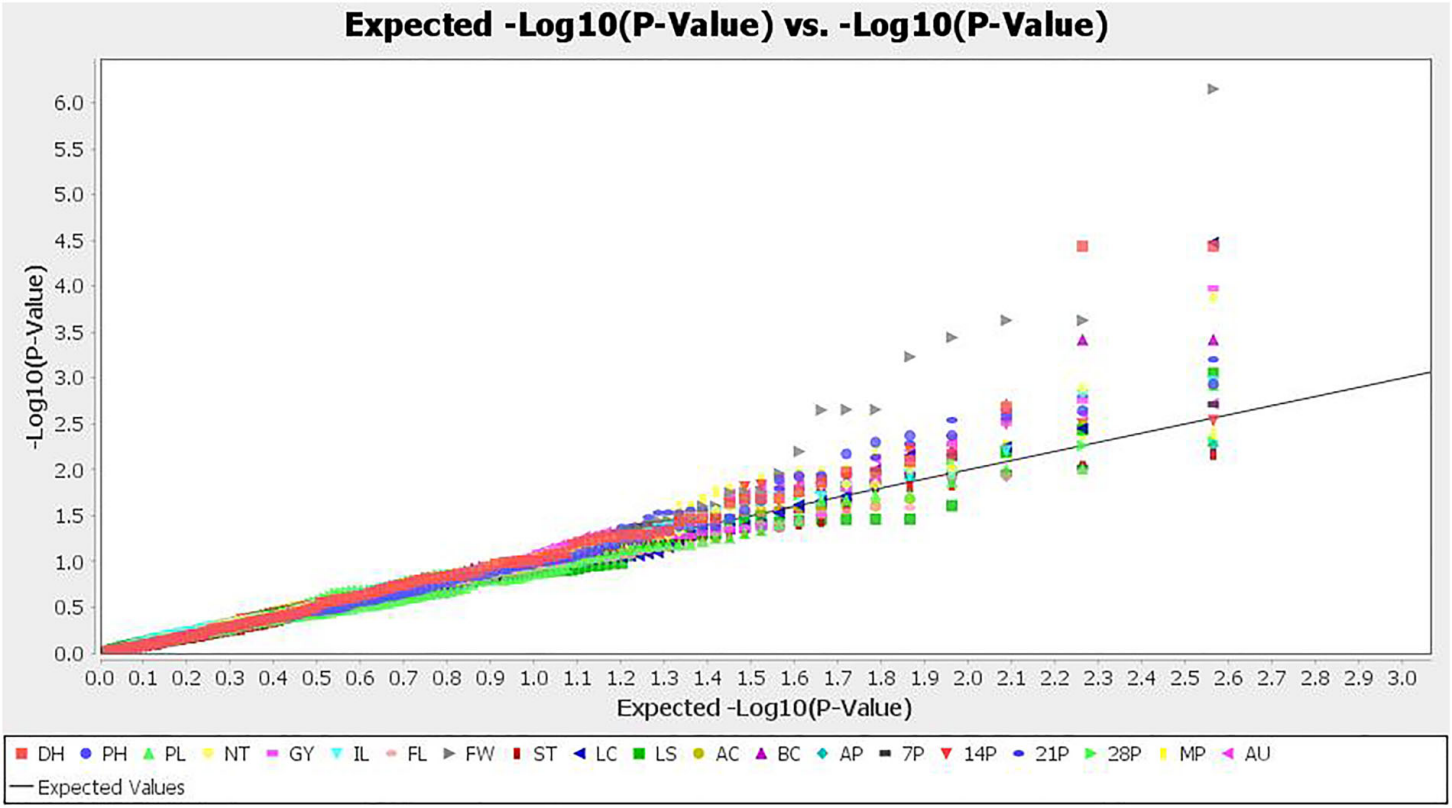
Quantile–quantile (QQ) plot and distribution of SSR markers and traits association. 14P, PDI of 14th day; 21P, PDI of 21st day; 28P, PDI of 28th day; 7P, PDI of 7th day; AC, auricle color; AP, apiculus color; AUDPC, area under disease progress curve; BC, basal leaf sheath color; DH, days to 50% flowering; FL, flag leaf length; FW, flag leaf width; GY, grain yield; IL, internodal length; LC, ligule color; LS, ligule shape; MP, mean PDI; NT, tiller number; PH, plant height; PL, panicle length; ST, culm thickness.

The genetic association analysis of 133 SSRs and sheath blight related and other morphological traits of 192 genotypes was performed using a generalized linear model (GLM) and mixed linear model (MLM/Q + K) model with the program TASSEL version 5.2.63 (Table S9). The association between the SSRs and traits such as PDI of 7, 14, 21, and 28 days after inoculation (DAI), mean PDI, AUDPC, plant height, and culm color was performed for each season (dry season 2019 (season 1) and wet season 2019 (season 2)) and pooled phenotypic data for the association analysis. A total of 8, 14, and 19 SSRs were found to be associated with season 1, season 2, and pooled over the season, respectively (Table 4). Further, both GLM and MLM (Q + K) models showed a total of 30 SSRs were associated across the different seasons of experiments. Among the 30 associations, 23 SSRs were associated with sheath blight related traits, whereas seven SSRs had an association with plant height (5) and culm color (2). Considering both GLM and MLM (Q + K), nine SSRs, namely. RM3482, RM250, RM1216, RM13, RM16, RM178, RM306, RM334, and RM5428, had a highly significant association with sheath blight disease‐related traits such as PDI of 7, 14, 21, and 28 days, mean PDI, and AUDPC across the three conditions (season 1, season 2, and pooled data) (Figure 9). The phenotypic variability of these markers ranged from 7% to 15% and 5% to 13% in GLM and MLM (Q + K), respectively at *p* < .05. The markers RM306 on chromosome 1 and RM334 on chromosome 5 had an association with disease‐related traits such as PDI of 7, 14, 21, and 28 DAI, mean PDI (MP), and AUDPC and explained the phenotypic variability of 9%–14% in GLM and 8%–13% in MLM analysis. Similarly, RM13 on chromosome 5 exhibited phenotypic variability of 10%–14% in GLM, and 5%–8% in MLM associated with PDI of 7, 14, and 21 DAI, MP, and AUDPC. Equally, RM16 on chromosome 3 explained phenotypic variability of 7%–14% in GLM and 5%–7% in MLM and exhibit an association with PDI of 14, 21, and 28 DAI, MP, and AUDPC. Additionally, Q–Q plot further confirmed the significant association of markers with sheath blight‐related and other morphological traits (Figure 10). Markers RM81 and RM3823 were found to be associated with basal leaf sheath color QTLs as detected by both GLM and MLM models with >5.0% phenotypic variability on chromosomes 3 and 9, respectively. The association of other morphological traits such as panicle length, days to 50% flowering, tiller number, grain yield, internodal length, ligule color, auricle color, apiculus color, culm thickness, flag leaf width, and flag leaf length are presented in Table S9.

**TABLE 4:** Association of marker alleles with disease related traits of sheath blight, plant height, and culm color in rice detected by GLM and MLM (Q + K) analyses in a shortlisted panel of 192 rice genotypes.

Year	Sl no.	Trait	Chr	QTL name	Marker	GLM				MLM			
						*F* value	*p* value	*q* value	R^2^	*F* value	*p* value	*q* value	R^2^
Dry season 2019	1	14PDI	2	qShB.2‐1	RM250	12.00	0.000662***	0.001245	0.06	13.02	0.001737**	0.004189	0.07
	2	14PDI	4	qShB.4‐1	RM335	11.96	0.000712***	0.001322	0.07	9.30	6.73E‐05***	0.000394	0.06
	3	14PDI	1	qShB.1‐3	RM3482	13.83	0.000279***	0.000655	0.08	7.84	9.39E‐05***	0.000481	0.05
	4	21PDI	2	qShB.2‐1	RM250	12.51	0.000511***	0.001044	0.06	13.13	0.019725*	0.028883	0.07
	5	21PDI	4	qShB.4‐1	RM335	10.29	0.00164**	0.002345	0.06	10.85	0.00017***	0.000633	0.07
	6	21PDI	1	qShB.1‐3	RM3482	14.33	0.000218***	0.000548	0.08	8.50	1.28E‐05***	0.000105	0.06
	7	21PDI	6	qsShB.6.1	RM400	5.66	0.0194*	0.02025	0.06	6.86	0.035909*	0.047492	0.07
	8	AUDPC	2	qShB.2‐1	RM250	14.25	0.000216***	0.000551	0.07	15.10	0.004224**	0.008659	0.08
	9	AUDPC	1	qShB.1‐3	RM3482	17.50	4.79E‐05***	0.000159	0.10	10.21	1.82E‐05***	0.000124	0.07
	10	MP	10	qShB.10‐1	RM5392	8.07	0.00541**	0.006447	0.07	9.44	0.002272**	0.005176	0.08
	11	MP	8	qShB.8‐2	RM5428	5.14	0.02602*	0.026203	0.06	8.44	0.001215**	0.003114	0.08
	12	MP	10	qShB.10‐2	RM8015	23.26	3.57E‐06***	1.82E‐05	0.14	13.26	5.77E‐07***	7.88E‐06	0.10
	13	PH	6	qPh.6‐1	RM6917	—	—	—	—	6.08	0.369973^ns^	0.388946	0.07
Wet season 2019	14	14PDI	5	qShB.5‐1	RM13	9.66	0.0027**	0.004014	0.12	6.28	0.000258***	0.001587	0.07
	15	14PDI	3	qShB.3‐2	RM16	20.79	1.18E‐05***	7.63E‐05	0.14	9.21	0.000195***	0.001419	0.07
	16	14PDI	1	qShB.1‐2	RM306	12.98	5.51E‐04***	0.001341	0.14	11.06	0.000154***	0.001236	0.13
	17	21PDI	1	qShB.1‐1	RM1216	15.85	1.01E‐04***	0.000413	0.08	12.82	0.000972***	0.002992	0.07
	18	21PDI	5	qShB.5‐1	RM13	11.43	0.00117**	0.002312	0.14	6.18	0.000262***	0.001498	0.08
	19	21PDI	3	qShB.3‐2	RM16	12.85	4.78E‐04***	0.001255	0.09	6.84	0.012582*	0.018639	0.06
	20	21PDI	5	qShB.5‐2	RM178	12.24	7.38E‐04***	0.00161	0.12	6.72	8.13E‐05***	0.001084	0.10
	21	21PDI	1	qShB.1‐2	RM306	7.81	0.00653**	0.00795	0.09	7.71	0.001287**	0.00355	0.08
	22	21PDI	3	qShB.3‐5	RM570	14.68	1.91E‐04***	0.00063	0.09	8.46	0.000317***	0.001689	0.06
	23	28PDI	3	qShB.3‐1	RM1350	5.45	0.02195*	0.023192	0.06	3.87	0.007685**	0.012809	0.05
	24	28PDI	3	qShB.3‐2	RM16	11.81	7.93E‐04***	0.001687	0.08	7.64	6.52E‐05***	0.001043	0.06
	25	28PDI	1	qShB.1‐2	RM306	7.13	0.00924**	0.011009	0.08	8.38	0.000564***	0.00205	0.08
	26	28PDI	5	qShB.5‐3	RM334	6.67	0.01158*	0.01297	0.08	5.43	0.005632**	0.010012	0.07
	27	7PDI	1	qShB.1‐1	RM1216	17.36	4.90E‐05***	0.000235	0.09	17.00	0.004532**	0.009541	0.09
	28	7PDI	2	qShB.2‐2	RM3857	—	—	—	—	4.87	0.091309^ns^	0.104353	0.07
	29	7PDI	5	qShB.5‐1	RM13	12.17	8.32E‐04***	0.001704	0.14	5.44	0.002348**	0.005524	0.06
	30	7PDI	5	qShB.5‐3	RM334	—	—	—	—	6.30	0.112739^ns^	0.127029	0.07
	31	7PDI	3	qShB.3‐3	RM16200	15.23	1.44E‐04***	0.000515	0.09	9.71	0.00325**	0.007026	0.07
	32	7PDI	1	qShB.1‐2	RM306	—	—	—	—	9.87	0.03831*	0.048648	0.10
	33	7PDI	5	qShB.5‐2	RM178	8.35	0.00486**	0.006329	0.09	7.61	0.001329**	0.003429	0.08
	34	AUDPC	1	qShB.1‐1	RM1216	15.50	1.20E‐04***	0.000457	0.08	12.53	0.001314**	0.003504	0.07
	35	AUDPC	3	qShB.3‐1	RM1350	5.32	0.02352*	0.024391	0.06	3.70	0.006797**	0.011569	0.05
	36	AUDPC	5	qShB.5‐1	RM13	9.54	0.00286**	0.004178	0.12	5.26	0.000476***	0.001903	0.06
	37	AUDPC	3	qShB.3‐2	RM16	15.48	1.36E‐04***	0.000498	0.11	7.76	0.000102***	0.001167	0.07
	38	AUDPC	5	qShB.5‐2	RM178	9.85	0.00232**	0.003643	0.10	4.86	0.000472***	0.001988	0.07
	39	AUDPC	1	qShB.1‐2	RM306	9.54	0.00279**	0.004112	0.11	9.98	0.000467***	0.002199	0.10
	40	AUDPC	3	qShB.3‐5	RM570	15.43	1.34E‐04***	0.0005	0.10	8.21	0.000471***	0.002093	0.06
	41	BC	3	qBc.3‐1	RM81	10.68	0.00129**	0.002408	0.05	9.86	0.17744^ns^	0.184353	0.05
	42	BC	9	qBc.9‐1	RM3823	9.20	0.00302**	0.004336	0.08	7.80	0.000904***	0.003015	0.07
	43	MP	1	qShB.1‐1	RM1216	12.16	6.20E‐04***	0.001447	0.07	10.11	0.005086**	0.009688	0.06
	44	MP	8	qShB.8‐1	RM310	—	—	—	—	8.75	0.004792**	0.009583	0.05
	45	MP	3	qShB.3‐1	RM1350	6.92	0.01014*	0.011748	0.08	4.63	0.004886**	0.009534	0.07
	46	MP	5	qShB.5‐1	RM13	9.36	0.00311**	0.004391	0.12	5.13	0.000689***	0.002397	0.06
	47	MP	3	qShB.3‐2	RM16	14.89	1.80E‐04***	0.000604	0.10	7.79	0.000127***	0.001266	0.06
	48	MP	5	qShB.5‐2	RM178	8.08	0.00557**	0.007089	0.09	4.50	0.001172**	0.003472	0.06
	49	MP	1	qShB.1‐2	RM306	9.30	0.00313**	0.004382	0.11	10.71	0.000561***	0.002139	0.11
	50	MP	5	qShB.5‐3	RM334	5.84	0.01793*	0.019434	0.07	5.85	0.011184*	0.017543	0.06
	51	MP	3	qShB.3‐5	RM570	17.42	5.25E‐05***	0.000238	0.11	9.67	6.33E‐05***	0.001265	0.08
	52	PH	5	qPh.5‐1	RM3286	—	—	—	—	3.37	0.022491*	0.031022	0.05
Pooled	53	14PDI	8	qShB.8‐2	RM5428	13.51	0.000422***	0.002075	0.14	6.49	0.014814*	0.028488	0.08
	54	14PDI	5	qShB.5‐1	RM13	11.44	0.00118**	0.003165	0.13	3.85	2.54E‐06***	3.81E‐05	0.06
	55	14PDI	3	qShB.3‐2	RM16	10.33	0.00166**	0.003696	0.07	3.91	0.225228^ns^	0.248413	0.05
	56	14PDI	9	qShB.9‐1	RM257	—	—	—	—	9.10	0.158642^ns^	0.201663	0.05
	57	14PDI	4	qShB.4‐1	RM335	—	—	—	—	9.05	0.003678**	0.011494	0.06
	58	14PDI	5	qShB.5‐3	RM334	5.08	0.02697*	0.027916	0.06	4.80	0.365767^ns^	0.370709	0.05
	59	21PDI	5	qShB.5‐1	RM13	9.11	0.00353**	0.005785	0.10	2.43	2.74E‐05***	0.000256	0.05
	60	21PDI	5	qShB.5‐2	RM178	8.06	0.00565**	0.007937	0.08	4.27	0.012172*	0.024673	0.05
	61	21PDI	3	qShB.3‐6	RM85	16.54	7.13E‐05***	0.000647	0.08	10.30	0.00015***	0.001021	0.06
	62	21PDI	5	qShB.5‐3	RM334	7.14	0.00915**	0.011997	0.08	7.58	0.085251^ns^	0.122958	0.07
	63	21PDI	4	qShB.4‐1	RM335	9.34	0.00267**	0.004566	0.06	9.21	0.000279***	0.001608	0.06
	64	21PDI	9	qShB.9‐1	RM257	10.09	0.00177**	0.003868	0.05	12.14	0.064544^ns^	0.10085	0.07
	65	28PDI	1	qShB.1‐2	RM306	8.16	0.00551**	0.007929	0.09	8.38	0.000564***	0.002487	0.08
	66	28PDI	3	qShB.3‐2	RM16	12.26	0.000638***	0.00215	0.09	7.65	0.0017**	0.00607	0.06
	67	28PDI	5	qShB.5‐3	RM334	6.70	0.01144*	0.01421	0.08	5.43	0.005632**	0.015085	0.07
	68	28PDI	3	qShB.3‐1	RM1350	5.69	0.01926*	0.021645	0.06	3.87	0.005643**	0.014595	0.05
	69	7PDI	4	qShB.4‐2	RM518	9.20	0.00287**	0.004838	0.06	9.97	0.00028***	0.001499	0.06
	70	7PDI	5	qShB.5‐4	RM5784	6.69	0.01105*	0.01402	0.06	6.30	7.91E‐05***	0.000659	0.06
	71	AUDPC	5	qShB.5‐1	RM13	12.95	0.000589***	0.002173	0.14	4.09	2.31E‐07***	1.73E‐05	0.07
	72	AUDPC	3	qShB.3‐2	RM16	19.24	2.4E‐05***	0.000257	0.13	9.58	0.248697^ns^	0.266461	0.06
	73	AUDPC	5	qShB.5‐3	RM334	9.76	0.00249**	0.004664	0.11	10.32	0.048695*	0.083002	0.09
	74	AUDPC	8	qShB.8‐2	RM5428	8.80	0.00395**	0.006299	0.10	4.35	0.009677**	0.021346	0.05
	75	AUDPC	3	qShB.3‐4	RM3117	8.51	0.00409**	0.00635	0.05	8.06	0.008528**	0.019989	0.06
	76	BC	3	qBc.3‐1	RM81	10.30	0.00157**	0.003705	0.05	9.86	0.186789^ns^	0.215526	0.05
	77	BC	9	qBc.9‐1	RM3823	8.97	0.0034**	0.005651	0.07	7.80	0.000904***	0.003768	0.07
	78	MP	8	qShB.8‐2	RM5428	14.63	0.000254***	0.001496	0.15	7.81	5.21E‐06***	5.58E‐05	0.08
	79	MP	3	qShB.3‐5	RM570	23.53	3.26E‐06***	7.69E‐05	0.14	8.55	4.14E‐06***	5.18E‐05	0.06
	80	MP	4	qShB.4‐1	RM335	10.75	0.0013**	0.003264	0.07	8.00	0.000953***	0.003761	0.05
	81	MP	5	qShB.5‐4	RM5784	6.48	0.01239*	0.014919	0.06	6.54	0.023288*	0.0426	0.06
	82	PH	2	qPh.2‐1	RM5529	—	—	—	—	6.54	0.005392**	0.014979	0.06
	83	PH	8	qPh.8‐1	RM3452	12.12	0.000623***	0.002163	0.06	10.90	0.285324^ns^	0.297213	0.06
	84	PH	12	qPh.12‐1	RM101	9.86	0.00206**	0.004191	0.06	8.12	0.116456^ns^	0.164796	0.06

*Note*: *, **, and *** significant at <0.05%, <0.01%, and <0.001% respectively.

Abbreviations: 14PDI, PDI of 14th day; 21PDI, PDI of 21st day; 28PDI, PDI of 28day; 7PDI, PDI of 7th day; AUDPC, area under disease progress curve; BC, basal leaf sheath color; MP, mean PDI; ns, nonsignificant; PH, plant height.

**FIGURE 11:**
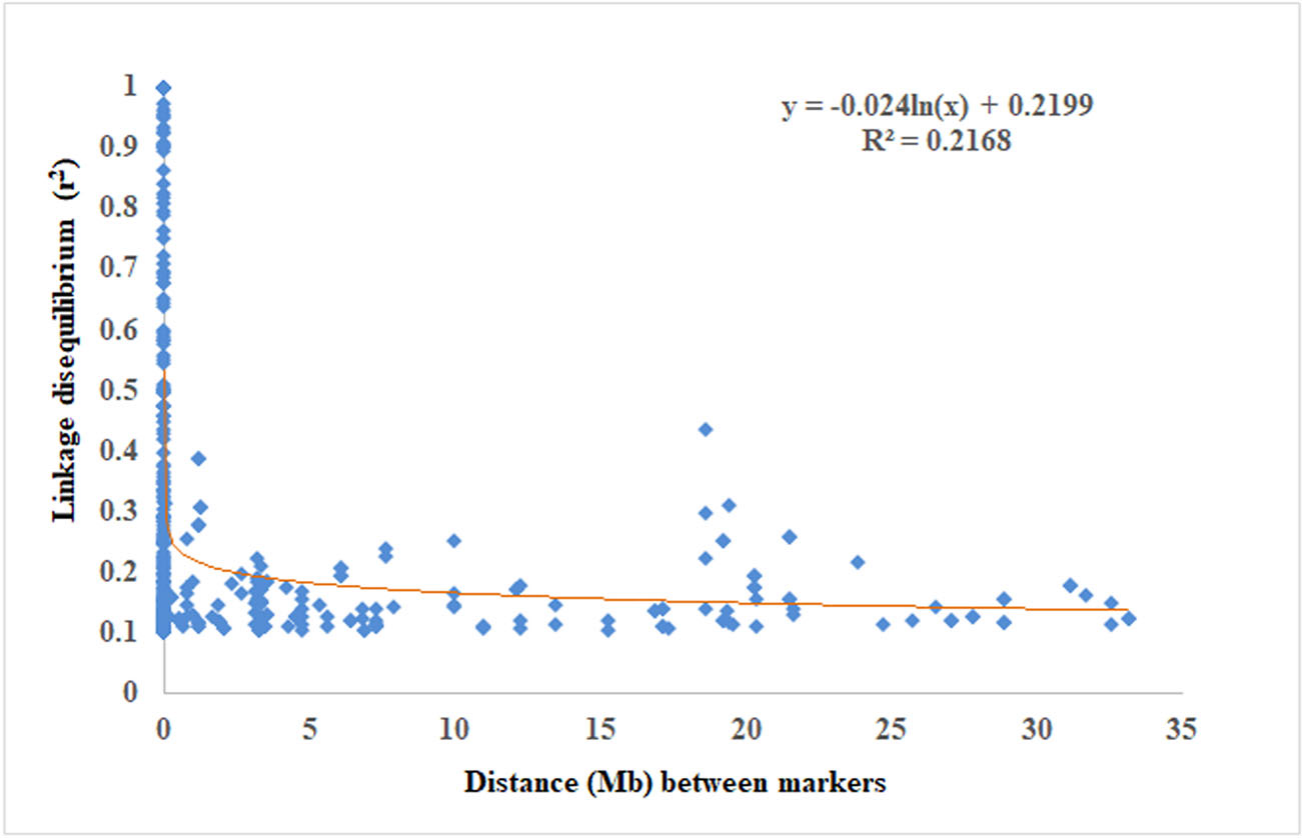
LD decay (*r* ^*2*^) curve plotted against the distance between pairs of loci on chromosomes in rice.

In LD analysis, a total of 133 SSRs were used for the analysis with a maximum of 17,025 pairwise combinations. Among them, 371 (2.17%) marker pairs showed LD (*r* ^2^) of >.10 (Figure 11). LD was subdivided into intra‐chromosomal LD (6086 marker pairs; 35.75%) and inter‐chromosomal LD (10939 marker pairs; 64.25%). The marker pairs were ranged from 190 (chromosome 12) to 2750 (chromosome 3) with an average of 1418.75 per chromosome. Furthermore, marker pairs with *r* ^2^ > .1 were varied from 10 (chromosome 10) to 72 (chromosome 9) with an average of 30.91 per chromosome.

### 2.8 Candidate genes underlying QTLs for traits related to sheath blight disease over the season

Twenty‐three different disease‐related QTLs and seven plant height and basal leaf sheath color QTLs were considered for the identification of candidate genes (Table 5). For the trait plant height, five candidate genes were identified and located on chromosomes 2, 5, 6, 8, and 12. Besides, two genes with locus id *Os03t0122300* and *Os09t0567300* were identified for basal leaf sheath color, while the rest of the candidate genes were identified for sheath blight‐associated traits. Among the candidate genes identified related to sheath blight, two gene (*Os01t0629900* and Os05t0572000) were associated with all six sheath blight‐associated traits, namely, PDI of 7, 14, 21, and 28 DAI, MP, and AUDPC, located on chromosomes 1 and 5, respectively, whereas *Os03t0600600* and *Os05t0246600* were identified to be associated with five sheath blight related traits and presented on chromosomes 3 and 5, respectively. The network analysis of the above candidate genes in the QTL interval region was analyzed using the rice FRIEND database, and it explored the co‐expression pattern of the genes. A total of six candidate genes were taken forward to construct gene network, and their interactions were displayed in Figure S5. Further, the loci information and functions of genes associated with the candidate genes were shown in Table S10. The sum of nodes and edges of co‐expressed genes of the six candidate genes was 36 and 38, respectively. Among the six putative genes, the loci *Os08g0155900* located on chromosome 8 had functionally associated with the locus *Os11g0702100* (similar to class III chitinase homologue *OsChib3Hh*).

**TABLE 5:** Colocalization of significant markers and candidate genes believed to be involved in sheath blight‐related traits, plant height, and culm color identified in the panel population.

Sl. no.	Marker associated	QTL	LOC	Description	Chromosome	QTL location	Trait associated
1	RM1216	qShB.1‐1	Os01t0750400	Leucine‐rich repeat domain‐containing protein	chr01	0.67 Mb right of QTL	7PDI, 21PDI, MP, and AUDPC
2	RM306	qShB.1‐2	Os01t0629900	Similar to blast and wounding‐induced mitogen‐activated protein kinase	chr01	0.71 Mb left of QTL	7PDI, 14PDI, 21PDI, 28PDI, MP, and AUDPC
3	RM3482	qShB.1‐3	Os01t0899800	Pathogenesis‐related transcriptional factor and ERF domain‐containing protein	chr01	0.57 Mb right of QTL	14PDI, 21PDI, and AUDPC
4	RM250	qShB.2‐1	Os02t0769800	Similar to mitogen‐activated protein kinase kinase kinase 1 (EC 2.7.1.‐) (*Arabidopsis* NPK1‐related protein kinase 1). Splice isoform 1S	chr02	0.34 Mb right of QTL	14PDI, 21PDI, and AUDPC
5	RM3857	qShB.2‐2	Os02t0759700	Leucine‐rich repeat 2 containing protein	chr02	0.78 Mb left of QTL	7PDI
6	RM5529	qPh.2‐1	Os02t0154000	Leucine‐rich repeat receptor‐like kinase, positive regulation of the drought stress response and tiller development	chr02	0.75 Mb right of QTL	PH
7	RM1350	qShB.3‐1	Os03t0703200	Leucine‐rich repeat receptor‐like kinase, control of anther development, and pollen fertility under low temperature	chr03	0.23 Mb right of QTL	28PDI, MP, and AU
8	RM16200	qShB.3‐3	Os03t0848700	Coiled‐coil, nucleotide‐binding, and leucine‐rich repeat (CC‐NB‐LRR) protein, resistance to brown planthopper, (Nipponbare: BPH‐susceptible)	chr03	0.02 Mb left of QTL	7PDI
9	RM16	qShB.3‐2	Os03t0600600	Similar to beta‐1,3‐glucanase	chr03	0.70 Mb right of QTL	14PDI, 21PDI, 28PDI, MP, and AUDPC
10	RM311 7	qShB.3‐4	Os03t0184400	Leucine‐rich repeat, plant specific containing protein	chr03	0.67 Mb left of QTL	AUDPC
11	RM570	qShB.3‐5	Os03t0848700	Coiled‐coil, nucleotide‐binding, and leucine‐rich repeat (CC‐NB‐LRR) protein, resistance to brown planthopper, (Nipponbare: BPH‐susceptible)	chr03	0.31 Mb left of QTL	21PDI, MP, and AUDPC
12	RM81	qBc.3‐1	Os03t0122300	Flavanone 3‐hydroxylase, positive regulation of flavonoid biosynthesis, resistance to brown planthopper (BPH)	chr03	0.69 Mb left of QTL	BC
13	RM85	qShB.3‐6	Os03t0860100	Ethylene response factor, transcriptional activator, positive regulation of disease resistance	chr03	0.05 Mb right of QTL	21PDI
14	RM335	qShB.4‐1	Os04t0119800	Leucine‐rich repeat domain‐containing protein	chr04	0.47 Mb left of QTL	14PDI, 21PDI, and MP
15	RM518	qShB.4‐2	Os04t0122000	Leucine‐rich repeat, N‐terminal domain‐containing protein.	chr04	0.70 Mb right of QTL	7PDI
16	RM13	qShB.5‐1	Os05t0246600	Leucine‐rich repeat domain‐containing protein	chr05	0.68 Mb left of QTL	7PDI, 14PDI, 21PDI, MP, and AUDPC
17	RM178	qShB.5‐2	Os05t0515700	Target protein of bacterial effector, Xoo virulence	chr05	0.47 Mb left of QTL	7PDI, 21PDI, MP, and AUDPC
18	RM3286	qPh.5‐1	Os05t0578900	GAGA‐type zinc‐finger transcription factor, organogenesis, regulation of leaf development	chr05	0.50 Mb left of QTL	PH
19	RM334	qShB.5‐3	Os05t0572000	Pathogenesis‐related transcriptional factor and ERF domain‐containing protein	chr05	0.05 Mb right of QTL	7PDI, 14PDI, 21PDI, 28PDI, MP, and AUDPC
20	RM5784	qShB.5‐4	Os05t0566400	Group D mitogen‐activated protein kinase (MAPK), defense responses to BPH, control of excessive defense responses, protection from defense‐response‐related autotoxicity	chr05	0.55 Mb left of QTL	7PDI and MP
21	RM400	qsShB.6‐1	Os06t0667900	Disease resistance protein domain‐containing protein	chr06	0.38 Mb right of QTL	21PDI
22	RM6917	qPh.6‐1	Os06t0199500‐01	Halotolerance protein, flavin mononucleotide (FMN)‐binding protein, regulation of cell division, light‐regulated growth	chr06	0.97 Mb right of QTL	PH
23	RM310	qShB.8‐1	Os08t0193700	Similar to NBS‐LRR disease resistance protein homologue (fragment)	chr08	0.36 Mb left of QTL	MP
24	RM3452	qPh.8‐1	Os08t0504700	Gibberellin‐induced A20/AN1 zinc‐finger protein, negative regulation of GA (gibberellin)‐mediated cell elongation	chr08	0.17 Mb right of QTL	PH
25	RM5428	qShB.8‐2	Os08t0155900	Similar to pathogen‐induced defense‐responsive protein	chr08	0.01 Mb right of QTL	14PDI, MP, and AUDPC
26	RM257	qShB.9.1	Os09t0458300	Leucine‐rich repeat, N‐terminal domain‐containing protein	chr09	0.35 Mb right of QTL	14PDI and 21PDI
27	RM3823	qBc.9‐1	Os09t0567300	Monodehydroascorbate reductase, tolerance to ROS‐induced oxidative stress	chr09	0.80 Mb right of QTL	BC
28	RM5392	qShB.10‐1	Os10t0478300	SG2‐type MYB transcription factor, resistance to fungal and bacterial pathogens	chr10	0.62 Mb left of QTL	MP
29	RM8015	qShB.10‐2	Os10t0195000	Multi‐antimicrobial extrusion protein MatE family protein	chr10	0.59 Mb right of QTL	MP
30	RM101	qPh.12‐1	Os12t0268000	Cytochrome P450 monooxygenase, tryptamine 5‐hydroxylase, regulation of cell death and disease resistance	chr12	0.75 Mb right of QTL	PH

Abbreviations: 14PDI, PDI of 14th day; 21PDI, PDI of 21st day; 28PDI, PDI of 28th day; 7PDI, PDI of 7th day; AUDPC, area under disease progress curve; BC, basal leaf sheath color; MP, mean PDI; PH, plant height; locus name based on Rice Annotation Project Database (RAPDB); QTL location, distance of the loci from the marker.

### 2.9 Identification of rice germplasm with QTLs related to sheath blight traits, culm color, and plant height

A total of 13 rice accessions were showed moderately resistant reaction to sheath blight, including tolerant check Tetep. Among them, 11 genotypes exhibited minimum disease scores in comparison to check genotype. The genotype IC 277248 was on par with the check, while IC283139 had the lowest disease reaction (11.11%) among all the genotypes including check (Tetep—18.51%). Amid the 30 QTLs detected, 11 QTLs (*qPh.2‐1*, *qShB.3‐3*, *qShB.3‐2*, *qShB.3‐6, qShB.4‐1*, *qShB.5‐1*, *qShB.5‐2*, *qShB.8‐1*, *qShB.8‐2*, *qBc.9‐1*, *qPh.12.1*) were found in AUS 283 rice cultivars with the disease score of 16.11 followed by eight QTLs detected in four genotypes, namely. IC 283139 (*qPh.2‐1*, *qShB.3‐2*, *qShB.4‐1*, *qShB.4‐2*, *qShB.5‐5*, *qShB.9.1*, *qBc.9‐1*, *qShB.12*), Sivappu Chithirai Kar (*qShB.4‐1*, *qShB.5‐5*, *qsShB.6.1*, *qShB.8‐1*, *qPh.8‐1*, *qShB.8‐2*, *qShB.9.1*, *qShB.10‐1*), Bowalia (*qShB.1‐2*, *qShB.2‐1*, *qPh.2‐1*, *qShB.3‐2*, *qPh.5‐1*, *qShB.8‐1*, *qBc.9‐1*, *qPh.12.1*) and IC274408 (*qShB.3‐2*, *qShB.4‐1*, *qShB.4‐2*, *qsShB.6.1*, *qShB.8‐1*, *qPh.8‐1*, *qShB.8‐2*, *qPh.12‐1*) with the disease score of 11.11, 13.36, 15.93, and 17.46, respectively (Table 6). The least number of QTLs (*qShB.2‐1*, *qShB.3‐2*, *qShB.8‐1*) was detected in Boro cultivar with the disease score of 17.22. Interestingly, seven QTLs were detected on check variety Tetep (*qShB.2‐1*, *qShB.3‐2*, *qPh.5‐1*, *qShB.8‐1*, *qPh.8‐1*, *qShB.8‐2*, *qBc.9‐1*). Therefore, these accessions could be proposed as donors in the breeding program or to generate MAGIC population to capture increased genetic recombination and genetic variation against sheath blight disease in rice.

**TABLE 6:** List of genotypes identified with their QTL details and disease reaction against sheath blight in rice.

SL. no.	Genotypes	DF (days)	PH (cm)	Yield (gm)	Basal leaf sheath color	PDI (mean)	DR	QTLs
1	IC283139	117	118.905	16.055	3	11.11	MR	qPh.2‐1, qShB.3‐2, qShB.4‐1, qShB.4‐2, qShB.5‐5, qShB.9.1, qBc.9‐1, qShB.12
2	Sivappu Chithirai Kar	126.5	117.7	18.295	1	13.36	MR	qShB.4‐1, qShB.5‐5, qsShB.6.1, qShB.8‐1, qPh.8‐1, qShB.8‐2, qShB.9.1, qShB.10‐1
3	CR Dhan 501	139.5	126.1963	17.4075	1	15.88	MR	qPh.2‐1, qShB.4‐1, qShB.5‐2, qShB.8‐1, qPh.8‐1, qShB.9.1
4	BOWALIA	84	111.5	16.244	1	15.93	MR	qShB.1‐2, qShB.2‐1, qPh.2‐1, qShB.3‐2, qPh.5‐1, qShB.8‐1, qBc.9‐1, qPh.12.1
5	BORO	85.5	113.54	12.57875	1	17.22	MR	qShB.2‐1, qShB.3‐2, qShB.8‐1
6	AUS 283	88	105.235	11.754	1	16.11	MR	qPh.2‐1, qShB.3‐3, qShB.3‐2, qShB.3‐6, qShB.4‐1, qShB.5‐1, qShB.5‐2, qShB.8‐1, qShB.8‐2,qBc.9‐1, qPh.12.1
7	IC277261	107.5	144.635	9.673833	1	18.29	MR	qPh.2‐1, qShB.3‐2, qShB.4‐1, qShB.5‐3, qPh.8‐1, qShB.8‐2
8	CR Dhan 505	137	126.445	15.17	1	17.33	MR	qPh.2‐1, qShB.3‐6, qShB.4‐1,qPh.8‐1
9	AUS MURALI	84.5	104.7017	6.1985	1	18.15	MR	qShB.2‐3, qShB.3‐2, qShB.4‐1, qsShB.6.1
10	Tetep	93.5	127.4652	16.8782	1	18.51	MR	qShB.2‐1, qShB.3‐2, qPh.5‐1, qShB.8‐1, qPh.8‐1, qShB.8‐2, qBc.9‐1
11	IC277274	101.5	142.0875	8.966625	1	18.37	MR	qShB.1‐2, qPh.2‐1, qShB.3‐2, qShB.3‐4, qShB.4‐1, qShB.4‐2, qShB.9.1
12	IC277248	106.5	148.175	10.2915	1	19.17	MR	qPh.2‐1, qShB.3‐2, qShB.3‐4, qShB.4‐1, qPh.8‐1, qShB.8‐2, qBc.9‐1
13	IC274408	123	127.37	19.272	1	17.46	MR	qShB.3‐2, qShB.4‐1, qShB.4‐2, qsShB.6.1, qShB.8‐1, qPh.8‐1, qShB.8‐2, qPh.12‐1
14	Pusa Basmati‐1	108.5	125.117	5.89	1	33.57	S	qShB.3‐2, qShB.3‐6, qPh.8‐1, qShB.9.1, qBc.9‐1, qShB.10‐1,qPh.12‐1
15	Tapaswini	99.5	116.48	15.97	1	36.29	S	qShB.1‐2, qShB.2‐1, qShB.3‐2, qShB.3‐4, qShB.3‐6, qBc.9‐1, qShB.10‐1, qShB.10‐2

Abbreviations: DF, days to 50% flowering; DR, disease reaction; MR, moderately resistant; PDI, percentage disease index; PH, plant height; S, susceptible.

## 3 DISCUSSION

Cultivation of genetically similar rice accessions over a large scale enforces the high selection pressure on the pathogen populations, which results in cultivars becoming highly vulnerable to biotic stresses. Further, climate change and the emergence of new virulent races enable a continuous threat to rice production as well the world's food security (Yadav et al., 2019). Thus, chemical protection measure constrains sustainable progress to keep up the pace with the evolving pathogens (Vasudevan et al., 2016). The genetic potential of conserved accessions (landraces, cultivated varieties, and wild accessions) needs to be fully investigated for the identification of new resistance genes/alleles to impart genetic tolerance in varieties. In the present investigation, we performed genetic diversity and evaluated geographically diverse rice germplasm that is unique, unexplored, and untapped against sheath blight disease of rice grown in seven major states of India and Bangladesh.

Our study found that out of 192 rice accessions studied, none of the variety found immune (complete resistance) to sheath blight, whereas 18 accessions were found to be moderately resistant (MR), 135 were moderately susceptible, and 39 showed a susceptible reaction. Similarly, Goswami et al. (2019) reported that 10 accessions were found to be promising and showed resistance against sheath blight disease out of 261 genotypes evaluated based on PDI and AUDPC values. On the other hand, among the 18 MR genotypes identified, only 11 showed less disease incidence in comparison to tolerant check variety, Tetep (Table 6). The accession IC 283139 showed very less disease incidence relative to other germplasm studied over the three seasons. Similarly, we observed the similar level of tolerance in IC 283139 across six environments (Panda et al., 2023). Dey et al. (2016) also reported that a few genotypes, namely, 10‐3 (introgression line), SM801 (N22 mutant), Wazuhophek, Ngnololasha Phougak, and Gumdhan (Gall midge biotype differential), were moderately resistant to sheath blight disease based on 3‐year field screening analysis. In our repeated testing of genotypes over the season, the previously identified moderately resistant check varieties such as Teqing (Li et al., 1995; Pinson et al., 2005; Yin et al., 2009) and Jasmine 85 (Liu et al., 2009; Pan et al., 1999; Zuo et al., 2013) were found to be moderately susceptible to sheath blight pathogen. Furthermore, our finding was in agreement with Dey et al. (2016), who has reported that Jasmine 85 showed moderately susceptible reaction to *R. solani*, and Bal et al. (2020), who reported that Teqing showed susceptible reaction to *R. solani*. These findings suggest that pathotype, cultivar, and environmental interaction determines the resistance reaction (Naveenkumar et al., 2022).

### 3.1 Genetic diversity

The genetic construction of diverse rice accessions can be analyzed by using distance‐based and model‐based clustering approach (Ngangkham et al., 2018; Pradhan et al., 2020; Yadav et al., 2017). To our knowledge, our work might be the first report from India and the second in the globe, wherein sheath blight trait‐linked SSRs were utilized to evaluate the population structure and genetic diversity in 192 germplasm lines of Indian rice accessions. Further, a complete analysis of sheath blight trait‐linked microsatellite markers in a diverse collection of rice would be helpful for breeders in designing breeding strategies for sheath blight disease resistance. In the present investigation, 133 SSR loci generated 366 alleles with a mean value of 2.781 and a mean PIC value of about 0.383. The comparison of our findings with earlier reports in rice suggested similar observations with population panel‐based variations. For example, Yadav et al. (2019) found that the average number of the allele was 1.76 with 0.25 mean PIC value of Indian rice landrace collection. Similarly, Pradhan et al. (2020) also observed average alleles per locus was 2.44, with one to six alleles per loci, and PIC values range from 0.000 to 0.671 with the mean of 0.355 in the collection of rice accessions including landraces and improved varieties. The average major allele frequency value was found to be 0.642, whereas the gene diversity value was 0.443 per locus. This is in agreement with previous reports of Anandan et al. (2016), Yadav et al. (2019), Pradhan et al. (2020), Chettri et al. (2021), and Anandan et al. (2022). Further, heterozygosity was found to be low with an average of 0.013 per loci, and average inbreeding coefficient value per primer was 0.972, which is in agreement with previous reports on rice (Anandan et al., 2016; Nachimuthu et al., 2015). Based on SSR genotypic data, a distance‐based neighbor‐joining (NJ) tree categorized the core population of 192 genotypes into three major clusters. Among the three clusters, the MR accessions were grouped in cluster 1, while moderately susceptible genotypes were categorized in cluster 2. Our finding is in agreement with Yadav et al. (2019) who reported that the majority of resistant and susceptible genotypes were grouped into distinct clusters screened against the disease rice leaf blast. Concurrently, Susan et al. (2019) also found that resistant cultivars were grouped in unique clusters in response to blast disease of rice. Besides, the grouping of NJ‐based clustering was found to be similar to the structure‐based subpopulation group. The program STRUCTURE model‐based grouping categorized the panels into two subpopulations considering the threshold of 70%. Earlier studies on the genetic structure of populations of rice reported a varied number of subpopulations 2–8 (Anandan et al., 2016; Jia et al., 2012; Pradhan et al., 2020) with varied level of the threshold between 55% and 80% to determine the genotype relatedness to a specific subpopulation (SP). In the present study, SP1 consists of 70 accessions, followed by 107 belonging to SP2, and 15 were categorized as an admixture. The use of trait‐linked SSRs in the present study might be the reason for the smaller number of subgroups identified by the population structure analysis, but they were very well differentiated based on sheath blight disease reactions. Similarly, Yadav et al. (2017) and Susan et al. (2019) reported that the STRUCTURE and PCoA scatter plot clearly grouped the panel population into resistant and susceptible subpopulations evaluated against rice leaf blast disease.

AMOVA estimates the molecular variance within the individuals in the panel population. In our study, the highest percentage of the proportion of variation was found among the individuals (87%), the lowest variant existed between the individuals (11%), and the least was observed (2%) within the individuals. There is enough number of reports in agreement with our observation for biotic stress response in rice (Yadav et al., 2017, 2019) and abiotic stress (Ngangkham et al., 2018; Pradhan et al., 2020). The genetic variability estimated by the fixation index revealed that 0.11 (Fst) indicates the existence of moderate genetic differentiation within the population and inbreeding coefficient F_IS_ and F_IT_ values were found to be 0.979 and 0.981, respectively. Conversely, Anandan et al. (2016) reported little divergence existed between rice subpopulation (Fst = 0.016). The moderate level of genetic differentiation in the present study might be due to the collection of large number of landraces from different provinces of India and Bangladesh.

### 3.2 Marker and rice sheath blight resistance trait associations

Association mapping becomes the most effective tool in mapping the genes of interest by utilization of *ex situ* conserved natural genetic diversity of crop germplasm resources. With regard to the QTL identification for sheath blight resistance, earlier researchers utilized the RLH as sheath blight trait for QTL identification in biparental and association mapping panel (Liu et al., 2014; Taguchi‐Shiobara et al., 2013; Wen et al., 2015; Yadav et al., 2015). Among them, very few stable QTLs were reported across location/seasons (Han et al., 2003; Pinson et al., 2005; Zuo et al., 2013), unlikely no reports available on QTL cloned for *R. solani* resistance in rice. Therefore, in the present investigation, we have used the PDI of different time intervals such as 7th, 14th, 21st, and 28th DAI of *R. solani* for QTL identification. Further, AUDPC generated using this different day's interval was also used to detect the sheath blight responsive QTLs. To our best knowledge, this is the first report for sheath blight QTL identification in rice using PDI and AUDPC traits to detect the QTLs associated with sheath blight resistance in rice.

In our GWA study, we found 30 QTLs (markers) that had a highly significant association with sheath blight disease resistance (Table S11). Among them, eight QTLs, namely, *qPh.2‐1*, *qShB.4‐1*, *qShB.4‐2*, *qPh.8‐1*, *qShB.8‐2*, *qShB.10‐1*, *qShB.10‐2*, and *qPh.12‐1*, were novel QTLs related to sheath blight resistance, which has not been reported in earlier studies. Among the novel QTLs identified, three QTLs such as *qPh.2‐1*, *qPh.8‐1*, and *qPh.12‐1* were associated with plant height that has positive correlation with sheath blight‐resistant traits. The markers associated with these plant height QTLs were associated with blast resistance genes such *pi‐Da(t)*, *pi55 (t)*, and *Pi61 (t)*, respectively (He et al., 2004, 2017; Lei et al., 2013; Shi et al., 2012). The QTL *qPh.2‐1* identified in this study is located approximately 9.1 Mb away on chromosome 2 from the sheath blight QTL reported by Liu et al. (2009). Further, on chromosome 8, QTL *qPh.8‐1* and *qShB.8‐2* were 1.85 and 2.3 Mb away from the QTL reported by Yadav et al. (2015) and Channamallikarjuna et al. (2010), respectively, while *qPh.12‐1* mapped on chromosome 12 was 17.09 Mb away from the QTL associated with number of tillers with lesion reported by Taguchi‐Shiobara et al. (2013). The PDI of the 7th day was positively associated with three QTLs *qShB.2‐2*, *qShB.3‐3*, and *qShB.4‐2* on chromosomes 2, 3, and 4, respectively, controlling sheath blight resistance during the early stage with phenotypic variation (PV) ranged from 6% to 9% in GLM and 6% to 7% in MLM analysis. There are putative resistance‐related candidate genes located within these three QTLs. Notably, leucine‐rich repeat genes (*Os02t0759700*, *Os03t0848700*, and *Os04t0122000*) were located at 0.78 Mb left of *qShB.2‐2*, 0.02 Mb left of *qShB.3‐3* and 0.70 Mb right of *qShB.4‐2*, which might play a role in imparting immunity (Ng & Xavier, 2011) against *R. solani*. Besides, two QTLs *qShB.2‐2* and *qShB.3‐3* was found to be colocalized with the previously reported QTLs associated with sheath blight‐related traits (Sharma et al., 2009; Taguchi‐Shiobara et al., 2013). Added to the above, leucine‐rich repeat gene was also found located at 0.35 Mb right of the QTL *qShB.9‐1* on chromosome 9 associated with 14th and 21st day PDI with PV of 5% and 7% in GLM and MLM, respectively. Furthermore, this QTL was found to be overlapped with the QTLs (*qSBR9* and *qSBR9.1*) reported by Channamallikarjuna et al. (2010) and Yadav et al. (2015). The QTLs *qShB.3‐6* and *qShB.6‐1* showed a stronger association with PDI on the 21st day with a PV of 8% and 6% in GLM and 6% and 7% in MLM analysis. The resistance imparted by these QTLs might be due to the locus Os03t0860100 being at 0.05 Mb right of *qShB.3‐6* on chromosome 3 and Os06t0667900 at 0.38 Mb right of *qShB.6‐1* on chromosome 6 responsible for positive regulation of disease resistance (Table 5).

The novel sheath blight disease‐resistant QTL (*qShB.10‐1*, *qShB.10‐2*) related to mean PDI on chromosome 10 was observed to have PV of 7%–14% and 8%–10% in both GLM and MLM analysis in response to RM5392 and RM8015, respectively (Table 6). MYB transcription factor and MatE family protein associated with resistance against fungal and bacterial pathogens were found to be located within 0.60 Mb on left and right of *qShB.10‐1* and *qShB.10‐2*, respectively. The AUDPC, which gives details on quantitative summary of disease intensity over time, had a QTL *qShB.3‐4* on chromosome 3 with the PV of 5% and 6% in both GLM and MLM analysis respectively with the *p*‐value at <.01%. At 0.67 Mb left of *qShB.3‐4*, the gene leucine‐rich repeat (Os03t0184400) might be linked with sheath blight resistance. Sharma et al. (2009) also found the marker RM3117 associated with sheath blight disease score on chromosome 3 with 6.4% PV. Besides, three significantly associated QTLs (*qShB.1‐1*, *qShB.1‐2*, and *qShB.1‐3*) on chromosome 1 was identified for multiple sheath blight traits, namely, PDI of 7, 14, 21, and 28 DAI, MP, and AUDPC with PV of 7%–14% and 5%–13% in GLM and MLM, respectively. In support of this, Sharma et al. (2009) and Channamallikarjuna et al. (2010) also reported the association of marker loci RM1216, RM3482, and RM306 with sheath blight resistance and plant height on chromosome 1. Higher PV was found with these markers to be the stronger association with the nearest candidate genes associated with these QTLs, that is, leucine‐rich repeat domain‐containing protein at 0.67 Mb on the right side of the locus RM1216, pathogenesis‐related transcriptional factor and ERF domain‐containing protein at 0.57 Mb on right side of the locus RM3482, and blast and wound‐induced mitogen‐activated protein kinase associated with the locus RM306 at 0.71 Mb on the left side of the QTL (Table 5).

The QTLs *qShB.5‐1* and *qShB.5‐3* registered PV of 7%–14% (GLM) and 5%–10% (MLM) for PDI and AUDPC was found associated with SSR genomic region of RM13 and RM334 on chromosome 5. The nearest candidate genes such as leucine‐rich repeat domain‐containing protein (0.68 Mb away left side from the marker RM13) and pathogenesis‐related transcriptional factor and ERF domain‐containing protein (0.05 Mb away right angle from the locus RM334) may involve in the sheath blight disease resistance (Table 5). Previously, Liu et al. (2009) also detected these marker loci on chromosome 5 contributing to sheath blight disease resistance with 5.4% PV.

Knowing the effect of favorable alleles on the traits of interest is one of the crucial parameters to strengthen the performance of modern rice cultivars by introgressing through MAS (Ngangkham et al., 2018). In the present experiment, elite alleles imparting resistance against sheath blight disease in rice were identified (Table S12) and that would be useful in developing rice varieties with durable disease resistance. Interestingly, the following 11 elite alleles associated with markers, namely, RM250 (Chr 2), RM1350 (Chr 3), RM16 (Chr 3), RM3117 (Chr 3), RM335 (Chr 4), RM13 (Chr 5), RM178 (Chr 5), RM5784 (Chr 5), RM400 (Chr 6), RM310 (Chr 8), and RM5428 (Chr 8), were found to enhance the disease resistance for sheath blight tolerance (Figure 9). Besides, several researchers (Che et al., 2003; Liu et al., 2009; Sharma et al., 2009; Taguchi‐Shiobara et al., 2013; Yadav et al., 2015) have observed the above said markers to have association with biotic resistance in rice. The network analysis of the locus *Os08g0155900* by the rice FREND database revealed that the co‐expression pattern of the genes was highly expressed in class III chitinase, which is responsible for disease resistance. Similarly, the marker RM5428 (chromosome 8) from this study suggested that by controlling traits of sheath blight resistance, also the QTLs were associated with the candidate gene OsDR10 and function of this gene is pathogen‐induced defense responsive protein. This report on identification of elite alleles and their candidate genes for disease tolerance would be the first report for sheath blight disease, and it might lead researchers to identify reliable resistance sources having elite alleles for the development of durable resistance.

## 4 CONCLUSION

Assessing large number of rice germplasm for resistance against sheath blight disease brings valuable information. We conclude from our experiment that sheath blight disease resistance should be studied on 21st and 28th days' post inoculation of *R. solani*. The germplasm utilized to study diversity patterns by subjecting trait‐linked microsatellite markers has classified tolerant and susceptible genotypes. The result of dissimilarity analyses and distance‐based approach was in corroboration with model‐based structure analysis. In the present study, we identified 30 QTLs that had a highly significant association with sheath blight disease resistance. Among them, eight QTLs, namely, *qPh.2‐1*, *qShB.4‐1*, *qShB.4‐2*, *qPh.8‐1*, *qShB.8‐2*, *qShB.10‐1*, *qShB.10‐2*, and *qPh.12‐1*, were novel QTLs related to sheath blight resistance. Notably, 13 LRR genes on eight chromosomes (1, 2, 3, 4, 5, 8, 9, and 12) were found to have associated with sheath blight disease resistance in rice that would be useful in developing varieties with durable sheath blight disease resistance in rice. However, these QTLs need to be further validated before they could be utilized for marker‐assisted breeding in rice. The newly identified donors from this study possessing favorable alleles could be used for imparting resistance against sheath blight disease in rice after functionally characterizing them.

## 5 MATERIALS AND METHODS

### 5.1 Planting materials

A total of 330 diverse rice (*Oryza sativa* L.) accessions encompassing cultivated varieties and landraces of *indica* and *aus* (Table S1) were collected from the Gene Bank, ICAR‐National Rice Research Institute (NRRI), Cuttack, Odisha; Department of Genetics and Plant Breeding, Banaras Hindu University (BHU), Varanasi, Uttar Pradesh; and Department of Rice, Tamil Nadu Agricultural University, Coimbatore. The agro‐morphological trait and source of seeds are presented in Table S1.

### 5.2 Phenotyping experiment 1

The 330 diverse rice genotypes were evaluated for sheath blight resistance during the wet season (*Kharif*) 2018 at the experimental farm, BHU (28.18^0^ N, 38.03^0^ E, and 75.5 masl), Varanasi. The experiment was laid out in a 156 m^2^ plot following an augmented block design with 13 blocks. Two seedlings per hill were planted with a spacing of 20 and 15 cm between and within rows, respectively. The moderately resistant (Tetep, Jasmine 85, and Teqing) and susceptible checks (Pusa Basmati‐1 and Tapaswini) were planted in each block in replicated mode with 25 test entries per block. The recommended dose of fertilizers such as 120:60:60 kg NPK ha^−1^ was applied as basal and top dressing. The highly virulent strain of *R. solani* (MTCC12227) was obtained from the Department of Mycology and Plant Pathology, Institute of Agricultural Sciences, Banaras Hindu University, Varanasi, India. During tillering stage of the crop, from the top second leaf sheath was placed with 4‐day‐old immature sclerotia of *R. solani* (AG1‐IA) grown on potato dextrose agar at 26 ± 2°C and tied with wet cotton to provide moisture for the initial pathogen development (Goswami et al., 2019; Panda et al., 2023). Based on the phenotypic assessment of sheath blight, we have developed a core population set consisting of 192 rice genotypes representing the diversity of the whole population that includes genotypes of S, MS, MR, and R and genotypes from each cluster (Figure 2) following the normal distribution used for association mapping studies. The core panel includes the cultivated varieties and landraces with known tolerant (Tetep, Jasmine 85, Teqing) and susceptible checks (Pusa Basmati 1 and Tapaswini) of sheath blight.

### 5.3 Phenotyping experiment 2

The core population developed from experiment 1 was evaluated in the experimental plot of NRRI (20^o^27′09″ N, 85^o^55′57″ E, 26 masl), Cuttack, during the dry (Rabi) and wet (Kharif) seasons of 2019. Initially, the seeds were heat‐treated to break the seed dormancy by keeping them in the hot‐air oven for 45 h at 50°C. Seedlings were raised in an elevated nursery bed and 30 days after sowing, and the healthy seedlings were carefully uprooted from nursery beds and transplanted into the main field along with the checks. The experiment was laid out in a 175 m^2^ area in a randomized block design with three replicates. Two seedlings per hill were planted in a 1.5 m row length of 20 cm between rows and 15 cm between plants. The recommended dose of fertilizers was applied as a basal and top dressing (120:60:60 kg NPK ha^−1^). At the tillering stage of the crop, immature sclerotia of the *R. solani* (AG1‐IA) (MK478903.1) was inoculated to infuse disease in the plants (Goswami et al., 2019; Panda et al., 2023). Several phenotypic parameters related to sheath blight such as plant height (cm), number of tillers per plant, days to 50% flowering, panicle length (cm), flag leaf width (cm), flag leaf length (cm), internodal length (cm), culm thickness (mm), basal leaf sheath color, ligule color, auricle color, apiculate color, and grain yield were observed at appropriate stages following IRRI SES score. Besides, disease severity was monitored at weekly intervals such as 7, 14, 21, and 28 DAI of the pathogen in each genotype. The RLH was estimated using the following formula described by Sharma and Teng (1990).

$$\mathrm{RLH}=\left(\text{maximum height}\;\mathrm{at}\;\text{which lesion appear}/\text{plant height}\right)\times 100$$

Disease scoring (0–9 scale) given by IRRI (2013) was used for the disease assessment, and the PDI was estimated as follows Baker and Wheeler (1970).

$$\mathrm{PDI}=\left(\mathrm{sum}\;\text{of}\;\mathrm{all}\;\text{rating}/\left(\text{total number of observations}\times \text{maximum rating scale}\right)\right)\times 100$$

The AUDPC was calculated by using the corresponding disease severity percentage of each disease score taken four times at 7 days' interval. AUDPC was generated by using the formula given by Shaner (1977).

$$\text{AUDPC}=\sum_{i=1}^{n-1}\left\{\left[\left(X_{i+1}+X_{i}\right)\right]/2x\left(t_{i+1}-\mathrm{ti}\right)\right\}$$

where X_i_ is the disease index expressed as a proportion at the *i* ^th^ observation, *t*_i_ is the time (days after planting) at the *i* ^th^ observations, and *n* is the total number of observations.

### 5.4 Genotyping of the core panel

#### 5.4.1 DNA isolation and quantification

Young leaf samples were collected from 21‐day‐old seedlings of 192 genotypes of the panel population. Total genomic DNA of all the genotypes was isolated using an automated tissue homogenizer (1600 MiniG®, NJ) in CTAB extraction buffer (100 mM Tris–HCl pH 8, 20 mM EDTA pH 8, 1.3 M NaCl, 2% CTAB) and chloroform‐isoamyl alcohol extraction followed by RNAse treatment and ethanol precipitation (Murray & Thompson, 1980). DNA quality and concentration were estimated by using 0.8% agarose gel electrophoresis. Further, sample DNA was diluted to 30 ng/μL. For the present study, 103 sheath blight‐linked microsatellite markers from previously published reports of biparental mapping and 30 SSR primers were selected in such a way that it has distributed across all the rice chromosomes to illustrate the diversity (Table S2).

#### 5.4.2 PCR amplification and visualization of markers linked to sheath blight resistance

The PCR amplification was performed in a thermocycler (Bio‐Rad) using 10 μl of PCR mixture, which contained 15 ng of genomic DNA, 1× PCR buffer, 200 μM dNTP mix, 4 picomoles of each forward and reverse primers, 2 mM of MgCl2, and 0.2 U of Taq DNA polymerase. In thermocycler, initially, the reaction mixture was denatured for 5 min at 94°C followed by 35 cycles of denaturation for 1 min at 94°C, annealing of 1 min at 48–62°C, and extension of 2 min at 72°C and followed by final extension for 10 min at 72°C. Agarose gel of 3% containing 0.8 μg/ml ethidium bromide was used for electrophoresis. Aliquots of 8 μl PCR amplification product were loaded in the 3% agarose gel containing 0.8 μg/ml ethidium bromide, which was electrophoresed in 1× TBE (pH 8.0) at 100 V (2.5 V/cm) for 3 h. The size of amplicons was determined by using a 50‐bp DNA ladder. Later, gel with amplified products was visualized and documented under ultraviolet (UV) light source in a gel documentation system (Bio‐Rad).

#### 5.4.3 Genetic diversity parameters, population structure, and LD mapping

Allele size of marker data for each genotype was generated based on the presence or absence of band and marker combination. Allele number, gene diversity, major allele frequency, the PIC, and heterozygosity for each SSR locus were determined by using PowerMarker software version 3.25 (Lu et al., 2005). In addition, the allelic data were subjected to the Nei coefficient for dissimilarity index with a bootstrap value of 1000 and constructed the unrooted UNJ tree (Nei, 1972) using Darwin 6.0 software (Perrier & Jacquemoud‐Collet, 2006). Further, AMOVA was generated using GenAlex 6.502 program to describe the presence of molecular variance components within and between the population differentiations among the four assumed subpopulations and population assignment (Peakall & Smouse, 2012) and to calculate the F statistics comprising the deviations from Hardy–Weinberg expectation across the population (F_IT_) and within population (F_IS_) and correlation of alleles between the subpopulation (F_ST_). Besides, PCoA was determined using the binary data of markers for each genotype, and Nei's gene diversity statistics and the number of migrants (NM) between the assumed subpopulations were generated using GenAlex version 6.502.

Population structure analysis was determined by using the Structure 2.3.4 program to find the genetic structure of the given population (Pritchard et al., 2000). We ran the software with the model parameter based on admixture and correlated allele frequency with the burn‐in period of 1,00,000, followed by 1,00,000 Markov chain Monte Carlo (MCMC) replications. The number of subgroups (K) in the clusters was generated by simulating the different K values ranging from 1 to 10 with 10 independent runs for each K value. To find out the true K value, ad hoc statistics ∆K was followed (Evanno et al., 2005) using Structure Harvester 0.6.94 version (Earl & vonHoldt, 2012). To determine the genetic association for sheath blight resistance traits with primers used, two statistical models, that is, GLM and MLM in TASSEL version 5.2.63 (Bradbury et al., 2007), were used. LD plot was constructed using measured *r* ^2^ values between the markers and plotted against the nucleotide difference between the marker pairs. False discovery rate (FDR) was performed to obtain *q* values (adjusted *p*‐values) as described by earlier studies (Benjamini & Hochberg, 1995).

#### 5.4.4 Candidate gene analysis underlying QTL region

The traits and SSR markers associated were aligned to the IRGSP 1.0 genome on the Rice Annotation Project (https://rapdb.dna.affrc.go.jp/viewer/gbrowse/irgsp1/) to retrieve the genes associated with linked markers. The sequence on either side of each linked marker extended from 1 Mb left and right was marked as QTL region was meticulously searched to find the genes associated with resistance against sheath blight. The interval on either side of the associated marker can be called intervals of the QTL genome.

#### 5.4.5 Statistical analysis

Descriptive statistics, ANOVA, and heritability were performed using Windostat 7.5 software. Hierarchical clustering was carried out for 330 genotypes to identify genotypes having similar phenotypic expression against sheath blight disease using the “hclust” function, method = “Euclidean” in R. Similarly, PCA and biplot analysis were executed using the PCA function from the *FactoMine* R package (Lê et al., 2008). To understand the interaction between the morphological traits, disease score, and AUDPC, Pearson correlation was executed using the *corrplot* functions from the *corrplot* package (Wei & Simko, 2016) in R (version 3.6.3).

## AUTHOR CONTRIBUTIONS

The idea was conceived and executed by R Naveenkumar, Annamalai Anandan, Anumalla Mahender, and Jauhar Ali. The experimental data collection, phenotypic trait characterization, data analysis, and manuscript writing were done by R Naveenkumar, Annamalai Anandan, S. R. Prabhukarthikeyan, and Ganesan Sangeetha. Genotypes were shared by Annamalai Anandan and P. K. Singh. The manuscript was improved and suggestions were made by R Naveenkumar, Annamalai Anandan, Anumalla Mahender, Shyam Saran Vaish, Waseem Hussain, and Jauhar Ali. All authors read and approved the final manuscript.

## CONFLICT OF INTEREST STATEMENT

The authors declare no conflict of interest.

## PEER REVIEW

The peer review history for this article is available in the Supporting Information for this article.

## Supporting information


**Data S1.** Peer Review

**Figure S1.** Biplot graph represents genotypes in two main principal components for traits associated with sheath blight disease in dry season 2019.

**Figure S2.** Biplot graph represents genotypes in two main principal components for traits associated with sheath blight disease in wet season 2019.

**Figure S3.** Correlation among the traits associated with sheath blight disease in dry season 2019.

**Figure S4.** Correlation among the traits associated with sheath blight disease in dry season 2019.

**Figure S5.** Network analysis of co‐expressed genes in the QTLs interval regions.

**Table S1.** List of rice accession used for preliminary screening against sheath blight disease reaction.
**Table S2.** List of SSR primers used in the present study to understand genetic diversity, structure and association mapping to identify the loci for resistance against sheath blight disease in rice.
**Table S3.** Descriptive statistics of different traits of core panel population over the season.
**Table S4.** Descriptive statistics of different traits of panel population during dry season 2019.
**Table S5.** Descriptive statistics of different traits of panel population during wet season 2019.
**Table S6.** Heritability and genetic advance for morphological and sheath blight related traits of 192 rice germplasm on dry and wet seasons.
**Table S7.** Details of SSR primers used for genotyping the core panel population consisting 192 genotypes and their estimated molecular genetic diversity parameters.
**Table S8.** Population structure group of rice genotypes based on inferred ancestry values.
**Table S9.** Association of marker alleles with disease‐related traits of sheath blight and other morphological traits in rice detected both in GLM and MLM (Q + K) analyses in a shortlisted panel population of 192 genotypes.
**Table S10.** Marker loci highly significant association with sheath blight resistance over season.
**Table S11.** Favorable and non‐favorable allelic variation of 30 QTLs regulating sheath blight resistance in the panel population.
**Table S12.** Favorable and non‐favorable allelic variation of 30 QTLs regulating sheath blight resistance in the panel population.

## Data Availability

The phenotypic and genotypic datasets analyzed during the current study are available from the corresponding author upon request.
