# Supplementary figures and images for: Dissecting genomic regions and underlying sheath blight resistance traits in rice ( Oryza sativa L.) using a genome‐wide association study
Source: Plant Direct. 2023 Nov 23;7(11):e540. doi: 10.1002/pld3.540 (PMC10667636; doi:10.1002/pld3.540)

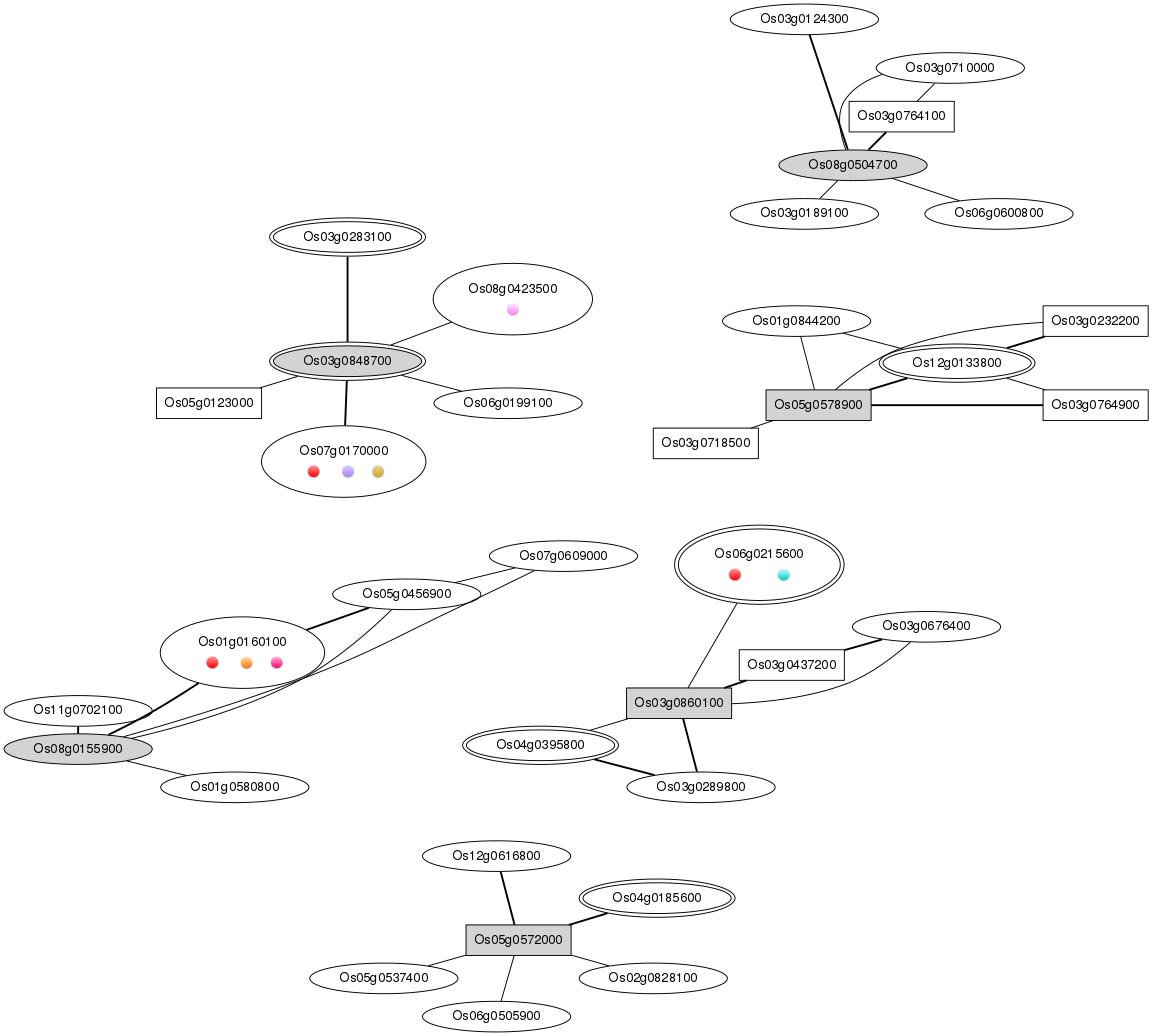

Supplement: Supplementary file 6 — Figure S5. Network analysis of co‐expressed genes in the QTLs interval regions. [file PLD3-7-e540-s005.jpg]
