# Supplementary material for: Dissecting genomic regions and underlying sheath blight resistance traits in rice ( Oryza sativa L.) using a genome‐wide association study
Source: Plant Direct. 2023 Nov 23;7(11):e540. doi: 10.1002/pld3.540 (PMC10667636; doi:10.1002/pld3.540)
Supplement: Supplementary file 7 — Table S1. List of rice accession used for preliminary screening against sheath blight disease reaction. Table S2. List of SSR primers used in the present study to understand genetic diversity, structure and association mapping to identify the loci for resistance against sheath blight disease in rice. Table S3. Descriptive statistics of different traits of core panel population over the season. Table S4. Descriptive statistics of different traits of panel population during dry season 2019. Table S5. Descriptive statistics of different traits of panel population during wet season 2019. Table S6. Heritability and genetic advance for morphological and sheath blight related traits of 192 rice germplasm on dry and wet seasons. Table S7. Details of SSR primers used for genotyping the core panel population consisting 192 genotypes and their estimated molecular genetic diversity parameters. Table S8. Population structure group of rice genotypes based on inferred ancestry values. Table S9. Association of marker alleles with disease‐related traits of sheath blight and other morphological traits in rice detected both in GLM and MLM (Q + K) analyses in a shortlisted panel population of 192 genotypes. Table S10. Marker loci highly significant association with sheath blight resistance over season. Table S11. Favorable and non‐favorable allelic variation of 30 QTLs regulating sheath blight resistance in the panel population. Table S12. Favorable and non‐favorable allelic variation of 30 QTLs regulating sheath blight resistance in the panel population. [file PLD3-7-e540-s004.docx]

**Supplementary Table 1**. List of rice accession used for preliminary screening against sheath blight disease reaction.

| **Sl. No.** | **Name of the varieties** | **Source of Collection** | **Plant Height** | **No. of Tillers/Plant** | **Panicle Length** | **Days to 50% flowering** | **Mean PDI** |
| --- | --- | --- | --- | --- | --- | --- | --- |
| 1 | Azucena | NRRI, Cuttack, Odisha | 99.52 | 2.00 | 23.50 | 92 | 9.26 |
| 2 | Domsufod | NRRI, Cuttack, Odisha | 110.08 | 8.33 | 23.13 | 83 | 11.11 |
| 3 | Dular | NRRI, Cuttack, Odisha | 77.25 | 10.33 | 21.37 | 86 | 15.74 |
| 4 | IR-64-21 | NRRI, Cuttack, Odisha | 98.75 | 6.17 | 13.73 | 81 | 12.96 |
| 5 | N 22 | NRRI, Cuttack, Odisha | 121.42 | 4.83 | 24.75 | 78 | 14.81 |
| 6 | Pokkali | NRRI, Cuttack, Odisha | 164.23 | 5.17 | 25.33 | 97 | 11.11 |
| 7 | Sadu cha | NRRI, Cuttack, Odisha | 116.63 | 4.83 | 19.03 | 81 | 12.04 |
| 8 | Sanhuangzhan | NRRI, Cuttack, Odisha | 87.33 | 12.17 | 22.33 | 89 | 19.91 |
| 9 | Zhenshan 97 b | NRRI, Cuttack, Odisha | 74.80 | 7.50 | 20.03 | 87 | 21.76 |
| 10 | Cypress | NRRI, Cuttack, Odisha | 78.87 | 2.17 | 18.45 | 86 | 24.07 |
| 11 | ARC 6578 | NRRI, Cuttack, Odisha | 109.12 | 4.00 | 22.42 | 89 | 18.52 |
| 12 | ARC 7229 | NRRI, Cuttack, Odisha | 114.88 | 3.50 | 23.70 | 95 | 11.57 |
| 13 | Kasalath | NRRI, Cuttack, Odisha | 108.13 | 8.17 | 21.58 | 89 | 12.50 |
| 14 | ARC 10376 | NRRI, Cuttack, Odisha | 94.22 | 6.67 | 17.78 | 85 | 12.50 |
| 15 | Black Gora | NRRI, Cuttack, Odisha | 92.87 | 6.67 | 44.88 | 89 | 15.28 |
| 16 | CTG 1516 | NRRI, Cuttack, Odisha | 95.25 | 6.67 | 19.85 | 87 | 12.96 |
| 17 | DD 62 | NRRI, Cuttack, Odisha | 98.75 | 4.00 | 19.75 | 90 | 20.37 |
| 18 | Dhala Shaiptta | NRRI, Cuttack, Odisha | 86.55 | 3.17 | 22.37 | 83 | 25.00 |
| 19 | DJ 24 | NRRI, Cuttack, Odisha | 102.80 | 4.33 | 21.02 | 96 | 14.81 |
| 20 | BM 43 | NRRI, Cuttack, Odisha | 99.20 | 11.50 | 19.97 | 81 | 12.04 |
| 21 | BM 56 | NRRI, Cuttack, Odisha | 102.60 | 3.67 | 17.72 | 87 | 13.89 |
| 22 | DM 59 | NRRI, Cuttack, Odisha | 101.47 | 7.17 | 20.58 | 89 | 11.11 |
| 23 | DV 123 | NRRI, Cuttack, Odisha | 103.63 | 5.50 | 16.88 | 87 | 14.35 |
| 24 | DV 85 | NRRI, Cuttack, Odisha | 98.32 | 4.00 | 18.72 | 85 | 11.11 |
| 25 | Ghor Bhai | NRRI, Cuttack, Odisha | 85.88 | 6.83 | 17.23 | 89 | 13.89 |
| 26 | Goria | NRRI, Cuttack, Odisha | 100.18 | 6.00 | 20.32 | 79 | 10.19 |
| 27 | Jamir | NRRI, Cuttack, Odisha | 100.03 | 9.83 | 43.00 | 78 | 16.67 |
| 28 | Jhona 349 | NRRI, Cuttack, Odisha | 91.52 | 5.83 | 15.57 | 88 | 14.81 |
| 29 | Kachilon | NRRI, Cuttack, Odisha | 101.07 | 8.83 | 23.73 | 81 | 10.19 |
| 30 | Kalamkati | NRRI, Cuttack, Odisha | 97.92 | 5.67 | 19.38 | 92 | 17.59 |
| 31 | Karkati 87 | NRRI, Cuttack, Odisha | 87.05 | 8.50 | 16.13 | 89 | 15.74 |
| 32 | PTB 30 | NRRI, Cuttack, Odisha | 103.83 | 10.33 | 20.64 | 89 | 11.11 |
| 33 | Surjamukhi | NRRI, Cuttack, Odisha | 93.63 | 5.33 | 19.95 | 89 | 20.83 |
| 34 | T1 | NRRI, Cuttack, Odisha | 112.17 | 6.83 | 19.50 | 92 | 12.50 |
| 35 | T-26 | NRRI, Cuttack, Odisha | 108.60 | 7.00 | 26.47 | 98 | 9.26 |
| 36 | 99216 | NRRI, Cuttack, Odisha | 97.97 | 7.67 | 21.23 | 83 | 13.89 |
| 37 | ARC 10378 | NRRI, Cuttack, Odisha | 106.98 | 7.67 | 24.32 | 83 | 12.96 |
| 38 | Bowalia | NRRI, Cuttack, Odisha | 99.18 | 7.17 | 18.95 | 89 | 13.89 |
| 39 | NP 97 | NRRI, Cuttack, Odisha | 286.00 | 5.50 | 26.00 | 92 | 7.41 |
| 40 | Asahi | NRRI, Cuttack, Odisha | 71.38 | 8.67 | 20.67 | 89 | 16.67 |
| 41 | Bachi Boro | NRRI, Cuttack, Odisha | 121.05 | 9.00 | 21.80 | 85 | 12.50 |
| 42 | BR6 | NRRI, Cuttack, Odisha | 73.28 | 13.17 | 21.78 | 86 | 16.20 |
| 43 | Chhola Boro | NRRI, Cuttack, Odisha | 103.05 | 16.50 | 17.13 | 92 | 12.04 |
| 44 | Lara | NRRI, Cuttack, Odisha | 114.80 | 10.67 | 14.97 | 85 | 10.65 |
| 45 | Rata Boro | NRRI, Cuttack, Odisha | 108.40 | 9.83 | 19.37 | 83 | 12.04 |
| 46 | Tulsi Boro | NRRI, Cuttack, Odisha | 120.13 | 11.67 | 19.02 | 92 | 8.80 |
| 47 | Tupa | NRRI, Cuttack, Odisha | 100.82 | 9.33 | 45.35 | 86 | 12.04 |
| 48 | Gobir Sail | NRRI, Cuttack, Odisha | 91.78 | 4.67 | 16.72 | 88 | 19.44 |
| 49 | Lahaya | NRRI, Cuttack, Odisha | 95.82 | 9.00 | 17.00 | 87 | 12.96 |
| 50 | Anjani | NRRI, Cuttack, Odisha | 88.47 | 11.33 | 16.93 | 87 | 21.30 |
| 51 | ARC 11205 | NRRI, Cuttack, Odisha | 99.92 | 10.00 | 18.12 | 94 | 14.81 |
| 52 | ARC 11600 | NRRI, Cuttack, Odisha | 96.62 | 5.67 | 18.23 | 87 | 16.67 |
| 53 | ARC 14855 | NRRI, Cuttack, Odisha | 103.38 | 16.67 | 19.45 | 89 | 6.48 |
| 54 | ARC 14950 | NRRI, Cuttack, Odisha | 97.02 | 8.33 | 19.83 | 94 | 13.43 |
| 55 | ARC 14965 | NRRI, Cuttack, Odisha | 85.77 | 12.17 | 18.07 | 82 | 8.80 |
| 56 | ARC 14969 | NRRI, Cuttack, Odisha | 101.62 | 9.00 | 18.52 | 82 | 11.11 |
| 57 | ARC 5959 | NRRI, Cuttack, Odisha | 88.57 | 5.83 | 19.62 | 92 | 16.67 |
| 58 | ARC 5960 | NRRI, Cuttack, Odisha | 107.10 | 8.33 | 20.97 | 94 | 11.11 |
| 59 | ARC 5977 | NRRI, Cuttack, Odisha | 109.23 | 7.83 | 22.65 | 90 | 11.11 |
| 60 | ARC 7098 | NRRI, Cuttack, Odisha | 99.72 | 9.33 | 20.90 | 86 | 7.87 |
| 61 | ARC 7325 | NRRI, Cuttack, Odisha | 104.70 | 9.67 | 19.30 | 85 | 12.50 |
| 62 | AS 2 | NRRI, Cuttack, Odisha | 111.58 | 18.00 | 16.77 | 92 | 14.35 |
| 63 | AUS 100 | NRRI, Cuttack, Odisha | 97.62 | 5.33 | 20.32 | 87 | 10.19 |
| 64 | AUS 125 | NRRI, Cuttack, Odisha | 88.40 | 11.67 | 16.68 | 89 | 18.52 |
| 65 | AUS 12 | NRRI, Cuttack, Odisha | 102.43 | 12.00 | 21.60 | 94 | 6.02 |
| 66 | AUS 130 | NRRI, Cuttack, Odisha | 104.13 | 4.00 | 21.18 | 99 | 12.50 |
| 67 | AUS 131 | NRRI, Cuttack, Odisha | 107.52 | 6.17 | 19.87 | 85 | 10.19 |
| 68 | AUS 151 | NRRI, Cuttack, Odisha | 105.05 | 6.33 | 20.93 | 88 | 9.26 |
| 69 | AUS 169 | NRRI, Cuttack, Odisha | 108.97 | 8.50 | 22.22 | 81 | 10.65 |
| 70 | AUS 175 | NRRI, Cuttack, Odisha | 108.08 | 8.67 | 23.98 | 81 | 9.26 |
| 71 | AUS 180 | NRRI, Cuttack, Odisha | 108.13 | 7.50 | 22.58 | 81 | 10.65 |
| 72 | AUS 204 | NRRI, Cuttack, Odisha | 108.97 | 7.83 | 22.13 | 81 | 12.04 |
| 73 | AUS 209 | NRRI, Cuttack, Odisha | 102.38 | 7.83 | 19.50 | 81 | 12.04 |
| 74 | AUS 210 | NRRI, Cuttack, Odisha | 92.15 | 6.17 | 17.07 | 87 | 15.28 |
| 75 | AUS 228 | NRRI, Cuttack, Odisha | 91.13 | 9.33 | 18.35 | 92 | 13.43 |
| 76 | AUS 267 | NRRI, Cuttack, Odisha | 108.23 | 9.17 | 23.73 | 85 | 9.26 |
| 77 | AUS 268 | NRRI, Cuttack, Odisha | 100.47 | 8.67 | 20.93 | 88 | 13.89 |
| 78 | AUS 273 | NRRI, Cuttack, Odisha | 276.60 | 6.50 | 17.78 | 89 | 9.26 |
| 79 | Aus 277 | NRRI, Cuttack, Odisha | 113.55 | 5.83 | 20.25 | 94 | 10.19 |
| 80 | AUS 283 | NRRI, Cuttack, Odisha | 95.92 | 6.83 | 22.45 | 88 | 10.65 |
| 81 | AUS 293 | NRRI, Cuttack, Odisha | 88.57 | 5.33 | 18.85 | 88 | 15.74 |
| 82 | AUS 28 | NRRI, Cuttack, Odisha | 109.35 | 9.00 | 21.58 | 94 | 8.80 |
| 83 | AUS 314 | NRRI, Cuttack, Odisha | 95.13 | 5.17 | 22.47 | 89 | 7.87 |
| 84 | AUS 31 | NRRI, Cuttack, Odisha | 111.47 | 5.67 | 21.18 | 94 | 11.11 |
| 85 | Aus 335 | NRRI, Cuttack, Odisha | 130.53 | 4.33 | 23.68 | 89 | 11.11 |
| 86 | AUS 350 | NRRI, Cuttack, Odisha | 89.27 | 6.83 | 18.48 | 87 | 13.89 |
| 87 | AUS 362 | NRRI, Cuttack, Odisha | 86.77 | 7.17 | 16.63 | 94 | 8.33 |
| 88 | AUS 354 | NRRI, Cuttack, Odisha | 86.47 | 8.67 | 16.67 | 89 | 7.87 |
| 89 | AUS 369 | NRRI, Cuttack, Odisha | 86.27 | 5.17 | 18.73 | 92 | 14.35 |
| 90 | AUS 382 | NRRI, Cuttack, Odisha | 92.77 | 8.67 | 20.95 | 90 | 12.04 |
| 91 | AUS 411 | NRRI, Cuttack, Odisha | 90.27 | 7.67 | 19.05 | 86 | 11.11 |
| 92 | AUS 385 | NRRI, Cuttack, Odisha | 88.78 | 8.33 | 21.90 | 92 | 14.35 |
| 93 | AUS 414 | NRRI, Cuttack, Odisha | 80.83 | 7.83 | 19.35 | 94 | 22.22 |
| 94 | AUS 415 | NRRI, Cuttack, Odisha | 84.97 | 5.50 | 20.50 | 94 | 16.67 |
| 95 | AUS 417 | NRRI, Cuttack, Odisha | 92.27 | 6.17 | 18.63 | 94 | 11.11 |
| 96 | AUS 420 | NRRI, Cuttack, Odisha | 103.73 | 5.50 | 23.58 | 94 | 15.74 |
| 97 | Aus 435 | NRRI, Cuttack, Odisha | 89.53 | 6.33 | 21.72 | 94 | 10.65 |
| 98 | AUS 440 | NRRI, Cuttack, Odisha | 87.28 | 6.00 | 23.12 | 94 | 14.35 |
| 99 | AUS 455 | NRRI, Cuttack, Odisha | 107.87 | 6.17 | 24.72 | 94 | 12.04 |
| 100 | AUS 462 | NRRI, Cuttack, Odisha | 97.38 | 8.00 | 21.97 | 94 | 18.06 |
| 101 | AUS 464 | NRRI, Cuttack, Odisha | 101.73 | 8.33 | 21.07 | 83 | 9.72 |
| 102 | AUS 46 | NRRI, Cuttack, Odisha | 104.28 | 11.50 | 18.87 | 89 | 9.72 |
| 103 | AUS 60 | NRRI, Cuttack, Odisha | 85.23 | 10.67 | 18.68 | 86 | 15.28 |
| 104 | Aus 62 | NRRI, Cuttack, Odisha | 92.35 | 12.67 | 17.63 | 91 | 18.52 |
| 105 | AUS 74 | NRRI, Cuttack, Odisha | 93.35 | 14.50 | 20.43 | 81 | 9.72 |
| 106 | AUS 77 | NRRI, Cuttack, Odisha | 90.75 | 10.50 | 17.55 | 90 | 27.31 |
| 107 | AUS khusi | NRRI, Cuttack, Odisha | 88.07 | 8.67 | 20.07 | 85 | 15.74 |
| 108 | AUSMERI | NRRI, Cuttack, Odisha | 108.93 | 7.17 | 22.17 | 81 | 10.65 |
| 109 | AUS murali | NRRI, Cuttack, Odisha | 98.67 | 13.33 | 21.10 | 83 | 13.43 |
| 110 | AUS paddy(black) | NRRI, Cuttack, Odisha | 86.60 | 12.83 | 17.83 | 86 | 13.89 |
| 111 | Bausphr | NRRI, Cuttack, Odisha | 120.82 | 7.33 | 20.38 | 90 | 15.28 |
| 112 | Bcularn | NRRI, Cuttack, Odisha | 103.30 | 13.33 | 20.30 | 82 | 9.26 |
| 113 | Bircona | NRRI, Cuttack, Odisha | 107.60 | 6.17 | 19.72 | 82 | 10.65 |
| 114 | Boro black | NRRI, Cuttack, Odisha | 87.17 | 9.00 | 13.28 | 90 | 15.28 |
| 115 | Boro | NRRI, Cuttack, Odisha | 105.92 | 9.50 | 18.97 | 86 | 12.04 |
| 116 | Bowalia2 | NRRI, Cuttack, Odisha | 94.40 | 5.83 | 21.35 | 85 | 12.50 |
| 117 | Bowalia | NRRI, Cuttack, Odisha | 105.58 | 7.33 | 21.62 | 83 | 8.33 |
| 118 | Brown gora s b 92 | NRRI, Cuttack, Odisha | 85.05 | 7.00 | 17.42 | 85 | 8.33 |
| 119 | Chamka | NRRI, Cuttack, Odisha | 105.67 | 8.83 | 24.27 | 80 | 7.41 |
| 120 | Chandra kana | NRRI, Cuttack, Odisha | 101.43 | 13.00 | 16.18 | 85 | 11.57 |
| 121 | Chandra mukhi | NRRI, Cuttack, Odisha | 109.52 | 8.17 | 17.77 | 87 | 12.50 |
| 122 | Chinger | NRRI, Cuttack, Odisha | 110.20 | 16.83 | 17.87 | 92 | 10.19 |
| 123 | CN 2- 175-5-31 | NRRI, Cuttack, Odisha | 83.65 | 10.67 | 16.43 | 94 | 16.67 |
| 124 | BI 93 | NRRI, Cuttack, Odisha | 117.17 | 7.00 | 18.52 | 108 | 11.11 |
| 125 | CTG 250 | NRRI, Cuttack, Odisha | 96.73 | 8.17 | 19.92 | 93 | 15.28 |
| 126 | Cunail | NRRI, Cuttack, Odisha | 96.52 | 5.67 | 18.95 | 94 | 13.43 |
| 127 | DA 12 | NRRI, Cuttack, Odisha | 94.52 | 11.33 | 17.90 | 92 | 4.63 |
| 128 | DA 2 | NRRI, Cuttack, Odisha | 76.65 | 6.33 | 13.55 | 90 | 9.26 |
| 129 | DF 3 | NRRI, Cuttack, Odisha | 90.65 | 6.33 | 18.12 | 88 | 13.89 |
| 130 | Dhalai Kachri | NRRI, Cuttack, Odisha | 107.85 | 9.17 | 21.92 | 85 | 9.26 |
| 131 | Dhaliboro 111-3 | NRRI, Cuttack, Odisha | 95.33 | 7.17 | 16.48 | 90 | 16.67 |
| 132 | DJ 29 | NRRI, Cuttack, Odisha | 89.22 | 7.33 | 15.62 | 94 | 16.20 |
| 133 | Dubhi Gora | NRRI, Cuttack, Odisha | 113.17 | 13.67 | 22.22 | 100 | 12.04 |
| 134 | Dulaaus | NRRI, Cuttack, Odisha | 93.23 | 10.50 | 16.53 | 104 | 7.87 |
| 135 | Early Sutarsar 39 | NRRI, Cuttack, Odisha | 109.78 | 6.17 | 22.63 | 96 | 11.11 |
| 136 | Gohama Bhadri | NRRI, Cuttack, Odisha | 107.35 | 10.17 | 17.60 | 91 | 8.80 |
| 137 | Hijli | NRRI, Cuttack, Odisha | 100.72 | 5.67 | 21.87 | 98 | 12.96 |
| 138 | Jagle Boro | NRRI, Cuttack, Odisha | 90.65 | 8.33 | 19.90 | 86 | 12.50 |
| 139 | Kada 176-12 | NRRI, Cuttack, Odisha | 90.98 | 8.67 | 17.12 | 86 | 13.89 |
| 140 | Kalasu | NRRI, Cuttack, Odisha | 86.03 | 8.67 | 18.82 | 83 | 11.11 |
| 141 | Kal Buri | NRRI, Cuttack, Odisha | 116.98 | 7.67 | 22.20 | 85 | 8.33 |
| 142 | Kali Aus | NRRI, Cuttack, Odisha | 103.75 | 8.50 | 16.70 | 87 | 14.81 |
| 143 | Kaliboro138-2 | NRRI, Cuttack, Odisha | 95.65 | 11.17 | 18.75 | 87 | 11.57 |
| 144 | Kaliboro 80-3 | NRRI, Cuttack, Odisha | 111.17 | 5.67 | 20.85 | 93 | 15.28 |
| 145 | Kalindi | NRRI, Cuttack, Odisha | 92.98 | 6.00 | 24.20 | 95 | 13.43 |
| 146 | M 136-20 | NRRI, Cuttack, Odisha | 95.18 | 9.00 | 18.98 | 92 | 19.44 |
| 147 | Padma Sail | NRRI, Cuttack, Odisha | 103.12 | 14.00 | 21.88 | 96 | 10.19 |
| 148 | Parbat Jira | NRRI, Cuttack, Odisha | 104.12 | 11.00 | 22.32 | 91 | 26.39 |
| 149 | Sorishaful | NRRI, Cuttack, Odisha | 102.38 | 5.50 | 23.13 | 88 | 9.72 |
| 150 | Tepa Boro 508 | NRRI, Cuttack, Odisha | 117.92 | 12.33 | 24.13 | 83 | 9.26 |
| 151 | White Dubhi | NRRI, Cuttack, Odisha | 115.88 | 9.83 | 21.22 | 87 | 11.57 |
| 152 | AUS 152 | NRRI, Cuttack, Odisha | 104.93 | 6.67 | 20.62 | 86 | 14.81 |
| 153 | AUS 154 | NRRI, Cuttack, Odisha | 90.83 | 8.17 | 19.77 | 81 | 17.59 |
| 154 | AUS 321 | NRRI, Cuttack, Odisha | 87.70 | 8.17 | 18.88 | 91 | 13.89 |
| 155 | AUS 366 | NRRI, Cuttack, Odisha | 106.72 | 5.83 | 20.73 | 93 | 12.50 |
| 156 | AUS 391 | NRRI, Cuttack, Odisha | 105.40 | 7.00 | 16.47 | 85 | 10.19 |
| 157 | AUS 63 | NRRI, Cuttack, Odisha | 108.87 | 6.33 | 20.88 | 81 | 11.57 |
| 158 | Dhingha | NRRI, Cuttack, Odisha | 105.13 | 5.33 | 19.05 | 83 | 10.19 |
| 159 | Kali Boro 2-2 | NRRI, Cuttack, Odisha | 86.98 | 5.50 | 16.00 | 91 | 18.06 |
| 160 | kaliboro 26 | NRRI, Cuttack, Odisha | 94.15 | 8.00 | 19.90 | 88 | 13.89 |
| 161 | MTU 18 | NRRI, Cuttack, Odisha | 110.80 | 7.83 | 22.85 | 91 | 12.04 |
| 162 | Nai Dumra | NRRI, Cuttack, Odisha | 113.97 | 4.33 | 23.70 | 93 | 10.19 |
| 163 | Raj Munlo | NRRI, Cuttack, Odisha | 91.92 | 4.00 | 20.55 | 89 | 11.11 |
| 164 | Soa Mukhi | NRRI, Cuttack, Odisha | 96.15 | 6.00 | 19.55 | 83 | 12.50 |
| 165 | Nagri | NRRI, Cuttack, Odisha | 115.00 | 5.83 | 20.73 | 94 | 13.89 |
| 166 | Boraya | NRRI, Cuttack, Odisha | 111.50 | 5.17 | 25.35 | 90 | 11.57 |
| 167 | Choudhury Sail | NRRI, Cuttack, Odisha | 103.15 | 7.83 | 20.42 | 87 | 12.50 |
| 168 | AUS 16 | NRRI, Cuttack, Odisha | 108.40 | 4.33 | 21.12 | 86 | 12.96 |
| 169 | AUS 298 | NRRI, Cuttack, Odisha | 91.07 | 10.17 | 20.75 | 130 | 12.04 |
| 170 | AUS 364 | NRRI, Cuttack, Odisha | 105.88 | 9.33 | 24.18 | 122 | 8.80 |
| 171 | CO-43 | TNAU, Coimbatore, Tamil Nadu | 74.17 | 12.67 | 18.55 | 104 | 16.20 |
| 172 | CO-47 | TNAU, Coimbatore, Tamil Nadu | 80.72 | 7.67 | 22.02 | 122 | 8.80 |
| 173 | CO(R)-48 | TNAU, Coimbatore, Tamil Nadu | 103.50 | 9.00 | 27.58 | 126 | 11.11 |
| 174 | CO(R)-49 | TNAU, Coimbatore, Tamil Nadu | 84.82 | 7.83 | 20.90 | 95 | 14.81 |
| 175 | CO(R)-51 | TNAU, Coimbatore, Tamil Nadu | 83.65 | 7.17 | 22.87 | 94 | 19.91 |
| 176 | CO(R)-52 | TNAU, Coimbatore, Tamil Nadu | 90.88 | 5.83 | 22.50 | 104 | 14.81 |
| 177 | ASD-16 | TNAU, Coimbatore, Tamil Nadu | 90.88 | 11.33 | 20.27 | 132 | 9.26 |
| 178 | TPS-5 | TNAU, Coimbatore, Tamil Nadu | 82.55 | 10.00 | 23.93 | 103 | 16.67 |
| 179 | GEB-24 | TNAU, Coimbatore, Tamil Nadu | 97.32 | 11.67 | 18.32 | 123 | 9.26 |
| 180 | ANNA (r)-4 | TNAU, Coimbatore, Tamil Nadu | 87.02 | 8.67 | 17.82 | 126 | 7.87 |
| 181 | Improved white ponni | TNAU, Coimbatore, Tamil Nadu | 82.87 | 10.33 | 17.67 | 140 | 4.91 |
| 182 | BPT 5204 | TNAU, Coimbatore, Tamil Nadu | 81.53 | 8.67 | 17.07 | 126 | 8.33 |
| 183 | BPT 5204 sub 1 | TNAU, Coimbatore, Tamil Nadu | 75.70 | 7.50 | 21.20 | 128 | 7.99 |
| 184 | CR 1009 sub 1 | TNAU, Coimbatore, Tamil Nadu | 118.60 | 9.00 | 23.07 | 130 | 7.41 |
| 185 | IR-20 | TNAU, Coimbatore, Tamil Nadu | 86.45 | 10.50 | 23.12 | 116 | 14.81 |
| 186 | IR-36 | TNAU, Coimbatore, Tamil Nadu | 112.15 | 10.17 | 22.38 | 110 | 11.57 |
| 187 | IR-64 | TNAU, Coimbatore, Tamil Nadu | 86.45 | 10.50 | 23.12 | 116 | 14.81 |
| 188 | Rasakadam | TNAU, Coimbatore, Tamil Nadu | 138.50 | 5.33 | 27.37 | 114 | 11.57 |
| 189 | Sornavari | TNAU, Coimbatore, Tamil Nadu | 105.05 | 5.83 | 18.77 | 143 | 11.11 |
| 190 | Kodaikulathan | TNAU, Coimbatore, Tamil Nadu | 126.03 | 5.83 | 56.77 | 128 | 10.65 |
| 191 | Vadakathai samba | TNAU, Coimbatore, Tamil Nadu | 118.27 | 5.67 | 20.73 | 140 | 12.04 |
| 192 | Panamara samba | TNAU, Coimbatore, Tamil Nadu | 106.00 | 5.33 | 20.88 | 140 | 8.80 |
| 193 | Nootri Pathu | TNAU, Coimbatore, Tamil Nadu | 100.73 | 7.33 | 22.35 | 97 | 11.11 |
| 194 | Murugan Kar | TNAU, Coimbatore, Tamil Nadu | 111.23 | 8.17 | 18.80 | 130 | 11.11 |
| 195 | Mattai Kar | TNAU, Coimbatore, Tamil Nadu | 114.58 | 6.83 | 22.68 | 135 | 10.65 |
| 196 | Ramakuruvai Kar | TNAU, Coimbatore, Tamil Nadu | 127.40 | 11.17 | 25.32 | 175 | 9.72 |
| 197 | Katta Samba | TNAU, Coimbatore, Tamil Nadu | 116.72 | 9.33 | 24.37 | 147 | 10.19 |
| 198 | Mappillai Samba | TNAU, Coimbatore, Tamil Nadu | 118.60 | 10.00 | 22.72 | 137 | 10.65 |
| 199 | Kauni | TNAU, Coimbatore, Tamil Nadu | 115.13 | 8.50 | 20.95 | 125 | 8.80 |
| 200 | Norungan | TNAU, Coimbatore, Tamil Nadu | 98.38 | 15.67 | 20.25 | 126 | 12.96 |
| 201 | Sivappu Chithirai Kar | TRRI, Aduthurai, Tamil Nadu | 110.65 | 17.00 | 23.10 | 103 | 10.65 |
| 202 | Swarna Masuri | TRRI, Aduthurai, Tamil Nadu | 85.82 | 10.17 | 20.90 | 122 | 10.65 |
| 203 | Soora Kuruvai | TRRI, Aduthurai, Tamil Nadu | 89.33 | 8.83 | 21.28 | 122 | 8.33 |
| 204 | Adu Kar | TRRI, Aduthurai, Tamil Nadu | 76.95 | 7.67 | 18.70 | 126 | 11.57 |
| 205 | Jaya | TRRI, Aduthurai, Tamil Nadu | 125.32 | 6.67 | 24.45 | 119 | 10.19 |
| 206 | Mtu-1001 | TRRI, Aduthurai, Tamil Nadu | 126.15 | 7.33 | 23.65 | 131 | 9.26 |
| 207 | Kalanamak | TRRI, Aduthurai, Tamil Nadu | 78.50 | 11.17 | 21.15 | 128 | 23.61 |
| 208 | Kayama | TRRI, Aduthurai, Tamil Nadu | 113.45 | 6.67 | 17.42 | 135 | 13.43 |
| 209 | Swarna | TRRI, Aduthurai, Tamil Nadu | 71.98 | 11.50 | 18.17 | 102 | 14.81 |
| 210 | Thooya malli | TRRI, Aduthurai, Tamil Nadu | 80.37 | 7.33 | 19.50 | 100 | 10.65 |
| 211 | ADT-36 | TRRI, Aduthurai, Tamil Nadu | 82.28 | 11.00 | 22.93 | 119 | 5.09 |
| 212 | ADT-37 | TRRI, Aduthurai, Tamil Nadu | 73.02 | 14.00 | 19.75 | 124 | 16.20 |
| 213 | ADT-38 | TRRI, Aduthurai, Tamil Nadu | 81.43 | 6.50 | 22.17 | 138 | 11.11 |
| 214 | ADT-39 | TRRI, Aduthurai, Tamil Nadu | 73.93 | 11.33 | 21.52 | 97 | 21.30 |
| 215 | ADT-40 | TRRI, Aduthurai, Tamil Nadu | 81.33 | 10.33 | 23.97 | 119 | 25.00 |
| 216 | ADT-41 | TRRI, Aduthurai, Tamil Nadu | 72.47 | 13.83 | 21.58 | 103 | 25.00 |
| 217 | ADT-42 | TRRI, Aduthurai, Tamil Nadu | 88.02 | 8.50 | 20.27 | 131 | 8.33 |
| 218 | ADT-43 | TRRI, Aduthurai, Tamil Nadu | 71.23 | 14.67 | 16.13 | 95 | 36.11 |
| 219 | ADT-44 | TRRI, Aduthurai, Tamil Nadu | 81.58 | 9.67 | 22.60 | 140 | 12.50 |
| 220 | ADT-45 | TRRI, Aduthurai, Tamil Nadu | 87.97 | 13.00 | 23.17 | 121 | 20.83 |
| 221 | ADT-46 | TRRI, Aduthurai, Tamil Nadu | 76.93 | 11.33 | 19.92 | 92 | 26.85 |
| 222 | ADT-47 | TRRI, Aduthurai, Tamil Nadu | 197.68 | 12.17 | 20.85 | 122 | 18.06 |
| 223 | ADT-48 | TRRI, Aduthurai, Tamil Nadu | 96.10 | 15.67 | 27.26 | 146 | 12.96 |
| 224 | ADT-49 | TRRI, Aduthurai, Tamil Nadu | 82.82 | 9.67 | 24.19 | 148 | 6.48 |
| 225 | ADT-50 | TRRI, Aduthurai, Tamil Nadu | 90.53 | 9.50 | 20.77 | 122 | 12.04 |
| 226 | ADT-51 | TRRI, Aduthurai, Tamil Nadu | 115.62 | 12.67 | 22.72 | 119 | 3.70 |
| 227 | TRY-2 | TRRI, Aduthurai, Tamil Nadu | 83.28 | 11.67 | 19.38 | 121 | 3.24 |
| 228 | TKM-12 | TRRI, Aduthurai, Tamil Nadu | 104.38 | 9.17 | 25.48 | 119 | 7.87 |
| 229 | TKM-13 | TRRI, Aduthurai, Tamil Nadu | 92.13 | 7.33 | 23.93 | 106 | 9.72 |
| 230 | CR Dhan 300 | NRRI, Cuttack, Odisha | 107.93 | 11.17 | 26.28 | 119 | 12.96 |
| 231 | CR Dhan 301 | NRRI, Cuttack, Odisha | 95.28 | 9.33 | 24.82 | 121 | 9.26 |
| 232 | CR Dhan 303 | NRRI, Cuttack, Odisha | 102.68 | 10.17 | 23.48 | 119 | 9.72 |
| 233 | CR Dhan 304 | NRRI, Cuttack, Odisha | 91.28 | 8.33 | 21.13 | 118 | 11.11 |
| 234 | CR Dhan 305 | NRRI, Cuttack, Odisha | 107.73 | 4.67 | 24.87 | 119 | 11.57 |
| 235 | CR Dhan 306 | NRRI, Cuttack, Odisha | 94.07 | 15.33 | 23.25 | 120 | 11.57 |
| 236 | Maudamani (CR Dhan 307) | NRRI, Cuttack, Odisha | 110.90 | 11.33 | 24.85 | 119 | 6.02 |
| 237 | CR Dhan 310 | NRRI, Cuttack, Odisha | 92.58 | 6.17 | 22.62 | 138 | 10.65 |
| 238 | CR Dhan 311 | NRRI, Cuttack, Odisha | 87.43 | 6.33 | 21.45 | 140 | 5.56 |
| 239 | Reeta (CR Dhan 401) | NRRI, Cuttack, Odisha | 86.05 | 9.50 | 23.33 | 104 | 11.11 |
| 240 | Sumit (CR Dhan 404) | NRRI, Cuttack, Odisha | 85.87 | 11.17 | 24.92 | 102 | 15.74 |
| 241 | Luna Sankhi (CR Dhan 405) | NRRI, Cuttack, Odisha | 102.63 | 6.83 | 20.38 | 135 | 8.80 |
| 242 | CR Dhan 408 | NRRI, Cuttack, Odisha | 111.25 | 7.50 | 18.75 | 140 | 5.09 |
| 243 | CR Dhan 500 | NRRI, Cuttack, Odisha | 106.78 | 6.33 | 20.68 | 137 | 9.72 |
| 244 | CR Dhan 501 | NRRI, Cuttack, Odisha | 100.65 | 9.67 | 22.17 | 138 | 11.11 |
| 245 | CR dhan 505 | NRRI, Cuttack, Odisha | 90.47 | 11.67 | 24.38 | 110 | 10.65 |
| 246 | CR Dhan 506 | NRRI, Cuttack, Odisha | 80.15 | 11.33 | 23.25 | 104 | 5.09 |
| 247 | CR Dhan 701 | NRRI, Cuttack, Odisha | 98.95 | 6.17 | 25.23 | 114 | 7.87 |
| 248 | CR Dhan 801 | NRRI, Cuttack, Odisha | 87.33 | 10.00 | 25.15 | 114 | 6.02 |
| 249 | Poorna Bhog (CR Dhan 902) | NRRI, Cuttack, Odisha | 82.52 | 13.83 | 17.98 | 125 | 12.50 |
| 250 | CR Dhan 907 | NRRI, Cuttack, Odisha | 84.95 | 10.67 | 20.37 | 127 | 14.35 |
| 251 | CR Dhan 908 | NRRI, Cuttack, Odisha | 121.95 | 6.33 | 22.05 | 140 | 10.19 |
| 252 | CR 1014 | NRRI, Cuttack, Odisha | 101.43 | 8.67 | 21.77 | 116 | 12.96 |
| 253 | Improved Lalat | NRRI, Cuttack, Odisha | 90.02 | 9.17 | 24.55 | 107 | 11.57 |
| 254 | Naveen | NRRI, Cuttack, Odisha | 101.92 | 9.17 | 19.85 | 98 | 11.57 |
| 255 | Vandana | NRRI, Cuttack, Odisha | 78.55 | 11.33 | 20.00 | 113 | 21.30 |
| 256 | HUR-105 | BHU, Varanasi, Uttar Pradesh | 87.72 | 4.67 | 23.27 | 115 | 10.65 |
| 257 | Sarjoo 52 | BHU, Varanasi, Uttar Pradesh | 90.57 | 7.00 | 23.92 | 113 | 12.50 |
| 258 | NDR-359 | BHU, Varanasi, Uttar Pradesh | 86.63 | 6.83 | 22.30 | 104 | 15.28 |
| 259 | Tulsi | BHU, Varanasi, Uttar Pradesh | 81.68 | 6.17 | 20.37 | 103 | 17.13 |
| 260 | Anjali | BHU, Varanasi, Uttar Pradesh | 90.05 | 7.33 | 24.25 | 117 | 8.80 |
| 261 | Sahbagi Dhan | BHU, Varanasi, Uttar Pradesh | 101.50 | 7.33 | 19.03 | 122 | 9.72 |
| 262 | Shusk Samrat | BHU, Varanasi, Uttar Pradesh | 74.02 | 9.17 | 21.63 | 115 | 17.59 |
| 263 | Barani deep | BHU, Varanasi, Uttar Pradesh | 75.87 | 9.33 | 18.95 | 126 | 6.94 |
| 264 | HUR-36 | BHU, Varanasi, Uttar Pradesh | 73.78 | 9.33 | 17.73 | 118 | 10.19 |
| 265 | HUR-43 | BHU, Varanasi, Uttar Pradesh | 83.10 | 5.17 | 14.95 | 100 | 8.33 |
| 266 | HUR-3022 | BHU, Varanasi, Uttar Pradesh | 82.57 | 12.33 | 14.83 | 99 | 6.48 |
| 267 | IC 277237 | BHU, Varanasi, Uttar Pradesh | 102.77 | 6.67 | 21.50 | 102 | 15.74 |
| 268 | IC 277319 | BHU, Varanasi, Uttar Pradesh | 106.45 | 6.50 | 24.40 | 104 | 8.80 |
| 269 | IC 278776 | BHU, Varanasi, Uttar Pradesh | 103.08 | 9.00 | 18.90 | 101 | 7.87 |
| 270 | IC 282454 | BHU, Varanasi, Uttar Pradesh | 55.43 | 10.33 | 16.17 | 98 | 27.31 |
| 271 | IC 256613 | BHU, Varanasi, Uttar Pradesh | 95.58 | 11.83 | 17.53 | 102 | 12.04 |
| 272 | IC 256616 | BHU, Varanasi, Uttar Pradesh | 87.58 | 7.00 | 17.87 | 97 | 14.81 |
| 273 | IC 256617 | BHU, Varanasi, Uttar Pradesh | 94.65 | 6.17 | 17.83 | 100 | 11.11 |
| 274 | IC 256754 | BHU, Varanasi, Uttar Pradesh | 121.18 | 6.33 | 21.95 | 102 | 10.19 |
| 275 | IC 256807 | BHU, Varanasi, Uttar Pradesh | 48.43 | 5.67 | 13.93 | 96 | 30.09 |
| 276 | IC 260917 | BHU, Varanasi, Uttar Pradesh | 81.25 | 13.67 | 14.45 | 100 | 11.57 |
| 277 | IC 264141 | BHU, Varanasi, Uttar Pradesh | 64.62 | 9.50 | 18.62 | 104 | 24.54 |
| 278 | IC 264151 | BHU, Varanasi, Uttar Pradesh | 118.88 | 8.67 | 23.05 | 126 | 9.26 |
| 279 | IC 274377 | BHU, Varanasi, Uttar Pradesh | 128.15 | 4.50 | 23.00 | 109 | 11.11 |
| 280 | IC 274408 | BHU, Varanasi, Uttar Pradesh | 142.32 | 8.83 | 25.65 | 109 | 9.72 |
| 281 | IC 277248 | BHU, Varanasi, Uttar Pradesh | 67.60 | 2.50 | 12.55 | 102 | 13.24 |
| 282 | IC 277261 | BHU, Varanasi, Uttar Pradesh | 96.78 | 7.17 | 16.95 | 103 | 8.80 |
| 283 | IC 277266 | BHU, Varanasi, Uttar Pradesh | 128.26 | 4.17 | 26.30 | 112 | 10.65 |
| 284 | IC 277274 | BHU, Varanasi, Uttar Pradesh | 83.10 | 6.83 | 17.37 | 104 | 10.19 |
| 285 | IC 277284 | BHU, Varanasi, Uttar Pradesh | 63.65 | 9.50 | 18.10 | 102 | 11.57 |
| 286 | IC 277290 | BHU, Varanasi, Uttar Pradesh | 116.77 | 3.50 | 20.55 | 103 | 10.19 |
| 287 | IC 277330 | BHU, Varanasi, Uttar Pradesh | 74.93 | 8.50 | 19.40 | 101 | 11.57 |
| 288 | IC 277332 | BHU, Varanasi, Uttar Pradesh | 86.52 | 3.00 | 17.30 | 100 | 6.94 |
| 289 | IC 278777 | BHU, Varanasi, Uttar Pradesh | 85.30 | 5.33 | 17.37 | 98 | 7.87 |
| 290 | IC 279355 | BHU, Varanasi, Uttar Pradesh | 58.57 | 12.50 | 17.47 | 100 | 15.28 |
| 291 | IC 280478 | BHU, Varanasi, Uttar Pradesh | 94.42 | 10.17 | 17.00 | 96 | 8.80 |
| 292 | IC 280504 | BHU, Varanasi, Uttar Pradesh | 66.48 | 8.33 | 16.03 | 96 | 14.81 |
| 293 | IC 280528 | BHU, Varanasi, Uttar Pradesh | 90.27 | 3.50 | 19.18 | 101 | 8.80 |
| 294 | IC 280564 | BHU, Varanasi, Uttar Pradesh | 73.88 | 2.50 | 15.77 | 96 | 13.43 |
| 295 | IC 281508 | BHU, Varanasi, Uttar Pradesh | 83.10 | 6.83 | 24.58 | 102 | 12.96 |
| 296 | IC 281783 | BHU, Varanasi, Uttar Pradesh | 109.65 | 6.17 | 23.23 | 119 | 6.94 |
| 297 | IC 281786 | BHU, Varanasi, Uttar Pradesh | 61.83 | 8.00 | 19.98 | 92 | 20.83 |
| 298 | IC 282438 | BHU, Varanasi, Uttar Pradesh | 81.88 | 5.50 | 16.78 | 98 | 11.11 |
| 299 | IC 282460 | BHU, Varanasi, Uttar Pradesh | 113.42 | 6.50 | 20.92 | 119 | 5.09 |
| 300 | IC 282463 | BHU, Varanasi, Uttar Pradesh | 67.68 | 5.17 | 14.17 | 105 | 19.91 |
| 301 | IC 282466 | BHU, Varanasi, Uttar Pradesh | 73.00 | 8.83 | 19.38 | 104 | 17.13 |
| 302 | IC 282473 | BHU, Varanasi, Uttar Pradesh | 126.38 | 5.50 | 24.78 | 95 | 9.26 |
| 303 | IC 282480 | BHU, Varanasi, Uttar Pradesh | 131.93 | 3.50 | 26.68 | 97 | 10.19 |
| 304 | IC 282500 | BHU, Varanasi, Uttar Pradesh | 87.52 | 9.17 | 19.20 | 101 | 12.50 |
| 305 | IC 282512 | BHU, Varanasi, Uttar Pradesh | 102.63 | 6.67 | 20.78 | 93 | 10.65 |
| 306 | IC 282526 | BHU, Varanasi, Uttar Pradesh | 121.92 | 5.33 | 21.43 | 92 | 9.72 |
| 307 | IC 282808 | BHU, Varanasi, Uttar Pradesh | 108.50 | 3.50 | 22.35 | 104 | 9.26 |
| 308 | IC 282812 | BHU, Varanasi, Uttar Pradesh | 77.48 | 7.83 | 17.90 | 102 | 10.49 |
| 309 | IC 282815 | BHU, Varanasi, Uttar Pradesh | 73.02 | 8.33 | 19.82 | 101 | 8.33 |
| 310 | IC 283023 | BHU, Varanasi, Uttar Pradesh | 68.55 | 2.50 | 11.82 | 100 | 14.81 |
| 311 | IC 283026 | BHU, Varanasi, Uttar Pradesh | 77.08 | 2.33 | 14.72 | 101 | 4.17 |
| 312 | IC 283028 | BHU, Varanasi, Uttar Pradesh | 111.28 | 6.33 | 22.12 | 119 | 4.17 |
| 313 | IC 283038 | BHU, Varanasi, Uttar Pradesh | 122.08 | 8.00 | 22.20 | 119 | 8.80 |
| 314 | IC 283041 | BHU, Varanasi, Uttar Pradesh | 119.72 | 6.17 | 25.33 | 120 | 9.26 |
| 315 | IC 283139 | BHU, Varanasi, Uttar Pradesh | 66.08 | 9.67 | 14.77 | 95 | 20.37 |
| 316 | IC 283204 | BHU, Varanasi, Uttar Pradesh | 95.87 | 11.67 | 18.48 | 99 | 8.80 |
| 317 | IC 256538 | BHU, Varanasi, Uttar Pradesh | 78.48 | 6.67 | 17.38 | 85 | 9.26 |
| 318 | IC 277267 | BHU, Varanasi, Uttar Pradesh | 63.82 | 12.00 | 17.75 | 95 | 12.96 |
| 319 | IC 277275 | BHU, Varanasi, Uttar Pradesh | 67.45 | 4.00 | 17.90 | 97 | 14.81 |
| 320 | IC 281774 | BHU, Varanasi, Uttar Pradesh | 69.02 | 12.50 | 17.17 | 128 | 12.04 |
| 321 | IC 256515 | BHU, Varanasi, Uttar Pradesh | 69.17 | 3.83 | 14.42 | 98 | 11.11 |
| 322 | IC 282508 | BHU, Varanasi, Uttar Pradesh | 67.45 | 4.00 | 17.90 | 97 | 14.81 |
| 323 | IC 283187 | BHU, Varanasi, Uttar Pradesh | 117.08 | 6.50 | 23.82 | 106 | 9.26 |
| 324 | IC 283206 | BHU, Varanasi, Uttar Pradesh | 95.99 | 11.00 | 16.85 | 100 | 27.68 |
| 325 | CO 39 | TNAU, Coimbatore, Tamil Nadu | 117.08 | 6.50 | 23.82 | 106 | 28.58 |
| 326 | Tetep | BHU, Varanasi, Uttar Pradesh | 113.05 | 10.07 | 20.74 | 95 | 9.13 |
| 327 | Pusa Basmati-1 | BHU, Varanasi, Uttar Pradesh | 88.10 | 11.20 | 24.51 | 112 | 19.54 |
| 328 | Tapaswini | NRRI, Cuttack, Odisha | 114.65 | 13.28 | 19.86 | 98 | 38.37 |
| 329 | Jasmine 85 | NRRI, Cuttack, Odisha | 100.00 | 8.78 | 21.56 | 120 | 8.18 |
| 330 | Teqing | BHU, Varanasi, Uttar Pradesh | 73.68 | 8.92 | 21.38 | 95 | 16.20 |

**Supplementary Table 2.** List of SSR primers used in the present study to understand genetic diversity, structure and association mapping to identify the loci for resistance against sheath blight disease in rice.

| **Sl. No** | **SSR primer** | **Forward primer** | **Reverse primer** | **Chr. No.** | **Annealing Temperature (^o^C)** | **Product size** |
| --- | --- | --- | --- | --- | --- | --- |
| 1 | RM11229 | TGACAGAAACAAAGCGGAAGG | TCCAAACCGCTATTCTTGTAGC | 1 | 60 | 393 |
| 2 | RM306 | CAAGGTCAAGAATGCAATGG | GCCACTTTAATCATTGCATC | 1 | 52 | 155 |
| 3 | RM237 | CAAATCCCGACTGCTGTCC | TGGGAAGAGAGCACTACAGC | 1 | 55 | 130 |
| 4 | RM1232 | GTCTCTGTGGAGTGGAAGCC | TTCACCGGATCTGATTACCC | 1 | 55 | 112 |
| 5 | RM1216 | TTCCCCAATGGAACAGTGAC | AGGGTCTACCACCCGATCTC | 1 | 55 | 84 |
| 6 | RM3825 | AAAGCCCCCAAAAGCAGTAC | GTGAAACTCTGGGGTGTTCG | 1 | 55 | 147 |
| 7 | RM1339 | ATCAAAGCATGTAAACCAGC | CGTAAGATCTCCCTACCACC | 1 | 55 | 144 |
| 8 | RM1361 | ATTCTCTCCGCCTAAACAAC | TTCTCGTGCACAGTTAATACC | 1 | 60 | 214 |
| 9 | RM6292 | GAGTCCTTCCTAGCTTCCTCG | AGTCCCAGAAGCTGCTCAAG | 1 | 55 | 198 |
| 10 | RM3482 | TTGTTGTCAAGCTACGGTGG | CTGCTTCGTGATGTTGTTGG | 1 | 55 | 101 |
| 11 | RM14 | CCGAGGAGAGGAGTTCGAC | GTGCCAATTTCCTCGAAAAA | 1 | 52 | 191 |
| 12 | RM12253 | TCTGGAGGTTGAAAGCTGAAACG | CGACTATGTCGGTGCCAAATGC | 1 | 59 | 351 |
| 13 | RM109 | GCCGCCGGAGAGGGAGAGAGAG | CCCCGACGGGATCTCCATCGTC | 2 | 55 | 97 |
| 14 | RM5529 | AGCCGAAACTACATTCGGTG | TTGTGTAGTTGGCACGCTTC | 2 | 55 | 167 |
| 15 | RM424 | TTTGTGGCTCACCAGTTGAG | TGGCGCATTCATGTCATC | 2 | 55 | 239 |
| 16 | RM341 | CAAGAAACCTCAATCCGAGC | CTCCTCCCGATCCCAATC | 2 | 55 | 172 |
| 17 | RM1385 | ATGACAGGTAAGGTGTGGTG | TGAACATCATCTTCGAATCC | 2 | 58 | 176 |
| 18 | RM3685 | AAACGTATTAGTGCGCCCAG | TCTCGCTTCTCCTCCTCTCC | 2 | 55 | 115 |
| 19 | RM3857 | TTCTTGGTATGCCGCGTG | GAGCCTCTCCCTCTCCTCTC | 2 | 55 | 137 |
| 20 | RM112 | GGGAGGAGAGGCAAGCGGAGAG | AGCCGGTGCAGTGGACGGTGAC | 2 | 55 | 128 |
| 21 | RM250 | GGTTCAAACCAAGCTGATCA | GATGAAGGCCTTCCACGCAG | 2 | 55 | 153 |
| 22 | RM3894 | TATGCTCTCTCCTTCAGGCC | CTTACCAACTCCGCACTTGC | 3 | 55 | 201 |
| 23 | RM22 | GGTTTGGGAGCCCATAATCT | CTGGGCTTCTTTCACTCGTC | 3 | 55 | 194 |
| 24 | RM569 | GACATTCTCGCTTGCTCCTC | TGTCCCCTCTAAAACCCTCC | 3 | 55 | 175 |
| 25 | RM81 | GAGTGCTTGTGCAAGATCCA | CTTCTTCACTCATGCAGTTC | 3 | 58 | 110 |
| 26 | RM3117 | GCCATCTCTCTCTCTCTCTCTC | CCTTAGCTCATCAAGCGAGG | 3 | 55 | 111 |
| 27 | RM5474 | AAAGTGTTGGTGAGCATAGC | TTTGTGTTTGGAGAGACGAG | 3 | 55 | 155 |
| 28 | RM338 | CACAGGAGCAGGAGAAGAGC | GGCAAACCGATCACTCAGTC | 3 | 55 | 183 |
| 29 | RM251 | GAATGGCAATGGCGCTAG | ATGCGGTTCAAGATTCGATC | 3 | 55 | 147 |
| 30 | RM16 | CGCTAGGGCAGCATCTAAA | AACACAGCAGGTACGCGC | 3 | 55 | 181 |
| 31 | RM5626 | GCAGACGAGATGAGATCG | GTAGAGGATGGGCAGCAG | 3 | 55 | 188 |
| 32 | RM426 | ATGAGATGAGTTCAAGGCCC | AACTCTGTACCTCCATCGCC | 3 | 55 | 150 |
| 33 | RM1350 | CGCCCTAGTAGATAGGTAATTG | AAATCAGCAAGAAAGCTCTG | 3 | 56 | 167 |
| 34 | RM514 | AGATTGATCTCCCATTCCCC | CACGAGCATATTACTAGTGG | 3 | 55 | 259 |
| 35 | RM570 | GTTCTTCAACTCCCAGTGCG | TGACGATGTGGAAGAGCAAG | 3 | 55 | 208 |
| 36 | RM16200 | GTGGTAGGGCGAAATGATCTGC | ATCACGCGCTCCTACCTCACC | 3 | 55 | 193 |
| 37 | RM85 | CCAAAGATGAAACCTGGATTG | GCACAAGGTGAGCAGTCC | 3 | 60 | 107 |
| 38 | RM551 | AGCCCAGACTAGCATGATTG | GAAGGCGAGAAGGATCACAG | 4 | 55 | 192 |
| 39 | RM335 | GTACACACCCACATCGAGAAG | GCTCTATGCGAGTATCCATGG | 4 | 55 | 104 |
| 40 | RM518 | CTCTTCACTCACTCACCATGG | ATCCATCTGGAGCAAGCAAC | 4 | 55 | 171 |
| 41 | RM185 | AGTTGTTGGGAGGGAGAAAGGCC | AGGAGGCGACGGCGATGTCCTC | 4 | 55 | 197 |
| 42 | RM317 | CATACTTACCAGTTCACCGCC | CTGGAGAGTGTCAGCTAGTTGA | 4 | 55 | 155 |
| 43 | RM5709 | CTGAATTTATTATAGGACGGAAG | CATAGTATTGGATTGGACACG | 4 | 55 | 163 |
| 44 | RM8217 | ACTAGCGATGTCTGAGTTGAC | TATTCACATGCTTGCTCATC | 4 | 59 | 178 |
| 45 | RM5478 | ATCCAATGCGATGCTACTCC | CATCACGAGACCACGACAAG | 4 | 55 | 130 |
| 46 | RM507 | CTTAAGCTCCAGCCGAAATG | CTCACCCTCATCATCGCC | 5 | 53 | 258 |
| 47 | RM159 | GGGGCACTGGCAAGGGTGAAGG | GCTTGTGCTTCTCTCTCTCTCTCTCTCTC | 5 | 55 | 248 |
| 48 | RM169 | TGGCTGGCTCCGTGGGTAGCTG | TCCCGTTGCCGTTCATCCCTCC | 5 | 55 | 167 |
| 49 | RM13 | TCCAACATGGCAAGAGAGAG | GGTGGCATTCGATTCCAG | 5 | 58 | 141 |
| 50 | RM146 | CTATTATTCCCTAACCCCCATACCCTCC | AGAGCCACTGCCTGCAAGGCCC | 5 | 55 | 345 |
| 51 | RM164 | TCTTGCCCGTCACTGCAGATATCC | GCAGCCCTAATGCTACAATTCTTC | 5 | 55 | 246 |
| 52 | RM39 | GCCTCTCTCGTCTCCTTCCT | AATTCAAACTGCGGTGGC | 5 | 55 | 115 |
| 53 | RM173 | CCTACCTCGCGATCCCCCCCTC | CCATGAGGAGGAGGCGGCGATC | 5 | 55 | 186 |
| 54 | RM188 | TCCGCCTCTCCTCTCGCTTCCC | GCAACGCACAACCGAACCGAGC | 5 | 55 | 210 |
| 55 | RM178 | TCGCGTGAAAGATAAGCGGCGC | GATCACCGTTCCCTCCGCCTGC | 5 | 55 | 117 |
| 56 | RM274 | CCTCGCTTATGAGAGCTTCG | CTTCTCCATCACTCCCATGG | 5 | 55 | 160 |
| 57 | RM5784 | GAACGCACAAACGTCCATTC | TTCACTCCAGTTCCTCCACC | 5 | 55 | 138 |
| 58 | RM334 | GTTCAGTGTTCAGTGCCACC | GACTTTGATCTTTGGTGGACG | 5 | 55 | 182 |
| 59 | RM3286 | AGTACCCTGCCACGGTACAG | CATGCTGAAGTAAAACCGGG | 5 | 55 | 161 |
| 60 | RM133 | TTGGATTGTTTTGCTGGCTCGC | GGAACACGGGGTCGGAAGCGAC | 6 | 55 | 230 |
| 61 | RM435 | ATTACGTGCATGTCTGGCTG | CGTACCTGACCATGCATCTG | 6 | 60 | 166 |
| 62 | RM190 | GCTACAAATAGCCACCCACACC | CAACACAAGCAGAGAAGTGAAGC | 6 | 55 | 124 |
| 63 | RM6917 | ATAGCTGTCCACTCCCCTTG | TTTTCTGAACTCCGTAGCCC | 6 | 55 | 186 |
| 64 | RM253 | TCCTTCAAGAGTGCAAAACC | GCATTGTCATGTCGAAGCC | 6 | 58 | 141 |
| 65 | RM2615 | CAGAGTGCTTTAGACAATCA | AAATTGGTAAGAGATTCTGC | 6 | 57 | 164 |
| 66 | RM5850 | TTAGGTGTGTGAGCGTGGC | ATACACAGATGACGCACACG | 6 | 55 | 181 |
| 67 | RM1161 | AAACTGTTTTACCCCTGGCC | ATCCCCTTCTGCGGTAAAAC | 6 | 60 | 80 |
| 68 | RM6395 | CTTCGGCTTCTGAACTAGCG | CAGTGCCGATGATCCTCTTC | 6 | 55 | 110 |
| 69 | RM400 | ACACCAGGCTACCCAAACTC | CGGAGAGATCTGACATGTGG | 6 | 55 | 321 |
| 70 | RM427 | TCACTAGCTCTGCCCTGACC | TGATGAGAGTTGGTTGCGAG | 7 | 55 | 185 |
| 71 | RM214 | CTGATGATAGAAACCTCTTCTC | AAGAACAGCTGACTTCACAA | 7 | 55 | 112 |
| 72 | RM5481 | GGCACAGAGTAGTGATGTTTCG | TGAAGCTCCAATACTCTCCC | 7 | 55 | 163 |
| 73 | RM3691 | GCTGATGGTCAAAGATCAGG | ATGTGTCTGCTGGCACAGAG | 7 | 55 | 117 |
| 74 | RM21693 | GCACAGACCAGAACTTTCTTCG | TGGCGAGTGTAGATGTAATTGG | 7 | 55 | 462 |
| 75 | RM336 | CTTACAGAGAAACGGCATCG | GCTGGTTTGTTTCAGGTTCG | 7 | 55 | 154 |
| 76 | RM21792 | GTGCAGAAGGCAAATATGAACACG | AGACGAACGGTCAAACATGTGC | 7 | 55 | 197 |
| 77 | RM6152 | GAATTCACCGCTCTCCAGTC | AGGAGGATCTCCTCCAGGAG | 7 | 55 | 206 |
| 78 | RM10 | TTGTCAAGAGGAGGCATCG | CAGAATGGGAAATGGGTCC | 7 | 60 | 159 |
| 79 | RM1335 | GCATGCATGAATATGATGG | AGATCGAACAAGAAGAGTGG | 7 | 55 | 168 |
| 80 | RM408 | CAACGAGCTAACTTCCGTCC | ACTGCTACTTGGGTAGCTGACC | 8 | 55 | 128 |
| 81 | RM1235 | AGCAGAGGAGGAGATGATGG | GGACCAAAACGAAGCTATCC | 8 | 55 | 118 |
| 82 | RM38 | ACGAGCTCTCGATCAGCCTA | TCGGTCTCCATGTCCCAC | 8 | 55 | 250 |
| 83 | RM5647 | ACTCCGACTGCAGTTTTTGC | AACTTGGTCGTGGACAGTGC | 8 | 55 | 119 |
| 84 | RM5428 | ATGCAATACAGCACACTCGC | CTTATGCTCTCATGGCTCCC | 8 | 55 | 222 |
| 85 | RM310 | CCAAAACATTTAAAATATCATG | GCTTGTTGGTCATTACCATTC | 8 | 49 | 105 |
| 86 | RM210 | TCACATTCGGTGGCATTG | CGAGGATGGTTGTTCACTTG | 8 | 55 | 140 |
| 87 | RM3452 | GGCAGCCCATCAACTAGATC | TTGCAAACCCTAGTCCAAGC | 8 | 55 | 190 |
| 88 | RM105 | GTCGTCGACCCATCGGAGCCAC | TGGTCGAGGTGGGGATCGGGTC | 9 | 55 | 134 |
| 89 | RM24260 | GATCTCTCACCCACATGCCTAGC | TCCCATCTTGATCGATCTCTTCG | 9 | 55 | 244 |
| 90 | RM409 | CCGTCTCTTGCTAGGGATTC | GGGGTGTTTTGCTTTCTCTG | 9 | 60 | 96 |
| 91 | RM434 | GCCTCATCCCTCTAACCCTC | CAAGAAAGATCAGTGCGTGG | 9 | 55 | 152 |
| 92 | RM257 | CAGTTCCGAGCAAGAGTACTC | GGATCGGACGTGGCATATG | 9 | 55 | 147 |
| 93 | RM3533 | TTCCAACCTGTCAGGGAATC | CATTTCCCTTCCCTCTCCTC | 9 | 55 | 120 |
| 94 | RM242 | GGCCAACGTGTGTATGTCTC | TATATGCCAAGACGGATGGG | 9 | 55 | 225 |
| 95 | RM6251 | GCAGAAATGCTGAGTTGCTG | TCCCATTCCTCGCTCATTC | 9 | 55 | 118 |
| 96 | RM160 | AGCTAGCAGCTATAGCTTAGCTGGAGATCG | TCTCATCGCCATGCGAGGCCTC | 9 | 55 | 131 |
| 97 | RM215 | CAAAATGGAGCAGCAAGAGC | TGAGCACCTCCTTCTCTGTAG | 9 | 60 | 148 |
| 98 | RM6971 | TTTGCGAACTAGACAAGGCC | GCGTCATTCTCGACGAGC | 9 | 55 | 202 |
| 99 | RM3823 | CTCCTTCAGTCGGTCGTC | AAGGAGTCTGTCGCTTTACC | 9 | 55 | 192 |
| 100 | RM245 | ATGCCGCCAGTGAATAGC | CTGAGAATCCAATTATCTGGGG | 9 | 47 | 150 |
| 101 | RM205 | CTGGTTCTGTATGGGAGCAG | CTGGCCCTTCACGTTTCAGTG | 9 | 55 | 122 |
| 102 | RM3744 | CCTAAGAGGAGCCATCTAACAACTGG | CTTGGATATACTGGCCCTTCACG | 9 | 55 | 151 |
| 103 | RM6364 | GTTCATTTCGTCCTTCTCGG | TCTCGATTCTTCCTTCTCCG | 10 | 55 | 163 |
| 104 | RM8015 | AAGTTTCTCCAAGCCAAGAG | AATGTGTTTTCCTGGTCAGA | 10 | 55 | 130 |
| 105 | RM8207 | TTCATCGACATCATCAACTG | CAGTTTGGGATGAAGTGTTC | 10 | 55 | 191 |
| 106 | RM4455 | CTCTCAAAGAACTAGGACTC | GAGAAGGTATGATAACCAAT | 10 | 55 | 116 |
| 107 | RM467 | GGTCTCTCTCTCTCTCTCTCTCTC | CTCCTGACAATTCAACTGCG | 10 | 55 | 221 |
| 108 | RM5304 | CAGCCCATCTCTCTCCTCTG | GATAGCAGGAAGAGGCGTTG | 10 | 55 | 143 |
| 109 | RM5392 | GCCGTCTACCTCATGGTCAC | CCATAACCGTGGTGTTTGAG | 10 | 55 | 149 |
| 110 | RM147 | TACGGCTTCGGCGGCTGATTCC | CCCCCGAATCCCATCGAAACCC | 10 | 55 | 97 |
| 111 | RM25790 | TCTGAGGGATGATTGACAACTCG | CGCTCTCTGCACTTTCACCTAGC | 10 | 55 | 247 |
| 112 | RM590 | CATCTCCGCTCTCCATGC | GGAGTTGGGGTCTTGTTCG | 10 | 55 | 137 |
| 113 | RM7203 | ACGTGGTGCCTTCTTTCAAG | CTCTGGCCTTCTACTCATGG | 11 | 60 | 106 |
| 114 | RM536 | TCTCTCCTCTTGTTTGGCTC | ACACACCAACACGACCACAC | 11 | 55 | 243 |
| 115 | RM202 | CAGATTGGAGATGAAGTCCTCC | CCAGCAAGCATGTCAATGTA | 11 | 55 | 189 |
| 116 | RM3428 | ATTCATGCTTCCTTTCAGTG | GATTACTGGTTTGCCATTTG | 11 | 55 | 156 |
| 117 | RM209 | ATATGAGTTGCTGTCGTGCG | CAACTTGCATCCTCCCCTCC | 11 | 60 | 134 |
| 118 | RM254 | AGCCCCGAATAAATCCACCT | CTGGAGGAGCATTTGGTAGC | 11 | 60 | 165 |
| 119 | RM1233 | TTCGTTTTCCTTGGTTAGTG | ATTGGCTCCTGAAGAAGG | 11 | 55 | 175 |
| 120 | RM224 | ATCGATCGATCTTCACGAGG | TGCTATAAAAGGCATTCGGG | 11 | 60 | 157 |
| 121 | sbq1 | ACAAATGGAACACTCCAGCC | GGACAAGAAGCTCGAGGACA | 11 | 55 | 232 |
| 122 | sbq11 | CCCGTGCATTGTAACTCCTT | AGCCACAGGTTACCAGGTTG | 11 | 55 | 154 |
| 123 | K39512 | GCCACATCAATGGCTACAACGTC | CCAGAATTTACAGGCTCTGG | 11 | 60 | 112 |
| 124 | RM7443 | TGCTGCGTGTTACTTTGGTG | AACCCTTCATCAGGCTACGC | 11 | 55 | 151 |
| 125 | Sbq33 | GTTCGATCCAACACGAGAGG | CGAGCATCTTGCTACCCAGT | 11 | 60 | 257 |
| 126 | RM1880 | ACCACTAAATAAGCACATAC | GGCATCATACATTAAAATAC | 12 | 49 | 128 |
| 127 | RM20A | ATCTTGTCCCTGCAGGTCAT | GAAACAGAGGCACATTTCATTG | 12 | 55 | 140 |
| 128 | RM247 | TAGTGCCGATCGATGTAACG | CATATGGTTTTGACAAAGCG | 12 | 55 | 131 |
| 129 | RM101 | GTGAATGGTCAAGTGACTTAGGTGGC | ACACAACATGTTCCCTCCCATGC | 12 | 55 | 324 |
| 130 | RM1337 | GTGCAATGCTGAGGAGTATC | CTGAGAATCTGGAGTGCTTG | 12 | 55 | 210 |
| 131 | RM5364 | GTATTACGCTCGATAGCGGC | GTATCCTTTCTCGCAATCGC | 12 | 55 | 148 |
| 132 | RM28130 | CAGCAGACGTTCCGGTTCTACTCG | AGGACGGTGGTGGTGATCTGG | 12 | 55 | 175 |
| 133 | RM7025 | TGCGAAGTAACAAGCCTGTG | GCAAAGGTTGTGTGAAGGAG | 12 | 58 | 81 |

**Supplementary Table 3.** Descriptive statistics of different traits of core panel population over the season

| **Trait** | **Mean** | **Median** | **Skewness** | **Kurtosis** | **CV (%)** |
| --- | --- | --- | --- | --- | --- |
| Days_50%_Flowering | 95.11 | 91 | 1.53 | 2.01 | 14.335 |
| Plant_height | 110.18 | 112.49 | -0.62 | 1.01 | 12.26 |
| Panicle_lth | 21.57 | 21.56 | 0.44 | 1.02 | 10.26 |
| Tiller_no_plant | 10.67 | 10.46 | 0.65 | 1.08 | 23.49 |
| Yield_plant | 10.55 | 10.28 | 0.15 | -0.09 | 32.22 |
| Internodal_lth | 27.26 | 27.02 | 0.14 | 1.04 | 14.20 |
| Flag_leaf_lth | 27.37 | 27.27 | 0.06 | 0.59 | 16.79 |
| Flag_leaf_wd | 1.29 | 1.32 | -0.19 | 0.65 | 12.33 |
| Culm_thick | 4.71 | 4.39 | 4.90 | 31.49 | 40.42 |
| Ligule_col | 1.09 | 1 | 3.92 | 15.54 | 33 |
| Ligule_Shape | 1.83 | 2 | -1.85 | 1.45 | 20.06 |
| Auricle_col | 1.03 | 1 | 4.98 | 23.09 | 18.13 |
| Basal_Leafsheath_col | 1.17 | 1 | 3.29 | 10.50 | 46.80 |
| Apiculus_col | 2.80 | 2 | 0.85 | -0.56 | 72.53 |
| PDI_7th_day | 12.08 | 11.11 | 2.14 | 5.93 | 14.48 |
| PDI_14th_day | 20.92 | 20 | 0.49 | -0.12 | 26.29 |
| PDI_21st_day | 31.51 | 31.11 | 0.08 | 0.56 | 19.68 |
| PDI_28th_day | 40.18 | 39.36 | 0.21 | 0.80 | 25.95 |
| PDI(mean) | 26.17 | 25.28 | 0.42 | 0.10 | 19.38 |
| AUDPC(mean) | 775.86 | 755.90 | 0.41 | 0.13 | 19.07 |

**Supplementary Table 4. Descriptive statistics of different traits of panel population during dry season 2019**

| **Trait** | **Mean** | **Median** | **Skewness** | **Kurtosis** | **CV (%)** |
| --- | --- | --- | --- | --- | --- |
| Days_50%_Flow. | 95.57 | 91.5 | 1.58 | 2.30 | 14.55 |
| Plant_hheight | 109.65 | 111.31 | -0.55 | 0.29 | 12.52 |
| Panicle_lth | 22.09 | 22 | -0.03 | -0.05 | 11.60 |
| Tiller_no_plant | 10.86 | 10.4 | 0.69 | 1.07 | 30.37 |
| Yield_plant | 11.08 | 10.63 | 0.18 | -0.75 | 40.87 |
| PDI_7th_day | 12.33 | 11.11 | 2.05 | 6.37 | 20.54 |
| PDI_14th_day | 22.75 | 22.445 | 0.34 | -0.53 | 31.06 |
| PDI_21st_day | 33.33 | 33.33 | 0.45 | 1.47 | 22.06 |
| Mean_PDI | 22.81 | 22.59 | 0.52 | 0.26 | 21.34 |
| AUDPC | 441.99 | 435.56 | 0.47 | -0.21 | 23.32 |

**Supplementary Table 5.** Descriptive statistics of different traits of panel population during wet season 2019

| **Trait** | **Mean** | **Median** | **Skewness** | **Kurtosis** | **CV (%)** |
| --- | --- | --- | --- | --- | --- |
| Days_50%_Flo | 94.11 | 90 | 1.46 | 1.69 | 14.35 |
| Plant_height | 110.70 | 110.3 | 0.007 | 0.51 | 18.18 |
| Panicle_length | 21.05 | 20.75 | 0.65 | 0.92 | 14.61 |
| Tiller_no_plant | 10.48 | 10.4 | 1.13 | 3.34 | 34.03 |
| Yield_plant | 10.01 | 8.97 | 0.41 | -0.66 | 52.09 |
| Internodal_lth | 27.26 | 27.02 | 0.14 | 1.04 | 14.20 |
| Flag_leaf_lth | 27.37 | 27.27 | 0.06 | 0.60 | 16.79 |
| Flag_leaf_wd | 1.29 | 1.32 | -0.19 | 0.65 | 12.33 |
| Culm_thick | 4.71 | 4.39 | 4.90 | 31.49 | 40.42 |
| Ligule_colour | 1.09 | 1 | 3.92 | 15.54 | 33 |
| Ligule_Shape | 1.83 | 2 | -1.85 | 1.45 | 20.06 |
| Auricle_col | 1.03 | 1 | 4.98 | 23.09 | 18.13 |
| Basal_Leafsheath_colour | 1.17 | 1 | 3.29 | 10.50 | 46.80 |
| Apiculus_col | 2.80 | 2 | 0.85 | -0.57 | 72.53 |
| PDI_7th_day | 11.83 | 11.11 | 2.89 | 8.66 | 15.92 |
| PDI_14th_day | 19.09 | 16.67 | 1.09 | 0.91 | 39.72 |
| PDI_21st_day | 29.69 | 28.89 | 0.10 | 0.05 | 29.45 |
| PDI_28th_day | 40.19 | 39.36 | 0.21 | 0.80 | 25.95 |
| PDI(mean) | 25.20 | 24.12 | 0.59 | 0.06 | 25.04 |
| AUDPC(mean) | 735.76 | 700 | 0.63 | 0.14 | 26.75 |

**Supplementary Table 6.** Heritability and genetic advance for morphological and sheath blight related traits of 192 rice germplasm on dry and wet seasons.

| **Traits** | **Season 1 (dry season 2019)** | | | **Season 2 (wet season 2019)** | | |
| --- | --- | --- | --- | --- | --- | --- |
|  | **h^2^_bs_** | **GA** | **Mean Square** | **h^2^_bs_** | **GA** | **Mean square** |
| 7th Day PDI | 1.43 | 0.10 | 12.31^ns^ | 4.55 | 0.27 | 12.17^ns^ |
| 14th Day PDI | 18.15 | 2.76 | 21.23** | 26.48 | 4.90 | 19.44*** |
| 21st Day PDI | 21.75 | 3.76 | 31.83** | 22.42 | 4.70 | 29.55** |
| 28th Day PDI | NA | NA | NA | 27.33 | 6.64 | 40.16*** |
| Mean PDI | 20.94 | 2.30 | 21.79** | 31.77 | 5.03 | 24.85*** |
| AUDPC | 19.73 | 44.94 | 420.46** | 37.68 | 53.93 | 721.66*** |
| plant height | 36.59 | 12.31 | 109.84*** | 42.58 | 19.82 | 109.52*** |
| PL | 42.42 | 2.53 | 21.89*** | 29.10 | 2.21 | 21.56*** |
| NT | 33.67 | 2.37 | 10.53*** | 10.88 | 0.87 | 10.43^ns^ |
| IL | - | - | - | 42.73 | 4.10 | 27.63*** |
| FL | - | - | - | 38.93 | 4.28 | 27.28*** |
| FW | - | - | - | 3.63 | 0.02 | 1.30^ns^ |
| CT | - | - | - | 24.05 | 0.49 | 4.41*** |
| GY | 45.05 | 2.06 | 11.20^ns^ | 40.01 | 6.26 | 10.71*** |

*** significance value at 0.001%, ** significance value at 0.01%, * significance value at 0.05% and ns- non significance. h^2^bs –heritability broad sense, GA-genetic advance, PDI-percent disease index, AUDPC-area under disease progress curve, PL-panicle length, NT-tiller number per plant, IL-internodal length, FL- flag leaf length, FW-flag leaf width, CT-culm thickness, GY- grain yield.

**Supplementary Table 7.** Details of SSR primers used for genotyping the core panel population consisting 192 genotypes and their estimated molecular genetic diversity parameters.

| **Sl. No** | **Marker** | **Chr. No.** | **No. of Allele** | **Minimum Size of alleles (bp)** | **Maximum size of alleles (bp)** | **Major Allele Frequency** | **Gene Diversity** | **Heterozygosity** | **PIC** | **Inbreeding coefficient** |
| --- | --- | --- | --- | --- | --- | --- | --- | --- | --- | --- |
| 1 | RM11229 | 1 | 3 | 300 | 400 | 0.485 | 0.581 | 0.000 | 0.491 | 1.000 |
| 2 | RM306 | 1 | 2 | 155 | 175 | 0.601 | 0.479 | 0.013 | 0.365 | 0.974 |
| 3 | RM237 | 1 | 2 | 130 | 140 | 0.592 | 0.483 | 0.000 | 0.366 | 1.000 |
| 4 | RM1232 | 1 | 2 | 112 | 130 | 0.728 | 0.396 | 0.006 | 0.318 | 0.986 |
| 5 | RM1216 | 1 | 4 | 70 | 100 | 0.374 | 0.708 | 0.000 | 0.655 | 1.000 |
| 6 | RM3825 | 1 | 4 | 130 | 160 | 0.448 | 0.611 | 0.007 | 0.529 | 0.988 |
| 7 | RM1339 | 1 | 3 | 130 | 150 | 0.508 | 0.562 | 0.005 | 0.467 | 0.990 |
| 8 | RM1361 | 1 | 2 | 195 | 210 | 0.723 | 0.401 | 0.000 | 0.321 | 1.000 |
| 9 | RM6292 | 1 | 3 | 200 | 200 | 0.597 | 0.515 | 0.006 | 0.422 | 0.989 |
| 10 | RM3482 | 1 | 5 | 90 | 130 | 0.280 | 0.782 | 0.000 | 0.747 | 1.000 |
| 11 | RM14 | 1 | 3 | 170 | 190 | 0.654 | 0.512 | 0.074 | 0.459 | 0.856 |
| 12 | RM12253 | 1 | 2 | 351 | 361 | 0.741 | 0.383 | 0.000 | 0.310 | 1.000 |
| 13 | RM109 | 2 | 2 | 97 | 107 | 0.931 | 0.129 | 0.000 | 0.121 | 1.000 |
| 14 | RM5529 | 2 | 2 | 160 | 170 | 0.876 | 0.217 | 0.000 | 0.194 | 1.000 |
| 15 | RM424 | 2 | 3 | 240 | 270 | 0.513 | 0.618 | 0.000 | 0.548 | 1.000 |
| 16 | RM341 | 2 | 3 | 130 | 170 | 0.593 | 0.586 | 0.023 | 0.540 | 0.962 |
| 17 | RM1385 | 2 | 3 | 130 | 170 | 0.634 | 0.502 | 0.037 | 0.423 | 0.926 |
| 18 | RM3685 | 2 | 3 | 100 | 130 | 0.436 | 0.649 | 0.000 | 0.576 | 1.000 |
| 19 | RM3857 | 2 | 2 | 110 | 137 | 0.690 | 0.428 | 0.080 | 0.336 | 0.815 |
| 20 | RM112 | 2 | 2 | 128 | 140 | 0.915 | 0.156 | 0.000 | 0.144 | 1.000 |
| 21 | RM250 | 2 | 3 | 140 | 170 | 0.618 | 0.507 | 0.000 | 0.422 | 1.000 |
| 22 | RM3894 | 3 | 2 | 190 | 200 | 0.995 | 0.011 | 0.000 | 0.011 | 1.000 |
| 23 | RM22 | 3 | 3 | 195 | 205 | 0.864 | 0.242 | 0.000 | 0.223 | 1.000 |
| 24 | RM569 | 3 | 3 | 145 | 175 | 0.853 | 0.254 | 0.006 | 0.226 | 0.975 |
| 25 | RM81 | 3 | 3 | 90 | 130 | 0.921 | 0.148 | 0.111 | 0.142 | 0.256 |
| 26 | RM3117 | 3 | 3 | 90 | 110 | 0.436 | 0.615 | 0.000 | 0.533 | 1.000 |
| 27 | RM5474 | 3 | 3 | 140 | 160 | 0.652 | 0.468 | 0.000 | 0.377 | 1.000 |
| 28 | RM338 | 3 | 3 | 180 | 200 | 0.948 | 0.099 | 0.000 | 0.095 | 1.000 |
| 29 | RM251 | 3 | 3 | 147 | 170 | 0.564 | 0.586 | 0.009 | 0.521 | 0.984 |
| 30 | RM16 | 3 | 3 | 171 | 230 | 0.808 | 0.322 | 0.000 | 0.290 | 1.000 |
| 31 | RM5626 | 3 | 3 | 190 | 200 | 0.372 | 0.664 | 0.000 | 0.590 | 1.000 |
| 32 | RM426 | 3 | 7 | 140 | 250 | 0.239 | 0.819 | 0.142 | 0.794 | 0.828 |
| 33 | RM1350 | 3 | 5 | 167 | 230 | 0.770 | 0.388 | 0.000 | 0.365 | 1.000 |
| 34 | RM514 | 3 | 2 | 260 | 270 | 0.859 | 0.243 | 0.000 | 0.213 | 1.000 |
| 35 | RM570 | 3 | 4 | 208 | 255 | 0.549 | 0.598 | 0.014 | 0.535 | 0.977 |
| 36 | RM16200 | 3 | 4 | 193 | 230 | 0.530 | 0.583 | 0.007 | 0.506 | 0.989 |
| 37 | RM85 | 3 | 4 | 80 | 110 | 0.418 | 0.711 | 0.049 | 0.663 | 0.931 |
| 38 | RM551 | 4 | 3 | 190 | 210 | 0.444 | 0.644 | 0.028 | 0.570 | 0.956 |
| 39 | RM335 | 4 | 2 | 105 | 145 | 0.833 | 0.278 | 0.000 | 0.239 | 1.000 |
| 40 | RM518 | 4 | 3 | 180 | 200 | 0.483 | 0.603 | 0.014 | 0.521 | 0.978 |
| 41 | RM185 | 4 | 2 | 190 | 200 | 0.690 | 0.428 | 0.008 | 0.337 | 0.981 |
| 42 | RM317 | 4 | 2 | 155 | 165 | 0.528 | 0.498 | 0.000 | 0.374 | 1.000 |
| 43 | RM5709 | 4 | 3 | 140 | 160 | 0.419 | 0.653 | 0.000 | 0.580 | 1.000 |
| 44 | RM8217 | 4 | 1 | 180 | 180 | 1.000 | 0.000 | 0.000 | 0.000 | NaN |
| 45 | RM5478 | 4 | 3 | 100 | 150 | 0.438 | 0.607 | 0.022 | 0.523 | 0.964 |
| 46 | RM507 | 5 | 2 | 250 | 260 | 0.686 | 0.431 | 0.000 | 0.338 | 1.000 |
| 47 | RM159 | 5 | 3 | 220 | 260 | 0.753 | 0.384 | 0.000 | 0.328 | 1.000 |
| 48 | RM169 | 5 | 4 | 170 | 200 | 0.300 | 0.736 | 0.022 | 0.687 | 0.971 |
| 49 | RM13 | 5 | 4 | 120 | 150 | 0.365 | 0.691 | 0.000 | 0.633 | 1.000 |
| 50 | RM146 | 5 | 2 | 340 | 350 | 0.649 | 0.456 | 0.000 | 0.352 | 1.000 |
| 51 | RM164 | 5 | 4 | 245 | 300 | 0.600 | 0.501 | 0.000 | 0.400 | 1.000 |
| 52 | RM39 | 5 | 2 | 115 | 125 | 0.757 | 0.368 | 0.000 | 0.300 | 1.000 |
| 53 | RM173 | 5 | 2 | 190 | 200 | 0.821 | 0.294 | 0.000 | 0.251 | 1.000 |
| 54 | RM188 | 5 | 1 | 210 | 210 | 1.000 | 0.000 | 0.000 | 0.000 | NaN |
| 55 | RM178 | 5 | 2 | 120 | 130 | 0.674 | 0.439 | 0.000 | 0.343 | 1.000 |
| 56 | RM274 | 5 | 2 | 150 | 160 | 0.514 | 0.500 | 0.043 | 0.375 | 0.915 |
| 57 | RM5784 | 5 | 3 | 110 | 130 | 0.355 | 0.666 | 0.000 | 0.592 | 1.000 |
| 58 | RM334 | 5 | 5 | 150 | 190 | 0.440 | 0.708 | 0.012 | 0.664 | 0.983 |
| 59 | RM3286 | 5 | 2 | 160 | 170 | 0.526 | 0.499 | 0.000 | 0.374 | 1.000 |
| 60 | RM133 | 6 | 1 | 190 | 230 | 0.929 | 0.132 | 0.000 | 0.123 | 1.000 |
| 61 | RM435 | 6 | 1 | 165 | 165 | 1.000 | 0.000 | 0.000 | 0.000 | NaN |
| 62 | RM190 | 6 | 3 | 125 | 145 | 0.495 | 0.577 | 0.016 | 0.487 | 0.972 |
| 63 | RM6917 | 6 | 3 | 140 | 170 | 0.517 | 0.613 | 0.000 | 0.543 | 1.000 |
| 64 | RM253 | 6 | 3 | 120 | 140 | 0.369 | 0.660 | 0.026 | 0.586 | 0.961 |
| 65 | RM2615 | 6 | 4 | 130 | 160 | 0.467 | 0.642 | 0.115 | 0.575 | 0.821 |
| 66 | RM5850 | 6 | 2 | 160 | 180 | 0.595 | 0.482 | 0.000 | 0.366 | 1.000 |
| 67 | RM1161 | 6 | 2 | 70 | 80 | 0.995 | 0.010 | 0.000 | 0.010 | 1.000 |
| 68 | RM6395 | 6 | 2 | 90 | 110 | 0.962 | 0.073 | 0.022 | 0.070 | 0.704 |
| 69 | RM400 | 6 | 5 | 190 | 320 | 0.294 | 0.756 | 0.010 | 0.712 | 0.987 |
| 70 | RM427 | 7 | 2 | 160 | 180 | 0.942 | 0.109 | 0.010 | 0.103 | 0.904 |
| 71 | RM214 | 7 | 4 | 110 | 140 | 0.608 | 0.577 | 0.026 | 0.536 | 0.954 |
| 72 | RM5481 | 7 | 3 | 140 | 160 | 0.760 | 0.390 | 0.000 | 0.354 | 1.000 |
| 73 | RM3691 | 7 | 3 | 130 | 150 | 0.438 | 0.650 | 0.045 | 0.577 | 0.930 |
| 74 | RM21693 | 7 | 2 | 390 | 410 | 0.867 | 0.231 | 0.000 | 0.204 | 1.000 |
| 75 | RM336 | 7 | 4 | 130 | 170 | 0.450 | 0.658 | 0.035 | 0.596 | 0.946 |
| 76 | RM21792 | 7 | 1 | 190 | 200 | 0.951 | 0.094 | 0.000 | 0.089 | 1.000 |
| 77 | RM6152 | 7 | 3 | 190 | 210 | 0.674 | 0.486 | 0.053 | 0.430 | 0.892 |
| 78 | RM10 | 7 | 3 | 150 | 170 | 0.488 | 0.597 | 0.000 | 0.514 | 1.000 |
| 79 | RM1335 | 7 | 3 | 160 | 180 | 0.475 | 0.578 | 0.000 | 0.487 | 1.000 |
| 80 | RM408 | 8 | 2 | 130 | 140 | 0.929 | 0.131 | 0.005 | 0.123 | 0.960 |
| 81 | RM1235 | 8 | 2 | 115 | 130 | 0.921 | 0.146 | 0.000 | 0.135 | 1.000 |
| 82 | RM38 | 8 | 2 | 230 | 250 | 0.900 | 0.180 | 0.009 | 0.164 | 0.952 |
| 83 | RM5647 | 8 | 4 | 100 | 140 | 0.444 | 0.674 | 0.037 | 0.617 | 0.945 |
| 84 | RM5428 | 8 | 2 | 220 | 450 | 0.776 | 0.347 | 0.000 | 0.287 | 1.000 |
| 85 | RM310 | 8 | 3 | 95 | 105 | 0.600 | 0.531 | 0.000 | 0.451 | 1.000 |
| 86 | RM210 | 8 | 3 | 140 | 160 | 0.595 | 0.561 | 0.006 | 0.497 | 0.989 |
| 87 | RM3452 | 8 | 4 | 180 | 220 | 0.315 | 0.735 | 0.048 | 0.686 | 0.935 |
| 88 | RM105 | 9 | 2 | 130 | 140 | 0.608 | 0.477 | 0.011 | 0.363 | 0.978 |
| 89 | RM24260 | 9 | 4 | 200 | 250 | 0.317 | 0.736 | 0.000 | 0.686 | 1.000 |
| 90 | RM409 | 9 | 2 | 85 | 95 | 0.591 | 0.483 | 0.000 | 0.367 | 1.000 |
| 91 | RM434 | 9 | 2 | 145 | 155 | 0.591 | 0.483 | 0.016 | 0.367 | 0.967 |
| 92 | RM257 | 9 | 4 | 130 | 160 | 0.647 | 0.519 | 0.023 | 0.465 | 0.956 |
| 93 | RM3533 | 9 | 3 | 120 | 140 | 0.401 | 0.639 | 0.011 | 0.562 | 0.983 |
| 94 | RM242 | 9 | 4 | 190 | 220 | 0.333 | 0.722 | 0.039 | 0.670 | 0.946 |
| 95 | RM6251 | 9 | 2 | 110 | 120 | 0.726 | 0.398 | 0.000 | 0.319 | 1.000 |
| 96 | RM160 | 9 | 4 | 90 | 130 | 0.730 | 0.435 | 0.011 | 0.400 | 0.975 |
| 97 | RM215 | 9 | 2 | 150 | 160 | 0.918 | 0.150 | 0.000 | 0.139 | 1.000 |
| 98 | RM6971 | 9 | 3 | 180 | 200 | 0.819 | 0.309 | 0.000 | 0.281 | 1.000 |
| 99 | RM3823 | 9 | 3 | 170 | 190 | 0.518 | 0.610 | 0.000 | 0.538 | 1.000 |
| 100 | RM245 | 9 | 2 | 140 | 150 | 0.506 | 0.500 | 0.012 | 0.375 | 0.976 |
| 101 | RM205 | 9 | 5 | 120 | 160 | 0.303 | 0.777 | 0.037 | 0.742 | 0.952 |
| 102 | RM3744 | 9 | 5 | 150 | 200 | 0.294 | 0.752 | 0.042 | 0.708 | 0.944 |
| 103 | RM6364 | 10 | 2 | 250 | 260 | 0.547 | 0.496 | 0.000 | 0.373 | 1.000 |
| 104 | RM8015 | 10 | 2 | 170 | 200 | 0.884 | 0.206 | 0.000 | 0.185 | 1.000 |
| 105 | RM8027 | 10 | 3 | 90 | 110 | 0.926 | 0.140 | 0.000 | 0.134 | 1.000 |
| 106 | RM4455 | 10 | 2 | 60 | 70 | 0.985 | 0.030 | 0.000 | 0.029 | 1.000 |
| 107 | RM467 | 10 | 2 | 200 | 220 | 0.947 | 0.100 | 0.000 | 0.095 | 1.000 |
| 108 | RM5304 | 10 | 1 | 340 | 340 | 1.000 | 0.000 | 0.000 | 0.000 | NaN |
| 109 | RM5392 | 10 | 2 | 270 | 300 | 0.583 | 0.486 | 0.000 | 0.368 | 1.000 |
| 110 | RM147 | 10 | 2 | 90 | 100 | 0.570 | 0.490 | 0.000 | 0.370 | 1.000 |
| 111 | RM25790 | 10 | 2 | 250 | 260 | 0.613 | 0.474 | 0.000 | 0.362 | 1.000 |
| 112 | RM590 | 10 | 2 | 130 | 140 | 0.808 | 0.311 | 0.000 | 0.262 | 1.000 |
| 113 | RM7203 | 11 | 2 | 95 | 105 | 0.911 | 0.161 | 0.017 | 0.148 | 0.894 |
| 114 | RM536 | 11 | 3 | 220 | 240 | 0.514 | 0.607 | 0.000 | 0.533 | 1.000 |
| 115 | RM202 | 11 | 4 | 160 | 190 | 0.407 | 0.678 | 0.032 | 0.616 | 0.953 |
| 116 | RM3428 | 11 | 3 | 140 | 160 | 0.451 | 0.627 | 0.016 | 0.550 | 0.974 |
| 117 | RM209 | 11 | 4 | 130 | 160 | 0.306 | 0.738 | 0.033 | 0.689 | 0.955 |
| 118 | RM254 | 11 | 2 | 165 | 175 | 0.561 | 0.492 | 0.000 | 0.371 | 1.000 |
| 119 | RM1233 | 11 | 3 | 160 | 180 | 0.409 | 0.640 | 0.013 | 0.563 | 0.981 |
| 120 | RM224 | 11 | 3 | 140 | 160 | 0.486 | 0.613 | 0.040 | 0.535 | 0.934 |
| 121 | sbq1 | 11 | 2 | 230 | 240 | 0.966 | 0.066 | 0.058 | 0.064 | 0.126 |
| 122 | sbq11 | 11 | 2 | 145 | 155 | 0.522 | 0.499 | 0.000 | 0.375 | 1.000 |
| 123 | K39512 | 11 | 2 | 90 | 110 | 0.781 | 0.342 | 0.000 | 0.283 | 1.000 |
| 124 | RM7443 | 11 | 2 | 140 | 150 | 0.683 | 0.433 | 0.044 | 0.339 | 0.900 |
| 125 | sbq33 | 11 | 2 | 250 | 260 | 0.624 | 0.469 | 0.000 | 0.359 | 1.000 |
| 126 | RM1880 | 12 | 2 | 100 | 130 | 0.967 | 0.063 | 0.000 | 0.061 | 1.000 |
| 127 | RM20A | 12 | 2 | 190 | 200 | 0.547 | 0.496 | 0.000 | 0.373 | 1.000 |
| 128 | RM247 | 12 | 4 | 130 | 180 | 0.478 | 0.632 | 0.017 | 0.562 | 0.973 |
| 129 | RM101 | 12 | 2 | 280 | 320 | 0.927 | 0.135 | 0.007 | 0.126 | 0.949 |
| 130 | RM1337 | 12 | 3 | 190 | 210 | 0.577 | 0.555 | 0.000 | 0.478 | 1.000 |
| 131 | RM5364 | 12 | 3 | 150 | 200 | 0.867 | 0.240 | 0.000 | 0.226 | 1.000 |
| 132 | RM28130 | 12 | 2 | 170 | 180 | 0.789 | 0.333 | 0.000 | 0.278 | 1.000 |
| 133 | RM7025 | 12 | 2 | 90 | 100 | 0.756 | 0.369 | 0.000 | 0.301 | 1.000 |
|  | Mean |  | 2.7594 |  |  | 0.642 | 0.443 | 0.013 | 0.383 | 0.972 |

**Supplementary Table 8.** Population structure group of rice genotypes based on inferred ancestry values.

| **Sl. No.** | **Genotypes** | **Inferred ancestry** | | **Structure Group** | **Response to Sheath blight** |
| --- | --- | --- | --- | --- | --- |
|  |  | **Q1** | **Q2** |  |  |
| 1 | Domsufod | 0.8070 | 0.1930 | SP1 | MS |
| 2 | Dular | 0.8440 | 0.1560 | SP1 | MS |
| 3 | Sadu Cha | 0.9950 | 0.0050 | SP1 | MS |
| 4 | Sanhuangzhan | 0.9280 | 0.0720 | SP1 | MS |
| 5 | Zhenshan 97 b | 0.9440 | 0.0560 | SP1 | S |
| 6 | Cypress | 0.5640 | 0.4360 | AD | MS |
| 7 | ARC 10376 | 0.0020 | 0.9980 | SP2 | MS |
| 8 | Black Gora | 0.0180 | 0.9820 | SP2 | MS |
| 9 | CTG 1516 | 0.0020 | 0.9980 | SP2 | MS |
| 10 | DD 62 | 0.0030 | 0.9970 | SP2 | MS |
| 11 | Dhala Shaiptta | 0.0200 | 0.9800 | SP2 | S |
| 12 | DJ 24 | 0.0120 | 0.9880 | SP2 | MS |
| 13 | BM 43 | 0.0020 | 0.9980 | SP2 | MS |
| 14 | BM 56 | 0.0010 | 0.9990 | SP2 | S |
| 15 | DM 59 | 0.0010 | 0.9990 | SP2 | MS |
| 16 | DV 123 | 0.0070 | 0.9930 | SP2 | MS |
| 17 | DV 85 | 0.0030 | 0.9970 | SP2 | MS |
| 18 | Ghor Bhai | 0.9920 | 0.0080 | SP1 | S |
| 19 | Jamir | 0.0030 | 0.9970 | SP2 | MS |
| 20 | Jhona 349 | 0.0020 | 0.9980 | SP2 | S |
| 21 | Kachilon | 0.0040 | 0.9960 | SP2 | MS |
| 22 | PTB 30 | 0.6750 | 0.3250 | AD | MS |
| 23 | T-26 | 0.0030 | 0.9970 | SP2 | MS |
| 24 | 99216 | 0.0040 | 0.9960 | SP2 | MS |
| 25 | ARC 10378 | 0.0050 | 0.9950 | SP2 | MS |
| 26 | Bowalia | 0.0360 | 0.9640 | SP2 | MS |
| 27 | NP 97 | 0.0090 | 0.9910 | SP2 | MR |
| 28 | Asahi | 0.9610 | 0.0390 | SP1 | S |
| 29 | Bachi boro | 0.0150 | 0.9850 | SP2 | MS |
| 30 | BR6 | 0.9950 | 0.0050 | SP1 | MS |
| 31 | Lara | 0.0080 | 0.9920 | SP2 | MS |
| 32 | Rata Boro | 0.2050 | 0.7950 | SP2 | MS |
| 33 | Tulsi Boro | 0.3820 | 0.6180 | AD | MS |
| 34 | Tupa | 0.6740 | 0.3260 | AD | MS |
| 35 | Gobir Sail | 0.3260 | 0.6740 | AD | MR |
| 36 | Dubhi Gora | 0.0020 | 0.9980 | SP2 | MS |
| 37 | Anjani | 0.6250 | 0.3750 | AD | MS |
| 38 | ARC 11205 | 0.0110 | 0.9890 | SP2 | MS |
| 39 | ARC 11600 | 0.0020 | 0.9980 | SP2 | MS |
| 40 | ARC 14855 | 0.0060 | 0.9940 | SP2 | MS |
| 41 | ARC 14965 | 0.0020 | 0.9980 | SP2 | MS |
| 42 | ARC 14969 | 0.0030 | 0.9970 | SP2 | MS |
| 43 | ARC 5959 | 0.0130 | 0.9870 | SP2 | MS |
| 44 | ARC 5960 | 0.0100 | 0.9900 | SP2 | MS |
| 45 | ARC 5977 | 0.0020 | 0.9980 | SP2 | MS |
| 46 | ARC 7098 | 0.0030 | 0.9970 | SP2 | MR |
| 47 | ARC 7325 | 0.0060 | 0.9940 | SP2 | MR |
| 48 | AUS 100 | 0.0490 | 0.9510 | SP2 | MS |
| 49 | AUS 125 | 0.0170 | 0.9830 | SP2 | MS |
| 50 | AUS 12 | 0.0070 | 0.9930 | SP2 | MS |
| 51 | AUS 130 | 0.0090 | 0.9910 | SP2 | MR |
| 52 | AUS 131 | 0.0020 | 0.9980 | SP2 | MS |
| 53 | DF 3 | 0.0320 | 0.9680 | SP2 | MS |
| 54 | AUS 169 | 0.1480 | 0.8520 | SP2 | MS |
| 55 | AUS 175 | 0.0040 | 0.9960 | SP2 | MS |
| 56 | AUS 180 | 0.2330 | 0.7670 | SP2 | MS |
| 57 | AUS 204 | 0.0030 | 0.9970 | SP2 | MS |
| 58 | AUS 209 | 0.0020 | 0.9980 | SP2 | MS |
| 59 | AUS 210 | 0.0010 | 0.9990 | SP2 | MS |
| 60 | AUS 267 | 0.0050 | 0.9950 | SP2 | MS |
| 61 | AUS 268 | 0.9460 | 0.0540 | SP1 | MS |
| 62 | AUS 273 | 0.0030 | 0.9970 | SP2 | MR |
| 63 | AUS 277 | 0.0020 | 0.9980 | SP2 | MS |
| 64 | AUS 283 | 0.2310 | 0.7690 | SP2 | MR |
| 65 | AUS 28 | 0.3460 | 0.6540 | AD | MR |
| 66 | AUS 314 | 0.0010 | 0.9990 | SP2 | MR |
| 67 | AUS 31 | 0.0020 | 0.9980 | SP2 | MR |
| 68 | AUS 335 | 0.0020 | 0.9980 | SP2 | MR |
| 69 | AUS 350 | 0.0030 | 0.9970 | SP2 | MR |
| 70 | AUS 362 | 0.0070 | 0.9930 | SP2 | MS |
| 71 | AUS 354 | 0.0040 | 0.9960 | SP2 | MR |
| 72 | AUS 382 | 0.0200 | 0.9800 | SP2 | MS |
| 73 | AUS 411 | 0.0520 | 0.9480 | SP2 | MS |
| 74 | AUS 385 | 0.0020 | 0.9980 | SP2 | MR |
| 75 | AUS 414 | 0.0190 | 0.9810 | SP2 | MS |
| 76 | AUS 415 | 0.0170 | 0.9830 | SP2 | MS |
| 77 | Hijli | 0.0050 | 0.9950 | SP2 | MS |
| 78 | AUS 420 | 0.0070 | 0.9930 | SP2 | MS |
| 79 | Kal Buri | 0.0040 | 0.9960 | SP2 | MR |
| 80 | Early Sutarsar 39 | 0.0180 | 0.9820 | SP2 | MS |
| 81 | AUS 462 | 0.0050 | 0.9950 | SP2 | MR |
| 82 | AUS 464 | 0.0020 | 0.9980 | SP2 | MS |
| 83 | AUS 46 | 0.0100 | 0.9900 | SP2 | MS |
| 84 | AUS 60 | 0.9830 | 0.0170 | SP1 | MR |
| 85 | AUS 62 | 0.0090 | 0.9910 | SP2 | MR |
| 86 | AUS 74 | 0.0030 | 0.9970 | SP2 | MS |
| 87 | AUS 77 | 0.0030 | 0.9970 | SP2 | MR |
| 88 | AUS KHUSI | 0.0030 | 0.9970 | SP2 | MS |
| 89 | AUSMERI | 0.0050 | 0.9950 | SP2 | MR |
| 90 | AUS PADDY(BLACK) | 0.0020 | 0.9980 | SP2 | MR |
| 91 | Bausphr | 0.0040 | 0.9960 | SP2 | MS |
| 92 | Bircona | 0.1450 | 0.8550 | SP2 | MS |
| 93 | Brown Gora s b 92 | 0.0050 | 0.9950 | SP2 | MR |
| 94 | Chandra Kana | 0.9850 | 0.0150 | SP1 | MS |
| 95 | Chandra Mukhi | 0.3070 | 0.6930 | AD | MS |
| 96 | CN 2- 175-5-31 | 0.0080 | 0.9920 | SP2 | MS |
| 97 | CTG 250 | 0.0520 | 0.9480 | SP2 | MS |
| 98 | DJ 29 | 0.0200 | 0.9800 | SP2 | MR |
| 99 | Dubhi Gora | 0.1380 | 0.8620 | SP2 | MS |
| 100 | Gohama Bhadri | 0.2420 | 0.7580 | SP2 | MR |
| 101 | Jagle Boro | 0.1520 | 0.8480 | SP2 | MR |
| 102 | Kada 176-12 | 0.9750 | 0.0250 | SP1 | MR |
| 103 | Kalasu | 0.0140 | 0.9860 | SP2 | MR |
| 104 | Kali AUS | 0.0290 | 0.9710 | SP2 | MR |
| 105 | M 136-20 | 0.0670 | 0.9330 | SP2 | MR |
| 106 | Parbat jIra | 0.3290 | 0.6710 | AD | MR |
| 107 | White Dubhi | 0.9970 | 0.0030 | SP1 | MS |
| 108 | AUS 152 | 0.0240 | 0.9760 | SP2 | MS |
| 109 | AUS 154 | 0.1570 | 0.8430 | SP2 | MR |
| 110 | AUS 321 | 0.2210 | 0.7790 | SP2 | MS |
| 111 | AUS 366 | 0.2610 | 0.7390 | SP2 | MS |
| 112 | AUS 391 | 0.9890 | 0.0110 | SP1 | MS |
| 113 | Dhingha | 0.0170 | 0.9830 | SP2 | MS |
| 114 | MTU 18 | 0.3370 | 0.6630 | AD | MS |
| 115 | Raj Munlo | 0.2270 | 0.7730 | SP2 | MR |
| 116 | Nagri | 0.1180 | 0.8820 | SP2 | MS |
| 117 | Boraya | 0.0310 | 0.9690 | SP2 | MS |
| 118 | AUS 298 | 0.0310 | 0.9690 | SP2 | MS |
| 119 | CO(R)-48 | 0.7700 | 0.2300 | SP1 | MS |
| 120 | CO(R)-51 | 0.7470 | 0.2530 | SP1 | MS |
| 121 | ASD-16 | 0.7390 | 0.2610 | SP1 | MS |
| 122 | GEB-24 | 0.9890 | 0.0110 | SP1 | MS |
| 123 | Anna (R)-4 | 0.9940 | 0.0060 | SP1 | MS |
| 124 | BPT 5204 | 0.9950 | 0.0050 | SP1 | MR |
| 125 | IR-36 | 0.9300 | 0.0700 | SP1 | MS |
| 126 | Kodaikulathan | 0.9760 | 0.0240 | SP1 | MR |
| 127 | Mattai Kar | 0.9810 | 0.0190 | SP1 | S |
| 128 | Kauni | 0.9980 | 0.0020 | SP1 | MS |
| 129 | Sivappu Chithirai Kar | 0.9920 | 0.0080 | SP1 | MR |
| 130 | Soora Kuruvai | 0.9980 | 0.0020 | SP1 | MR |
| 131 | Swarna | 0.9990 | 0.0010 | SP1 | MR |
| 132 | ADT-37 | 0.9750 | 0.0250 | SP1 | MR |
| 133 | ADT-38 | 0.8820 | 0.1180 | SP1 | MS |
| 134 | ADT-41 | 0.9980 | 0.0020 | SP1 | MS |
| 135 | ADT-42 | 0.9950 | 0.0050 | SP1 | MS |
| 136 | ADT-43 | 0.9970 | 0.0030 | SP1 | MS |
| 137 | ADT-45 | 0.9940 | 0.0060 | SP1 | MR |
| 138 | ADT-48 | 0.9690 | 0.0310 | SP1 | S |
| 139 | ADT-51 | 0.9930 | 0.0070 | SP1 | MR |
| 140 | TKM-12 | 0.9910 | 0.0090 | SP1 | MR |
| 141 | TKM-13 | 0.9960 | 0.0040 | SP1 | MR |
| 142 | CR Dhan 311 | 0.9820 | 0.0180 | SP1 | MR |
| 143 | CR Dhan 401 | 0.7370 | 0.2630 | SP1 | MR |
| 144 | CR Dhan 404 | 0.8990 | 0.1010 | SP1 | MR |
| 145 | CR Dhan 405 | 0.9320 | 0.0680 | SP1 | MS |
| 146 | CR Dhan 501 | 0.9570 | 0.0430 | SP1 | MR |
| 147 | CR Dhan 505 | 0.9920 | 0.0080 | SP1 | MR |
| 148 | CR Dhan 701 | 0.9980 | 0.0020 | SP1 | MS |
| 149 | CR Dhan 801 | 0.9220 | 0.0780 | SP1 | MS |
| 150 | CR Dhan 902 | 0.9980 | 0.0020 | SP1 | MR |
| 151 | CR Dhan 907 | 0.9980 | 0.0020 | SP1 | MS |
| 152 | Sarjoo 52 | 0.9970 | 0.0030 | SP1 | MS |
| 153 | HUR-43 | 0.9980 | 0.0020 | SP1 | MS |
| 154 | IC 277237 | 0.2550 | 0.7450 | SP2 | MS |
| 155 | IC 256613 | 0.4510 | 0.5490 | AD | MR |
| 156 | IC 256616 | 0.9960 | 0.0040 | SP1 | MR |
| 157 | IC 260917 | 0.9780 | 0.0220 | SP1 | MR |
| 158 | IC 264141 | 0.9980 | 0.0020 | SP1 | MS |
| 159 | IC 274377 | 0.9970 | 0.0030 | SP1 | S |
| 160 | IC 274408 | 0.9850 | 0.0150 | SP1 | MR |
| 161 | IC 277248 | 0.9940 | 0.0060 | SP1 | MR |
| 162 | IC 277261 | 0.9380 | 0.0620 | SP1 | MR |
| 163 | IC 277274 | 0.5170 | 0.4830 | AD | MR |
| 164 | IC 277284 | 0.9950 | 0.0050 | SP1 | MR |
| 165 | IC 277290 | 0.0180 | 0.9820 | SP2 | MS |
| 166 | IC 277332 | 0.5170 | 0.4830 | AD | MS |
| 167 | IC 279355 | 0.2750 | 0.7250 | SP2 | MR |
| 168 | IC 280478 | 0.0370 | 0.9630 | SP2 | MS |
| 169 | IC 280504 | 0.8300 | 0.1700 | SP1 | MS |
| 170 | IC 280564 | 0.9960 | 0.0040 | SP1 | MR |
| 171 | IC 281508 | 0.0670 | 0.9330 | SP2 | MS |
| 172 | IC 283139 | 0.9900 | 0.0100 | SP1 | MR |
| 173 | IC 282460 | 0.8950 | 0.1050 | SP1 | MS |
| 174 | IC 282812 | 0.9640 | 0.0360 | SP1 | S |
| 175 | IC 282815 | 0.2030 | 0.7970 | SP2 | MR |
| 176 | IC 283204 | 0.3880 | 0.6120 | AD | MR |
| 177 | IC 277267 | 0.9950 | 0.0050 | SP1 | MR |
| 178 | IC 277275 | 0.9950 | 0.0050 | SP1 | MS |
| 179 | IC 283206 | 0.3640 | 0.6360 | AD | MR |
| 180 | CO 39 | 0.9580 | 0.0420 | SP1 | MR |
| 181 | AUS MURALI | 0.0060 | 0.9940 | SP2 | MR |
| 182 | BCULARN | 0.0060 | 0.9940 | SP2 | MR |
| 183 | BORO BLACK | 0.0190 | 0.9810 | SP2 | MR |
| 184 | BORO | 0.1790 | 0.8210 | SP2 | MR |
| 185 | BOWALIA | 0.0020 | 0.9980 | SP2 | MR |
| 186 | CHAMKA | 0.0320 | 0.9680 | SP2 | MS |
| 187 | DA 12 | 0.0030 | 0.9970 | SP2 | MR |
| 188 | Tetep | 0.9980 | 0.0020 | SP1 | MR |
| 189 | Pusa Basmati-1 | 0.7350 | 0.2650 | SP1 | S |
| 190 | Jasmine-85 | 0.9620 | 0.0380 | SP1 | MS |
| 191 | Teqing | 0.9880 | 0.0120 | SP1 | MS |
| 192 | Tapaswini | 0.9920 | 0.0080 | SP1 | S |

SP1- sub population 1, SP2- sub population 2, AD- admixture, MR- moderately resistant, MS- moderately susceptible, S- Susceptible

**Supplementary Table 9.** Association of marker alleles with disease-related traits of sheath blight and other morphological traits in rice detected both in GLM and MLM (Q+K) analyses in a shortlisted panel population of 192 genotypes

| **Sl. No.** | **Trait** | **Marker** | **Chr. No.** | **GLM** | | | | **MLM** | | | |
| --- | --- | --- | --- | --- | --- | --- | --- | --- | --- | --- | --- |
|  |  |  |  | ***F* value** | ***p* value** | ***q* value** | ***R^2^*** | ***F* value** | ***p* value** | ***q* value** | ***R^2^*** |
| **Season 1** | | | | | | | | | | | |
| 1 | 14P | RM13B | 5 | 6.96462 | 0.01018 | 0.011646 | 0.0882 | - | - | - | - |
| 2 | 14P | RM242A | 9 | 10.27833 | 0.00158 | 0.002329 | 0.05132 | - | - | - | - |
| 3 | 14P | RM250C | 2 | 11.99994 | 0.000662 | 0.001245 | 0.06122 | 13.01824 | 0.001737 | 0.004189 | 0.06835 |
| 4 | 14P | RM257C | 9 | 12.24365 | 0.000594 | 0.001164 | 0.06645 | - | - | - | - |
| 5 | 14P | RM310B | 8 | 10.06513 | 0.00181 | 0.002563 | 0.05816 | - | - | - | - |
| 6 | 14P | RM335A | 4 | 11.95528 | 0.000712 | 0.001322 | 0.07474 | 9.30062 | 6.73E-05 | 0.000394 | 0.05728 |
| 7 | 14P | RM3482B | 1 | 13.82945 | 0.000279 | 0.000655 | 0.08191 | 7.83971 | 9.39E-05 | 0.000481 | 0.05491 |
| 8 | 14P | RM514A | 3 | 14.31583 | 0.000212 | 0.000551 | 0.07562 | - | - | - | - |
| 9 | 21P | RM13D | 5 | 5.9343 | 0.01732 | 0.018483 | 0.07614 | - | - | - | - |
| 10 | 21P | RM250C | 2 | 12.51478 | 0.000511 | 0.001044 | 0.06368 | 13.13474 | 0.019725 | 0.028883 | 0.06829 |
| 11 | 21P | RM335A | 4 | 10.28745 | 0.00164 | 0.002345 | 0.06499 | 10.85025 | 0.00017 | 0.000633 | 0.06612 |
| 12 | 21P | RM3482B | 1 | 14.33377 | 0.000218 | 0.000548 | 0.08465 | 8.50115 | 1.28E-05 | 0.000105 | 0.06368 |
| 13 | 21P | RM400B | 6 | 5.65578 | 0.0194 | 0.02025 | 0.05619 | 6.86485 | 0.035909 | 0.047492 | 0.06556 |
| 14 | 21P | sbq1A | 11 | 11.13592 | 0.00102 | 0.001716 | 0.05592 | - | - | - | - |
| 15 | 7P | RM1339A | 1 | 11.10825 | 0.00104 | 0.001709 | 0.05782 | - | - | - | - |
| 16 | 7P | RM133A | 6 | 10.64772 | 0.00136 | 0.002091 | 0.06506 | - | - | - | - |
| 17 | 7P | RM257C | 9 | 9.31356 | 0.00264 | 0.003401 | 0.05137 | - | - | - | - |
| 18 | 7P | RM3428A | 11 | 13.01085 | 0.000399 | 0.000865 | 0.06638 | - | - | - | - |
| 19 | 7P | RM3482B | 1 | 9.11958 | 0.00296 | 0.003618 | 0.05557 | - | - | - | - |
| 20 | 7P | RM5784B | 5 | 5.71391 | 0.01861 | 0.019568 | 0.05161 | - | - | - | - |
| 21 | AUDPC | RM13B | 5 | 6.44542 | 0.01329 | 0.014398 | 0.08216 | - | - | - | - |
| 22 | AUDPC | RM250C | 2 | 14.25102 | 0.000216 | 0.000551 | 0.07188 | 15.10104 | 0.004224 | 0.008659 | 0.07819 |
| 23 | AUDPC | RM257C | 9 | 11.29415 | 0.000958 | 0.00167 | 0.06162 | - | - | - | - |
| 24 | AUDPC | RM335A | 4 | 9.24296 | 0.0028 | 0.003482 | 0.05878 | - | - | - | - |
| 25 | AUDPC | RM3482B | 1 | 17.50026 | 4.79E-05 | 0.000159 | 0.10145 | 10.21298 | 1.82E-05 | 0.000124 | 0.07471 |
| 26 | AUDPC | RM514A | 3 | 11.24889 | 0.000977 | 0.001683 | 0.0604 | - | - | - | - |
| 27 | DH | K39512A | 11 | 14.58185 | 0.000192 | 0.000509 | 0.08449 | - | - | - | - |
| 28 | DH | RM11229C | 1 | 12.73249 | 0.000502 | 0.00104 | 0.08797 | - | - | - | - |
| 29 | DH | RM1232A | 1 | 26.78248 | 6.16E-07 | 5.18E-06 | 0.13207 | - | - | - | - |
| 30 | DH | RM1233B | 11 | 9.29585 | 0.00269 | 0.003435 | 0.05557 | - | - | - | - |
| 31 | DH | RM335A | 4 | - | - | - | - | 19.1746 | 0.146179 | 0.171238 | 0.10897 |
| 32 | DH | RM1335A | 7 | 7.09252 | 0.00882 | 0.010171 | 0.0567 | - | - | - | - |
| 33 | DH | RM133A | 6 | 10.93417 | 0.00118 | 0.001854 | 0.0667 | - | - | - | - |
| 34 | DH | RM1385A | 2 | 23.28459 | 2.91E-06 | 1.54E-05 | 0.11179 | - | - | - | - |
| 35 | DH | RM13C | 5 | 4.2108 | 0.0438 | 0.0438 | 0.05525 | 3.08158 | 0.037436 | 0.047965 | 0.07118 |
| 36 | DH | RM147A | 10 | 21.65847 | 8.13E-06 | 3.32E-05 | 0.14668 | - | - | - | - |
| 37 | DH | RM14C | 1 | 6.39767 | 0.01313 | 0.014333 | 0.06502 | - | - | - | - |
| 38 | DH | RM159A | 5 | 27.14281 | 4.94E-07 | 4.42E-06 | 0.12616 | - | - | - | - |
| 39 | DH | RM16200B | 3 | 17.97509 | 3.92E-05 | 0.000134 | 0.1083 | - | - | - | - |
| 40 | DH | RM164A | 5 | 25.1376 | 1.26E-06 | 8.16E-06 | 0.12077 | - | - | - | - |
| 41 | DH | RM169A | 5 | 13.14696 | 0.000373 | 0.000821 | 0.06703 | - | - | - | - |
| 42 | DH | RM173A | 5 | 9.20675 | 0.00281 | 0.003464 | 0.05441 | - | - | - | - |
| 43 | DH | RM205B | 9 | 28.27956 | 2.99E-07 | 2.85E-06 | 0.13198 | - | - | - | - |
| 44 | DH | RM209D | 11 | 18.46134 | 2.85E-05 | 0.000104 | 0.09397 | - | - | - | - |
| 45 | DH | RM20AA | 12 | 23.60162 | 2.7E-06 | 1.54E-05 | 0.12318 | - | - | - | - |
| 46 | DH | RM210A | 8 | 12.93917 | 0.000424 | 0.000892 | 0.07231 | - | - | - | - |
| 47 | DH | RM242A | 9 | 13.81464 | 0.000265 | 0.000632 | 0.06778 | - | - | - | - |
| 48 | DH | RM253A | 6 | 11.54404 | 0.000829 | 0.0015 | 0.05756 | - | - | - | - |
| 49 | DH | RM254A | 11 | 9.35853 | 0.00278 | 0.003487 | 0.07711 | - | - | - | - |
| 50 | DH | RM257A | 9 | 50.2791 | 3.31E-11 | 7.9E-10 | 0.2262 | - | - | - | - |
| 51 | DH | RM2615C | 6 | 42.35796 | 7.31E-10 | 1.16E-08 | 0.19049 | - | - | - | - |
| 52 | DH | RM274A | 5 | 48.18186 | 1.39E-10 | 2.83E-09 | 0.25879 | - | - | - | - |
| 53 | DH | RM306B | 1 | 22.77566 | 8.34E-06 | 3.31E-05 | 0.226 | - | - | - | - |
| 54 | DH | RM3286A | 5 | 24.07742 | 3.93E-06 | 1.81E-05 | 0.20565 | 7.15973 | 8.53E-07 | 8.75E-06 | 0.0747 |
| 55 | DH | RM334A | 5 | 17.09873 | 8.61E-05 | 0.000256 | 0.1743 | 8.3229 | 0.008138 | 0.015166 | 0.09527 |
| 56 | DH | RM341D | 2 | 35.36891 | 1.45E-08 | 1.72E-07 | 0.16813 | - | - | - | - |
| 57 | DH | RM3428A | 11 | 51.20151 | 1.95E-11 | 5.56E-10 | 0.21862 | - | - | - | - |
| 58 | DH | RM3482A | 1 | 21.61369 | 7.09E-06 | 2.98E-05 | 0.12238 | - | - | - | - |
| 59 | DH | RM3685A | 2 | 64.11627 | 1.5E-13 | 1.08E-11 | 0.26591 | - | - | - | - |
| 60 | DH | RM38A | 8 | 8.43405 | 0.00443 | 0.005323 | 0.06945 | - | - | - | - |
| 61 | DH | RM39A | 5 | 12.13742 | 0.000648 | 0.001236 | 0.07486 | - | - | - | - |
| 62 | DH | RM409A | 9 | 78.02134 | 8E-16 | 1.14E-13 | 0.29777 | - | - | - | - |
| 63 | DH | RM424A | 2 | 10.66621 | 0.00144 | 0.002168 | 0.08488 | - | - | - | - |
| 64 | DH | RM434B | 9 | 25.14988 | 1.24E-06 | 8.43E-06 | 0.11968 | - | - | - | - |
| 65 | DH | RM5364A | 12 | 13.28933 | 0.000359 | 0.000814 | 0.07538 | - | - | - | - |
| 66 | DH | RM536B | 11 | 22.77807 | 3.74E-06 | 1.84E-05 | 0.11178 | - | - | - | - |
| 67 | DH | RM5428A | 8 | 10.10294 | 0.00208 | 0.002806 | 0.10851 | - | - | - | - |
| 68 | DH | RM5474A | 3 | 22.75792 | 3.8E-06 | 1.81E-05 | 0.1128 | - | - | - | - |
| 69 | DH | RM5478B | 4 | 25.90308 | 8.9E-07 | 6.7E-06 | 0.12459 | - | - | - | - |
| 70 | DH | RM551C | 4 | 39.99856 | 2.05E-09 | 2.67E-08 | 0.18604 | - | - | - | - |
| 71 | DH | RM5626C | 3 | 19.03101 | 2.35E-05 | 9.06E-05 | 0.10999 | - | - | - | - |
| 72 | DH | RM5784C | 5 | 9.77356 | 0.00229 | 0.003004 | 0.08516 | - | - | - | - |
| 73 | DH | RM6251A | 9 | 53.57967 | 7.03E-12 | 2.51E-10 | 0.22179 | - | - | - | - |
| 74 | DH | RM6917B | 6 | 28.26568 | 8.08E-07 | 6.42E-06 | 0.24522 | 6.903 | 0.000116 | 0.000478 | 0.12319 |
| 75 | DH | RM85B | 3 | 23.47725 | 2.71E-06 | 1.49E-05 | 0.11482 | - | - | - | - |
| 76 | GY | RM101B | 12 | 15.5712 | 0.000124 | 0.000356 | 0.09882 | 8.46003 | 0 | 0 | 0.06976 |
| 77 | GY | RM1350A | 3 | 8.03953 | 0.00572 | 0.006705 | 0.08641 | 5.04055 | 0.001053 | 0.00288 | 0.06239 |
| 78 | GY | RM1385C | 2 | 9.81198 | 0.00202 | 0.002751 | 0.05037 | - | - | - | - |
| 79 | GY | RM13B | 5 | 11.63371 | 0.00107 | 0.001739 | 0.1391 | 4.55985 | 0.000953 | 0.00279 | 0.06284 |
| 80 | GY | RM160A | 9 | 10.98503 | 0.00111 | 0.001783 | 0.05663 | - | - | - | - |
| 81 | GY | RM22B | 3 | 11.55752 | 0.000837 | 0.001477 | 0.06229 | - | - | - | - |
| 82 | GY | RM3857A | 2 | 13.66281 | 0.000361 | 0.000806 | 0.12236 | 8.31822 | 0.033556 | 0.045859 | 0.09198 |
| 83 | GY | RM5428A | 8 | 11.63325 | 0.001 | 0.001702 | 0.12293 | 3.62041 | 3.63E-07 | 7.44E-06 | 0.05438 |
| 84 | GY | RM5626C | 3 | 10.35322 | 0.00158 | 0.002306 | 0.06299 | 8.9505 | 0.000447 | 0.001409 | 0.0568 |
| 85 | GY | RM570C | 3 | 18.57605 | 3.06E-05 | 0.000107 | 0.11714 | - | - | - | - |
| 86 | GY | RM6292B | 1 | 10.55654 | 0.0014 | 0.00213 | 0.05912 | - | - | - | - |
| 87 | GY | RM6917B | 6 | 5.88499 | 0.01734 | 0.018368 | 0.06336 | 4.05162 | 0.010948 | 0.019516 | 0.05124 |
| 88 | GY | RM7443B | 11 | 12.24431 | 0.000587 | 0.001167 | 0.06336 | - | - | - | - |
| 89 | GY | RM8015A | 10 | 10.32789 | 0.00162 | 0.00234 | 0.06692 | - | - | - | - |
| 90 | MP | RM1235A | 8 | 9.25898 | 0.00273 | 0.003455 | 0.05406 | - | - | - | - |
| 91 | MP | RM13B | 5 | 5.56948 | 0.02099 | 0.021594 | 0.0718 | - | - | - | - |
| 92 | MP | RM257C | 9 | 11.14535 | 0.00103 | 0.001713 | 0.06086 | - | - | - | - |
| 93 | MP | RM274B | 5 | 7.979 | 0.00543 | 0.006417 | 0.05466 | - | - | - | - |
| 94 | MP | RM306A | 1 | 5.18856 | 0.02547 | 0.025831 | 0.06237 | - | - | - | - |
| 95 | MP | RM3857A | 2 | 6.62372 | 0.01156 | 0.012915 | 0.06331 | - | - | - | - |
| 96 | MP | RM5392A | 10 | 8.06622 | 0.00541 | 0.006447 | 0.07072 | 9.44232 | 0.002272 | 0.005176 | 0.07797 |
| 97 | MP | RM5428A | 8 | 5.13702 | 0.02602 | 0.026203 | 0.05828 | 8.44186 | 0.001215 | 0.003114 | 0.08074 |
| 98 | MP | RM5647D | 8 | 21.43172 | 6.89E-06 | 3.08E-05 | 0.10382 | - | - | - | - |
| 99 | MP | RM570A | 3 | 11.97017 | 0.000717 | 0.001314 | 0.07877 | - | - | - | - |
| 100 | MP | RM8015A | 10 | 23.25649 | 3.57E-06 | 1.82E-05 | 0.13905 | 13.26222 | 5.77E-07 | 7.88E-06 | 0.1041 |
| 101 | NT | RM13B | 5 | 6.54986 | 0.01259 | 0.013849 | 0.08338 | - | - | - | - |
| 102 | NT | RM254A | 11 | - | - | - | - | 6.83547 | 0.024712 | 0.034937 | 0.05181 |
| 103 | NT | RM335A | 4 | - | - | - | - | 11.29069 | 0.016793 | 0.026482 | 0.07554 |
| 104 | NT | RM16200B | 3 | 13.83417 | 0.000283 | 0.000652 | 0.08548 | - | - | - | - |
| 105 | NT | RM16B | 3 | 17.00837 | 6.65E-05 | 0.000216 | 0.11729 | 12.30536 | 0.000182 | 0.000622 | 0.08878 |
| 106 | NT | RM250C | 2 | 14.0581 | 0.000237 | 0.000575 | 0.07098 | 14.71263 | 0.000113 | 0.000516 | 0.08017 |
| 107 | NT | RM424C | 2 | 6.79932 | 0.01033 | 0.011724 | 0.05582 | - | - | - | - |
| 108 | NT | RM5392A | 10 | 6.4908 | 0.01228 | 0.013613 | 0.0577 | - | - | - | - |
| 109 | NT | RM6917A | 6 | 6.09789 | 0.01549 | 0.016655 | 0.0655 | 2.99368 | 0.018651 | 0.028322 | 0.05387 |
| 110 | PH | RM6917C | 6 | - | - | - | - | 6.08101 | 0.369973 | 0.388946 | 0.06632 |
| 111 | PH | RM5364A | 12 | - | - | - | - | 10.17117 | 0.38241 | 0.39197 | 0.05483 |
| 112 | PH | RM1235C | 8 | 15.09425 | 0.000149 | 0.000417 | 0.08523 | - | - | - | - |
| 113 | PH | RM1350C | 3 | 10.29442 | 0.00188 | 0.002636 | 0.10803 | - | - | - | - |
| 114 | PH | RM13D | 5 | 19.8357 | 3.03E-05 | 0.000108 | 0.21599 | - | - | - | - |
| 115 | PH | RM16200D | 3 | 8.93239 | 0.00328 | 0.003975 | 0.05692 | - | - | - | - |
| 116 | PH | RM164A | 5 | 10.58965 | 0.00135 | 0.002098 | 0.0547 | - | - | - | - |
| 117 | PH | RM205D | 9 | 14.52116 | 0.000188 | 0.000508 | 0.07242 | - | - | - | - |
| 118 | PH | RM20AB | 12 | 9.69865 | 0.00217 | 0.002873 | 0.05458 | - | - | - | - |
| 119 | PH | RM21792B | 7 | 9.87532 | 0.00196 | 0.002721 | 0.05174 | - | - | - | - |
| 120 | PH | RM224C | 11 | 9.72018 | 0.00214 | 0.00286 | 0.05379 | - | - | - | - |
| 121 | PH | RM242A | 9 | 21.39775 | 6.89E-06 | 2.99E-05 | 0.10122 | - | - | - | - |
| 122 | PH | RM257A | 9 | 56.67858 | 2.76E-12 | 1.32E-10 | 0.24785 | - | - | - | - |
| 123 | PH | RM2615A | 6 | 15.5699 | 0.000114 | 0.000332 | 0.07961 | - | - | - | - |
| 124 | PH | RM274A | 5 | 43.11945 | 9.67E-10 | 1.38E-08 | 0.23807 | - | - | - | - |
| 125 | PH | RM306A | 1 | 9.79161 | 0.00247 | 0.003211 | 0.11153 | - | - | - | - |
| 126 | PH | RM310B | 8 | 16.58192 | 7.25E-05 | 0.000225 | 0.09234 | - | - | - | - |
| 127 | PH | RM3117C | 3 | 12.50289 | 0.000544 | 0.001095 | 0.07839 | - | - | - | - |
| 128 | PH | RM335A | 4 | 18.83685 | 2.62E-05 | 9.88E-05 | 0.11291 | - | - | - | - |
| 129 | PH | RM341A | 2 | 29.58634 | 1.78E-07 | 1.82E-06 | 0.14462 | - | - | - | - |
| 130 | PH | RM3482A | 1 | 16.79023 | 6.71E-05 | 0.000213 | 0.09774 | - | - | - | - |
| 131 | PH | RM3685A | 2 | 25.48034 | 1.1E-06 | 7.89E-06 | 0.12584 | - | - | - | - |
| 132 | PH | RM3744B | 9 | 10.38985 | 0.0015 | 0.002234 | 0.05264 | - | - | - | - |
| 133 | PH | RM3857A | 2 | 5.21124 | 0.0246 | 0.025127 | 0.05049 | - | - | - | - |
| 134 | PH | RM514A | 3 | 14.09941 | 0.000236 | 0.000581 | 0.07456 | - | - | - | - |
| 135 | PH | RM5474B | 3 | 11.56423 | 0.000829 | 0.001482 | 0.06068 | - | - | - | - |
| 136 | PH | RM551C | 4 | 24.0247 | 2.16E-06 | 1.29E-05 | 0.12071 | - | - | - | - |
| 137 | PH | RM5529B | 2 | 15.27357 | 0.00015 | 0.000414 | 0.10735 | - | - | - | - |
| 138 | PH | RM5709A | 4 | 13.1703 | 0.000403 | 0.000861 | 0.08949 | - | - | - | - |
| 139 | PH | RM5784A | 5 | 16.9526 | 7.65E-05 | 0.000233 | 0.13901 | - | - | - | - |
| 140 | PH | RM6251A | 9 | 31.31818 | 7.66E-08 | 8.42E-07 | 0.1428 | - | - | - | - |
| 141 | PH | RM85D | 3 | 45.73095 | 1.81E-10 | 3.23E-09 | 0.2017 | - | - | - | - |
| 142 | PL | RM1350C | 3 | 7.41553 | 0.00785 | 0.009126 | 0.08024 | - | - | - | - |
| 143 | PL | RM13D | 5 | 5.62377 | 0.0204 | 0.021139 | 0.07245 | 3.57013 | 0.012978 | 0.022171 | 0.14019 |
| 144 | PL | RM14C | 1 | 6.66717 | 0.0114 | 0.012836 | 0.06757 | 7.56623 | 0.055292 | 0.066676 | 0.06373 |
| 145 | PL | RM16C | 3 | 12.37219 | 0.000604 | 0.001167 | 0.08814 | 12.63184 | 0.015295 | 0.025084 | 0.08307 |
| 146 | PL | RM250C | 2 | 9.85149 | 0.00198 | 0.002723 | 0.05082 | - | - | - | - |
| 147 | PL | RM3117C | 3 | 10.97466 | 0.00116 | 0.001843 | 0.06947 | 6.43451 | 0.044758 | 0.055609 | 0.07198 |
| 148 | PL | sbq1A | 11 | 24.10266 | 1.98E-06 | 1.23E-05 | 0.11364 | - | - | - | - |
| 149 | PL | RM190A | 6 | - | - | - | - | 11.06811 | 0.003455 | 0.007456 | 0.0578 |
| 150 | PL | RM5364A | 12 | - | - | - | - | 11.23968 | 0.005298 | 0.010344 | 0.06093 |
| **Season 2** | | | | | | | | | | | |
| 151 | 14P | RM1350B | 3 | 5.64749 | 0.01973 | 0.021112 | 0.0623 | - | - | - | - |
| 152 | 14P | RM13B | 5 | 9.65611 | 0.0027 | 0.004014 | 0.11825 | 6.28116 | 0.000258 | 0.001587 | 0.06741 |
| 153 | 14P | RM160A | 9 | 14.17214 | 2.25E-04 | 0.000699 | 0.07188 | - | - | - | - |
| 154 | 14P | RM16B | 3 | 20.79366 | 1.18E-05 | 7.63E-05 | 0.13975 | 9.21107 | 0.000195 | 0.001419 | 0.07491 |
| 155 | 14P | RM178A | 5 | 5.44078 | 0.02198 | 0.023079 | 0.05886 | - | - | - | - |
| 156 | 14P | RM22B | 3 | 14.94805 | 1.56E-04 | 0.000534 | 0.07911 | - | - | - | - |
| 157 | 14P | RM306B | 1 | 12.98454 | 5.51E-04 | 0.001341 | 0.14271 | 11.06023 | 0.000154 | 0.001236 | 0.13015 |
| 158 | 14P | RM3452D | 8 | 22.84428 | 3.58E-06 | 2.87E-05 | 0.11044 | - | - | - | - |
| 159 | 14P | RM3825D | 1 | 8.93268 | 0.00334 | 0.004637 | 0.06338 | - | - | - | - |
| 160 | 14P | RM5428A | 8 | 8.60823 | 0.00433 | 0.005773 | 0.09397 | - | - | - | - |
| 161 | 14P | RM570C | 3 | 16.44222 | 8.29E-05 | 0.000357 | 0.1051 | - | - | - | - |
| 162 | 14P | RM6292C | 1 | 10.45084 | 0.00148 | 0.002617 | 0.05856 | - | - | - | - |
| 163 | 14P | RM6971C | 9 | 9.2953 | 0.00269 | 0.004035 | 0.05556 | - | - | - | - |
| 164 | 21P | RM1216A | 1 | 15.85199 | 1.01E-04 | 0.000413 | 0.08439 | 12.81887 | 0.000972 | 0.002992 | 0.06974 |
| 165 | 21P | RM13B | 5 | 11.42979 | 0.00117 | 0.002312 | 0.137 | 6.17963 | 0.000262 | 0.001498 | 0.08001 |
| 166 | 21P | RM16B | 3 | 12.852 | 4.78E-04 | 0.001255 | 0.09124 | 6.83555 | 0.012582 | 0.018639 | 0.05924 |
| 167 | 21P | RM178B | 5 | 12.24447 | 7.38E-04 | 0.00161 | 0.12338 | 6.72323 | 8.13E-05 | 0.001084 | 0.09641 |
| 168 | 21P | RM306B | 1 | 7.81347 | 0.00653 | 0.00795 | 0.09105 | 7.71103 | 0.001287 | 0.00355 | 0.07655 |
| 169 | 21P | RM338B | 3 | 10.71402 | 0.00126 | 0.002378 | 0.05338 | - | - | - | - |
| 170 | 21P | RM5428A | 8 | 6.14969 | 0.01517 | 0.016549 | 0.06898 | - | - | - | - |
| 171 | 21P | RM570C | 3 | 14.68378 | 1.91E-04 | 0.00063 | 0.09493 | 8.45989 | 0.000317 | 0.001689 | 0.06209 |
| 172 | 28P | RM1350B | 3 | 5.44792 | 0.02195 | 0.023192 | 0.06023 | 3.87269 | 0.007685 | 0.012809 | 0.05278 |
| 173 | 28P | RM16B | 3 | 11.81489 | 7.93E-04 | 0.001687 | 0.0845 | 7.64195 | 6.52E-05 | 0.001043 | 0.0584 |
| 174 | 28P | RM306A | 1 | 7.12681 | 0.00924 | 0.011009 | 0.08372 | 8.37517 | 0.000564 | 0.00205 | 0.0832 |
| 175 | 28P | RM334A | 5 | 6.67337 | 0.01158 | 0.01297 | 0.07612 | 5.43062 | 0.005632 | 0.010012 | 0.06984 |
| 176 | 28P | RM3452D | 8 | 10.44036 | 0.00146 | 0.002609 | 0.05369 | - | - | - | - |
| 177 | 28P | RM5428A | 8 | 6.90339 | 0.01025 | 0.011795 | 0.07679 | - | - | - | - |
| 178 | 28P | RM570C | 3 | 10.06399 | 0.00186 | 0.003064 | 0.06706 | - | - | - | - |
| 179 | 7P | RM1216A | 1 | 17.35638 | 4.90E-05 | 0.000235 | 0.09166 | 16.99782 | 0.004532 | 0.009541 | 0.09133 |
| 180 | 7P | RM3857A | 2 | - | - | - | - | 4.86767 | 0.091309 | 0.104353 | 0.06739 |
| 181 | 7P | RM13D | 5 | 8.32E-04 | 0.001704 | 0.14461 | - | 5.44137 | 0.002348 | 0.005524 | 0.0587 |
| 182 | 7P | RM334A | 5 | - | - | - | - | 6.30286 | 0.112739 | 0.127029 | 0.06965 |
| 183 | 7P | RM16200D | 3 | 1.44E-04 | 0.000515 | 0.09331 | - | 9.70856 | 0.00325 | 0.007026 | 0.06957 |
| 184 | 7P | RM306B | 1 | - | - | - | - | 9.87191 | 0.03831 | 0.048648 | 0.09518 |
| 185 | 7P | RM178A | 5 | 0.00486 | 0.006329 | 0.08762 | - | 7.60512 | 0.001329 | 0.003429 | 0.07534 |
| 186 | 7P | RM254A | 11 | 0.01419 | 0.015581 | 0.05251 | - | - | - | - | - |
| 187 | 7P | RM5784A | 5 | 0.01916 | 0.020634 | 0.05115 | - | - | - | - | - |
| 188 | AC | RM1216A | 1 | 0.00144 | 0.002601 | 0.0575 | - | - | - | - | - |
| 189 | AC | RM3825D | 1 | 6.84E-04 | 0.001553 | 0.08396 | - | - | - | - | - |
| 190 | AP | RM164A | 5 | 0.00204 | 0.003327 | 0.05079 | - | - | - | - | - |
| 191 | AP | RM205D | 9 | 5.73E-04 | 0.001375 | 0.06194 | - | - | - | - | - |
| 192 | AP | RM20AA | 12 | 9.26E-05 | 0.000389 | 0.08719 | - | - | - | - | - |
| 193 | AP | RM257A | 9 | 5.35E-04 | 0.001322 | 0.06752 | - | - | - | - | - |
| 194 | AP | RM274B | 5 | 2.73E-05 | 0.000143 | 0.12012 | - | - | - | - | - |
| 195 | AP | RM334B | 5 | 0.00253 | 0.003864 | 0.10708 | - | 8.16872 | 0.005166 | 0.009612 | 0.09177 |
| 196 | AP | RM178A | 5 | - | - | - | - | 5.99646 | 0.011119 | 0.017791 | 0.05439 |
| 197 | AP | RM341D | 2 | 0.00177 | 0.002944 | 0.05449 | - | - | - | - | - |
| 198 | AP | RM3428A | 11 | 3.73E-04 | 0.001028 | 0.06702 | - | - | - | - | - |
| 199 | AP | RM3685A | 2 | 2.19E-04 | 0.000693 | 0.07449 | - | - | - | - | - |
| 200 | AP | RM3744D | 9 | 0.00154 | 0.002667 | 0.05235 | - | - | - | - | - |
| 201 | AP | RM518C | 4 | 0.00549 | 0.007041 | 0.05196 | - | - | - | - | - |
| 202 | AP | RM5529A | 2 | 0.00531 | 0.006862 | 0.05957 | - | - | - | - | - |
| 203 | AU | RM1216A | 1 | 1.20E-04 | 0.000457 | 0.08267 | - | 12.52982 | 0.001314 | 0.003504 | 0.06811 |
| 204 | AU | RM1350B | 3 | 0.02352 | 0.024391 | 0.0589 | - | 3.69916 | 0.006797 | 0.011569 | 0.05184 |
| 205 | AU | RM13B | 5 | 0.00286 | 0.004178 | 0.11698 | - | 5.26377 | 0.000476 | 0.001903 | 0.06435 |
| 206 | AU | RM16B | 3 | 1.36E-04 | 0.000498 | 0.10786 | - | 7.75675 | 0.000102 | 0.001167 | 0.06519 |
| 207 | AU | RM178A | 5 | 0.00232 | 0.003643 | 0.10171 | - | 4.85633 | 0.000472 | 0.001988 | 0.07045 |
| 208 | AU | RM22B | 3 | 0.00117 | 0.002286 | 0.0589 | - | - | - | - | - |
| 209 | AU | RM306B | 1 | 0.00279 | 0.004112 | 0.10898 | - | 9.976 | 0.000467 | 0.002199 | 0.09792 |
| 210 | AU | RM334A | 5 | 0.03507 | 0.03528 | 0.05368 | - | - | - | - | - |
| 211 | AU | RM3452D | 8 | 1.99E-04 | 0.000642 | 0.07266 | - | - | - | - | - |
| 212 | AU | RM5428A | 8 | 0.00888 | 0.010733 | 0.07965 | - | - | - | - | - |
| 213 | AU | RM570C | 3 | 1.34E-04 | 0.0005 | 0.09926 | - | 8.20831 | 0.000471 | 0.002093 | 0.0612 |
| 214 | BC | RM306A | 1 | 0.01153 | 0.013088 | 0.07905 | - | - | - | - | - |
| 215 | BC | RM334B | 5 | 0.00358 | 0.004812 | 0.10006 | - | 7.00284 | 0.025898 | 0.035116 | 0.09129 |
| 216 | BC | RM3823C | 9 | 0.00302 | 0.004336 | 0.07718 | - | 7.80354 | 0.000904 | 0.003015 | 0.07444 |
| 217 | BC | RM400E | 6 | 8.23E-04 | 0.001706 | 0.11166 | - | 6.48408 | 0.051057 | 0.061888 | 0.10148 |
| 218 | BC | RM5392A | 10 | 0.00164 | 0.002811 | 0.08968 | - | 13.46365 | 0.002575 | 0.005885 | 0.11962 |
| 219 | BC | RM570A | 3 | 0.00599 | 0.007399 | 0.05271 | - | - | - | - | - |
| 220 | BC | RM81D | 3 | 0.00129 | 0.002408 | 0.05374 | - | 9.86374 | 0.17744 | 0.184353 | 0.05076 |
| 221 | DH | K39512A | 11 | 1.48E-04 | 0.000518 | 0.08736 | - | - |  | - | - |
| 222 | DH | RM11229C | 1 | 2.30E-04 | 0.000702 | 0.09805 | - | - | - | - | - |
| 223 | DH | RM1232A | 1 | 7.17E-08 | 1.09E-06 | 0.1524 | - | - | - | - | - |
| 224 | DH | RM1335A | 7 | 0.00988 | 0.011527 | 0.05507 | - | - | - | - | - |
| 225 | DH | RM133A | 6 | 4.21E-04 | 0.001122 | 0.07832 | - | - | - | - | - |
| 226 | DH | RM1385A | 2 | 2.94E-06 | 2.74E-05 | 0.11172 | - | - | - | - | - |
| 227 | DH | RM13B | 5 | 0.034 | 0.03441 | 0.06093 | - | - | - | - | - |
| 228 | DH | RM335A | 4 | - | - | - | - | 16.46749 | 0.223258 | 0.226084 | 0.09108 |
| 229 | DH | RM147A | 10 | 1.18E-05 | 7.93E-05 | 0.14181 | - | - | - | - | - |
| 230 | DH | RM14C | 1 | 0.00437 | 0.005781 | 0.08494 | - | - | - | - | - |
| 231 | DH | RM159A | 5 | 4.38E-08 | 7.35E-07 | 0.14774 | - | - | - | - | - |
| 232 | DH | RM16200B | 3 | 2.80E-05 | 0.000143 | 0.11215 | - | - | - | - | - |
| 233 | DH | RM164A | 5 | 4.16E-07 | 5.37E-06 | 0.13097 | - | - | - | - | - |
| 234 | DH | RM169A | 5 | 0.00133 | 0.002455 | 0.05487 | - | - | - | - | - |
| 235 | DH | RM173A | 5 | 0.00117 | 0.002259 | 0.06393 | - | - | - | - | - |
| 236 | DH | RM205B | 9 | 1.16E-07 | 1.62E-06 | 0.1405 | - | - | - | - | - |
| 237 | DH | RM209D | 11 | 1.21E-05 | 7.24E-05 | 0.10228 | - | - | - | - | - |
| 238 | DH | RM20AA | 12 | 1.23E-06 | 1.38E-05 | 0.13107 | - | - | - | - | - |
| 239 | DH | RM210A | 8 | 1.14E-04 | 0.000447 | 0.086 | - | - | - | - | - |
| 240 | DH | RM242A | 9 | 6.32E-05 | 0.000279 | 0.08099 | - | - | - | - | - |
| 241 | DH | RM253A | 6 | 3.11E-04 | 0.000901 | 0.06665 | - | - | - | - | - |
| 242 | DH | RM254A | 11 | 0.00169 | 0.002839 | 0.08464 | - | - | - | - | - |
| 243 | DH | RM25790A | 10 | 0.00591 | 0.007355 | 0.05007 | - | - | - | - | - |
| 244 | DH | RM257A | 9 | 8.16E-12 | 3.43E-10 | 0.23849 | - | - | - | - | - |
| 245 | DH | RM2615C | 6 | 1.62E-09 | 3.4E-08 | 0.18347 | - | - | - | - | - |
| 246 | DH | RM274A | 5 | 1.66E-10 | 4.64E-09 | 0.25691 | - | - | - | - | - |
| 247 | DH | RM306B | 1 | 1.53E-05 | 8.59E-05 | 0.2143 | - | - | - | - | - |
| 248 | DH | RM310B | 8 | 0.00292 | 0.004229 | 0.05305 | - | - | - | - | - |
| 249 | DH | RM3286A | 5 | 1.19E-05 | 7.43E-05 | 0.18719 | - | 6.18599 | 3.05E-06 | 0.000122 | 0.06505 |
| 250 | DH | RM334A | 5 | 3.10E-04 | 0.000913 | 0.14922 | - | 6.74451 | 0.009296 | 0.015177 | 0.07226 |
| 251 | DH | RM341D | 2 | 2.43E-09 | 4.54E-08 | 0.1845 | - | - | - | - | - |
| 252 | DH | RM3428A | 11 | 4.01E-11 | 1.35E-09 | 0.21253 | - | - | - | - | - |
| 253 | DH | RM3452A | 8 | 3.94E-04 | 0.001069 | 0.06615 | - | - | - | - | - |
| 254 | DH | RM3482A | 1 | 3.37E-05 | 0.000167 | 0.10533 | - | - | - | - | - |
| 255 | DH | RM3685A | 2 | 1.94E-14 | 1.63E-12 | 0.28246 | - | - | - | - | - |
| 256 | DH | RM39A | 5 | 4.86E-04 | 0.001237 | 0.07817 | - | - | - | - | - |
| 257 | DH | RM409A | 9 | 4.59E-17 | 7.71E-15 | 0.319 | - | - | - | - | - |
| 258 | DH | RM424A | 2 | 0.00335 | 0.004576 | 0.07241 | - | - | - | - | - |
| 259 | DH | RM426B | 3 | 0.009 | 0.0108 | 0.05056 | - | - | - | - | - |
| 260 | DH | RM434B | 9 | 3.45E-06 | 2.9E-05 | 0.11023 | - | - | - | - | - |
| 261 | DH | RM5364A | 12 | 1.06E-04 | 0.000423 | 0.08834 | - | - | - | - | - |
| 262 | DH | RM536B | 11 | 1.14E-05 | 7.97E-05 | 0.10123 | - | - | - | - | - |
| 263 | DH | RM5428A | 8 | 7.17E-04 | 0.001607 | 0.12951 | - | - | - | - | - |
| 264 | DH | RM5474A | 3 | 4.53E-06 | 3.46E-05 | 0.11112 | - | - | - | - | - |
| 265 | DH | RM5478B | 4 | 5.71E-07 | 6.85E-06 | 0.12871 | - | - | - | - | - |
| 266 | DH | RM551C | 4 | 5.92E-10 | 1.42E-08 | 0.19727 | - | - | - | - | - |
| 267 | DH | RM5626C | 3 | 5.10E-05 | 0.000238 | 0.10138 | - | - | - | - | - |
| 268 | DH | RM5784A | 5 | 0.0011 | 0.0022 | 0.09695 | - | - | - | - | - |
| 269 | DH | RM5784C | 5 | 0.00249 | 0.003838 | 0.08385 | - | - | - | - | - |
| 270 | DH | RM6251A | 9 | 2.02E-13 | 1.13E-11 | 0.25017 | - | - | - | - | - |
| 271 | DH | RM6917B | 6 | 3.02E-06 | 2.67E-05 | 0.22279 | - | 5.46674 | 0.002882 | 0.006405 | 0.09345 |
| 272 | DH | RM7025A | 12 | 0.00353 | 0.004783 | 0.05133 | - | - | - | - | - |
| 273 | DH | RM85B | 3 | 1.62E-05 | 8.77E-05 | 0.09788 | - | - | - | - | - |
| 274 | DH | sbq1A | 11 | 0.00139 | 0.002538 | 0.05304 | - | - | - | - | - |
| 275 | FL | RM11229B | 1 | 0.00624 | 0.007652 | 0.05529 | - | - | - | - | - |
| 276 | FL | RM1216A | 1 | 7.35E-04 | 0.001624 | 0.06431 | - | 10.82643 | 0.011667 | 0.017949 | 0.05963 |
| 277 | FL | RM146A | 5 | 0.00148 | 0.00259 | 0.05389 | - | - | - | - | - |
| 278 | FL | RM400C | 6 | 0.00246 | 0.003827 | 0.09247 | - | 4.21288 | 0.000138 | 0.001225 | 0.07252 |
| 279 | FW | RM13B | 5 | - | - | - | - | 10.05949 | 0.165856 | 0.174585 | 0.06168 |
| 280 | FW | RM5428A | 8 | - | - | - | - | 9.98477 | 0.395565 | 0.395565 | 0.05959 |
| 281 | FW | RM1235A | 8 | 3.20E-04 | 0.000912 | 0.07704 | - | 14.16484 | 0.141623 | 0.155203 | 0.07727 |
| 282 | FW | RM426G | 3 | 2.89E-04 | 0.000868 | 0.09508 | - | 12.42178 | 0.165259 | 0.176276 | 0.0686 |
| 283 | FW | RM6292C | 1 | 1.33E-06 | 1.39E-05 | 0.13029 | - | 26.57417 | 0.000942 | 0.003013 | 0.14301 |
| 284 | FW | RM81A | 3 | 5.81E-06 | 4.24E-05 | 0.1038 | - | 13.20993 | 0.153693 | 0.166154 | 0.06932 |
| 285 | GY | RM400C | 6 | 0.01168 | 0.012995 | 0.06508 | - | 5.31033 | 0.005326 | 0.009684 | 0.05954 |
| 286 | GY | RM202D | 11 | - | - | - | - | 9.59195 | 6.03E-07 | 4.83E-05 | 0.05075 |
| 287 | GY | RM1350A | 3 | 0.02972 | 0.030445 | 0.05439 | - | - | - | - | - |
| 288 | GY | RM551C | 4 | 0.00226 | 0.003582 | 0.05203 | - | - | - | - | - |
| 289 | IL | RM101B | 12 | 0.00576 | 0.007276 | 0.05245 | - | 11.1122 | 0.06778 | 0.078586 | 0.07672 |
| 290 | IL | RM5428A | 8 | - | - | - | - | 6.71818 | 0.032218 | 0.042253 | 0.08644 |
| 291 | IL | RM336D | 7 | - | - | - | - | 7.73402 | 0.060545 | 0.072293 | 0.05228 |
| 292 | IL | RM13A | 5 | 0.00451 | 0.005919 | 0.10667 | - | 6.60331 | 0.061918 | 0.072845 | 0.08541 |
| 293 | IL | RM334A | 5 | 0.03694 | 0.03694 | 0.05264 | - | 2.50832 | 0.201321 | 0.206483 | 0.05662 |
| 294 | IL | RM3823A | 9 | 0.01116 | 0.012754 | 0.05711 | - | - | - | - | - |
| 295 | LC | RM1216A | 1 | 4.94E-04 | 0.001239 | 0.06833 | - | 8.73992 | 0.000382 | 0.001909 | 0.05489 |
| 296 | LC | RM3823C | 9 | 0.0119 | 0.013153 | 0.05613 | - | - | - | - | - |
| 297 | LC | RM3825D | 1 | 2.49E-06 | 2.46E-05 | 0.15516 | - | - | - | - | - |
| 298 | LS | RM1216A | 1 | 4.83E-04 | 0.001249 | 0.06855 | - | 11.44726 | 0.012132 | 0.018313 | 0.0625 |
| 299 | LS | RM306A | 1 | 0.02016 | 0.021436 | 0.06728 | - | - | - | - | - |
| 300 | LS | RM434B | 9 | 5.94E-04 | 0.001405 | 0.06193 | - | - | - | - | - |
| 301 | MP | RM1216A | 1 | 6.20E-04 | 0.001447 | 0.06603 | - | 10.10785 | 0.005086 | 0.009688 | 0.05547 |
| 302 | MP | RM310B | 8 | - | - | - | - | 8.74714 | 0.004792 | 0.009583 | 0.05137 |
| 303 | MP | RM1350B | 3 | 0.01014 | 0.011748 | 0.07525 | - | 4.62975 | 0.004886 | 0.009534 | 0.06502 |
| 304 | MP | RM13B | 5 | 0.00311 | 0.004391 | 0.1151 | - | 5.13334 | 0.000689 | 0.002397 | 0.06299 |
| 305 | MP | RM16B | 3 | 1.80E-04 | 0.000604 | 0.1042 | - | 7.79114 | 0.000127 | 0.001266 | 0.06247 |
| 306 | MP | RM178A | 5 | 0.00557 | 0.007089 | 0.08501 | - | 4.50322 | 0.001172 | 0.003472 | 0.05903 |
| 307 | MP | RM22B | 3 | 8.07E-04 | 0.001695 | 0.06265 | - | - | - | - | - |
| 308 | MP | RM306B | 1 | 0.00313 | 0.004382 | 0.10651 | - | 10.71379 | 0.000561 | 0.002139 | 0.10715 |
| 309 | MP | RM334A | 5 | 0.01793 | 0.019434 | 0.06723 | - | 5.85431 | 0.011184 | 0.017543 | 0.06289 |
| 310 | MP | RM3452D | 8 | 3.45E-04 | 0.000967 | 0.06741 | - | - | - | - | - |
| 311 | MP | RM5428A | 8 | 0.01154 | 0.013012 | 0.07442 | - | - | - | - | - |
| 312 | MP | RM570C | 3 | 5.25E-05 | 0.000238 | 0.11064 | - | 9.66654 | 6.33E-05 | 0.001265 | 0.07521 |
| 313 | NT | RM215A | 9 | 0.00164 | 0.002783 | 0.05317 | - | - | - | - | - |
| 314 | NT | RM3117C | 3 | 8.54E-04 | 0.001728 | 0.0731 | - | 10.81095 | 0.005664 | 0.00985 | 0.08098 |
| 315 | NT | RM400E | 6 | 0.00334 | 0.004599 | 0.0871 | - | 5.62073 | 0.045751 | 0.056309 | 0.09034 |
| 316 | NT | RM5481C | 7 | 0.00267 | 0.004041 | 0.05213 | - | - | - | - | - |
| 317 | NT | sbq1A | 11 | 1.31E-05 | 7.6E-05 | 0.09633 | - | - | - | - | - |
| 318 | PH | RM1350B | 3 | 0.02339 | 0.024407 | 0.059 | - | 3.97649 | 0.001263 | 0.003609 | 0.05648 |
| 319 | PH | RM3286A | 5 | - | - | - | - | 3.36554 | 0.022491 | 0.031022 | 0.05088 |
| 320 | PH | RM336B | 7 | 0.00222 | 0.003552 | 0.06534 | - | - | - | - | - |
| 321 | PH | RM400B | 6 | 0.00937 | 0.011086 | 0.06895 | - | 3.39171 | 0.015943 | 0.022776 | 0.05592 |
| 322 | PL | RM5529A | 2 | - | - | - | - | 7.52482 | 0.11402 | 0.126689 | 0.06146 |
| 323 | PL | RM400B | 6 | - | - | - | - | 3.55231 | 0.039984 | 0.04998 | 0.0548 |
| 324 | PL | RM1216A | 1 | 0.0022 | 0.003554 | 0.05319 | - | 10.71505 | 0.036253 | 0.046778 | 0.05876 |
| 325 | PL | RM1350B | 3 | 0.02665 | 0.027467 | 0.05649 | - | 4.48531 | 0.004695 | 0.009632 | 0.05393 |
| 326 | PL | RM13C | 5 | 7.62E-04 | 0.00164 | 0.14656 | - | 7.95011 | 0.000235 | 0.001565 | 0.14198 |
| 327 | PL | RM16200B | 3 | 0.00579 | 0.007259 | 0.05032 | - | - | - | - | - |
| 328 | PL | RM178A | 5 | 0.03375 | 0.034364 | 0.05077 | - | - | - | - | - |
| 329 | PL | RM3286A | 5 | 0.00306 | 0.004357 | 0.09046 | - | 10.14105 | 0.001378 | 0.003444 | 0.08847 |
| 330 | PL | RM3452B | 8 | 6.54E-04 | 0.001504 | 0.06134 | - | 9.34752 | 0.029422 | 0.039229 | 0.05555 |
| 331 | PL | RM3823B | 9 | 0.00974 | 0.011443 | 0.05919 | - | 5.45017 | 0.017008 | 0.023871 | 0.0613 |
| 332 | PL | RM569A | 3 | 0.00117 | 0.002234 | 0.06635 | - | - | - | - | - |
| 333 | CT | RM5784C | 5 | - | - | - | - | 7.03834 | 0.013976 | 0.020329 | 0.05308 |
| **Pooled** | | | | | | | | | | | |
| 334 | 14P | RM5428A | 8 | 13.51093 | 0.000422 | 0.002075 | 0.14045 | 6.49067 | 0.014814 | 0.028488 | 0.07565 |
| 335 | 14P | RM13B | 5 | 11.43512 | 0.00118 | 0.003165 | 0.12646 | 3.84933 | 2.54E-06 | 3.81E-05 | 0.06331 |
| 336 | 14P | RM16B | 3 | 10.32694 | 0.00166 | 0.003696 | 0.07317 | 3.91203 | 0.225228 | 0.248413 | 0.05253 |
| 337 | 14P | RM257B | 9 | - | - | - | - | 9.09814 | 0.158642 | 0.201663 | 0.05155 |
| 338 | 14P | RM22B | 3 | 13.11362 | 0.000385 | 0.001975 | 0.06856 | - | - | - | - |
| 339 | 14P | RM400D | 6 | 6.53327 | 0.01219 | 0.014984 | 0.06463 | - | - | - | - |
| 340 | 14P | RM335A | 4 | - | - | - | - | 9.04542 | 0.003678 | 0.011494 | 0.05601 |
| 341 | 14P | RM5392A | 10 | 7.0208 | 0.0093 | 0.012059 | 0.06218 | - | - | - | - |
| 342 | 14P | RM242A | 9 | 12.55126 | 0.000499 | 0.002264 | 0.0602 | - | - | - | - |
| 343 | 14P | RM334A | 5 | 5.07772 | 0.02697 | 0.027916 | 0.0587 | 4.80307 | 0.365767 | 0.370709 | 0.05209 |
| 344 | 14P | RM310A | 8 | 10.27131 | 0.00163 | 0.003699 | 0.05765 | - | - | - | - |
| 345 | 14P | RM3452D | 8 | 9.97096 | 0.00186 | 0.003991 | 0.05022 | - | - | - | - |
| 346 | 21P | RM13B | 5 | 9.11013 | 0.00353 | 0.005785 | 0.09543 | 2.43216 | 2.74E-05 | 0.000256 | 0.05317 |
| 347 | 21P | RM178A | 5 | 8.05746 | 0.00565 | 0.007937 | 0.08308 | 4.26829 | 0.012172 | 0.024673 | 0.05277 |
| 348 | 21P | RM85D | 3 | 16.53506 | 7.13E-05 | 0.000647 | 0.08245 | 10.30165 | 0.00015 | 0.001021 | 0.05605 |
| 349 | 21P | RM334A | 5 | 7.13575 | 0.00915 | 0.011997 | 0.08178 | 7.57822 | 0.085251 | 0.122958 | 0.07147 |
| 350 | 21P | RM6917C | 6 | 6.75007 | 0.01103 | 0.014147 | 0.06315 | - | - | - | - |
| 351 | 21P | RM570C | 3 | 9.47401 | 0.00251 | 0.004628 | 0.0625 | - | - | - | - |
| 352 | 21P | RM310B | 8 | 10.75951 | 0.00127 | 0.003258 | 0.06116 | - | - | - | - |
| 353 | 21P | RM5428A | 8 | 5.17224 | 0.02556 | 0.026691 | 0.0593 | - | - | - | - |
| 354 | 21P | RM335A | 4 | 9.3351 | 0.00267 | 0.004566 | 0.05881 | 9.21204 | 0.000279 | 0.001608 | 0.05682 |
| 355 | 21P | RM257B | 9 | 10.09002 | 0.00177 | 0.003868 | 0.05465 | 12.14412 | 0.064544 | 0.10085 | 0.06736 |
| 356 | 28P | RM306B | 1 | 8.15687 | 0.00551 | 0.007929 | 0.09395 | 8.37581 | 0.000564 | 0.002487 | 0.08321 |
| 357 | 28P | RM16B | 3 | 12.26485 | 0.000638 | 0.00215 | 0.08734 | 7.64778 | 0.0017 | 0.00607 | 0.05843 |
| 358 | 28P | RM334A | 5 | 6.70109 | 0.01144 | 0.01421 | 0.07723 | 5.43109 | 0.005632 | 0.015085 | 0.06985 |
| 359 | 28P | RM570C | 3 | 9.91981 | 0.002 | 0.00414 | 0.06657 | - | - | - | - |
| 360 | 28P | RM1350B | 3 | 5.69486 | 0.01926 | 0.021645 | 0.06143 | 3.8737 | 0.005643 | 0.014595 | 0.05279 |
| 361 | 28P | RM310B | 8 | 10.4572 | 0.00148 | 0.003564 | 0.06 | - | - | - | - |
| 362 | 28P | RM335A | 4 | 7.89655 | 0.00563 | 0.008004 | 0.05042 | - | - | - | - |
| 363 | 7P | RM518A | 4 | 9.20357 | 0.00287 | 0.004838 | 0.05641 | 9.96582 | 0.00028 | 0.001499 | 0.05641 |
| 364 | 7P | RM5784B | 5 | 6.69443 | 0.01105 | 0.01402 | 0.05598 | 6.30404 | 7.91E-05 | 0.000659 | 0.05598 |
| 365 | AC | RM3825D | 1 | 11.54179 | 0.000902 | 0.002728 | 0.07986 | - | - | - | - |
| 366 | AP | RM334B | 5 | 9.79892 | 0.00244 | 0.004644 | 0.09134 | 8.16872 | 0.005166 | 0.014903 | 0.09177 |
| 367 | AP | RM178A | 5 | 5.57152 | 0.02052 | 0.02242 | 0.05466 | 5.99561 | 0.004637 | 0.013911 | 0.05438 |
| 368 | AU | RM13B | 5 | 12.94596 | 0.000589 | 0.002173 | 0.13699 | 4.08509 | 2.31E-07 | 1.73E-05 | 0.07107 |
| 369 | AU | RM16B | 3 | 19.23749 | 2.4E-05 | 0.000257 | 0.1297 | 9.58062 | 0.248697 | 0.266461 | 0.0648 |
| 370 | AU | RM334A | 5 | 9.7556 | 0.00249 | 0.004664 | 0.10694 | 10.31544 | 0.048695 | 0.083002 | 0.09325 |
| 371 | AU | RM5428A | 8 | 8.79929 | 0.00395 | 0.006299 | 0.0969 | 4.34986 | 0.009677 | 0.021346 | 0.05212 |
| 372 | AU | RM400C | 6 | 8.38593 | 0.0047 | 0.00702 | 0.0806 | - | - | - | - |
| 373 | AU | RM310A | 8 | 13.70284 | 0.000293 | 0.001647 | 0.07645 | - | - | - | - |
| 374 | AU | RM570C | 3 | 11.48111 | 0.000915 | 0.002633 | 0.07356 | - | - | - | - |
| 375 | AU | RM178A | 5 | 6.72523 | 0.01117 | 0.014022 | 0.07216 | - | - | - | - |
| 376 | AU | RM22B | 3 | 12.43283 | 0.00054 | 0.002198 | 0.06626 | - | - | - | - |
| 377 | AU | RM3117A | 3 | 8.51159 | 0.00409 | 0.00635 | 0.05301 | 8.06281 | 0.008528 | 0.019989 | 0.06315 |
| 378 | AU | RM3825D | 1 | 7.56131 | 0.00681 | 0.009237 | 0.05206 | - | - | - | - |
| 379 | BC | RM5392A | 10 | 14.59 | 0.000227 | 0.001407 | 0.11998 | 13.46365 | 0.002575 | 0.008395 | 0.11962 |
| 380 | BC | RM400E | 6 | 11.20093 | 0.00118 | 0.003094 | 0.10125 | 6.48408 | 0.082615 | 0.121493 | 0.10148 |
| 381 | BC | RM334B | 5 | 8.5487 | 0.0045 | 0.006808 | 0.09123 | 7.00284 | 0.025898 | 0.046246 | 0.09129 |
| 382 | BC | RM3823C | 9 | 8.96922 | 0.0034 | 0.005651 | 0.07453 | 7.80354 | 0.000904 | 0.003768 | 0.07444 |
| 383 | BC | RM81D | 3 | 10.30038 | 0.00157 | 0.003705 | 0.05091 | 9.86339 | 0.186789 | 0.215526 | 0.05076 |
| 384 | DH | RM335A | 4 | 52.8077 | 2E-11 | 2.36E-09 | 0.16493 | 18.13411 | 0.179703 | 0.210589 | 0.10143 |
| 385 | DH | RM6917B | 6 | 19.27342 | 3.21E-05 | 0.000315 | 0.14842 | 6.33647 | 0.000461 | 0.00216 | 0.11232 |
| 386 | DH | RM3286A | 5 | 20.08034 | 2.13E-05 | 0.00028 | 0.12834 | 6.80161 | 1.34E-06 | 2.52E-05 | 0.07118 |
| 387 | DH | RM13D | 5 | 11.9837 | 0.000913 | 0.002692 | 0.11167 | 2.70951 | 0.285269 | 0.301341 | 0.065 |
| 388 | DH | RM5478C | 4 | 29.62632 | 1.69E-07 | 9.95E-06 | 0.08988 | - | - | - | - |
| 389 | DH | RM409A | 9 | 26.77467 | 5.97E-07 | 2.35E-05 | 0.08117 | - | - | - | - |
| 390 | DH | RM334A | 5 | 9.66751 | 0.0026 | 0.004512 | 0.07362 | 7.72855 | 0.008594 | 0.019531 | 0.08577 |
| 391 | DH | RM5709C | 4 | 15.11513 | 0.000159 | 0.001172 | 0.06713 | - | - | - | - |
| 392 | DH | RM202D | 11 | 18.97044 | 2.19E-05 | 0.000258 | 0.05829 | - | - | - | - |
| 393 | DH | RM14A | 1 | 7.96047 | 0.00587 | 0.008149 | 0.05692 | - | - | - | - |
| 394 | FL | RM400C | 6 | 9.64479 | 0.00251 | 0.004557 | 0.09255 | 4.21288 | 0.000138 | 0.001034 | 0.07252 |
| 395 | FL | RM1216A | 1 | 11.49016 | 0.000869 | 0.002698 | 0.06286 | 10.8252 | 0.134644 | 0.187006 | 0.05962 |
| 396 | FL | RM146A | 5 | 10.56937 | 0.00137 | 0.003368 | 0.05488 | - | - | - | - |
| 397 | FW | RM6292C | 1 | 25.89065 | 9.64E-07 | 2.84E-05 | 0.13418 | 26.57417 | 0.243191 | 0.264338 | 0.14301 |
| 398 | FW | RM13B | 5 | - | - | - | - | 10.05949 | 0.165856 | 0.200632 | 0.06168 |
| 399 | FW | RM81A | 3 | 22.08655 | 5.04E-06 | 8.5E-05 | 0.10559 | 13.20993 | 0.153693 | 0.19874 | 0.06932 |
| 400 | FW | RM426G | 3 | 13.60602 | 0.000329 | 0.001766 | 0.09395 | 12.42178 | 0.165259 | 0.203188 | 0.0686 |
| 401 | FW | RM1235A | 8 | 14.36962 | 0.000212 | 0.001471 | 0.08186 | 14.16484 | 0.141623 | 0.193122 | 0.07727 |
| 402 | FW | RM5428A | 8 | 5.63568 | 0.01994 | 0.022197 | 0.06431 | 10.00709 | 0.153056 | 0.20139 | 0.05971 |
| 403 | GY | RM8027C | 10 | 21.03828 | 8.64E-06 | 0.000127 | 0.10823 | 15.74215 | 0.16674 | 0.1985 | 0.10273 |
| 404 | GY | RM185B | 4 | 9.53039 | 0.00251 | 0.004488 | 0.07135 | 8.08077 | 0.430298 | 0.430298 | 0.06217 |
| 405 | GY | RM13B | 5 | 5.3934 | 0.02308 | 0.024535 | 0.07059 | 2.90746 | 0.006275 | 0.015689 | 0.05604 |
| 406 | GY | RM551B | 4 | 12.61719 | 0.000492 | 0.002322 | 0.06716 | 10.10558 | 0.078439 | 0.117658 | 0.05539 |
| 407 | GY | RM1216A | 1 | 10.27427 | 0.00161 | 0.003725 | 0.05658 | 9.01619 | 0.146435 | 0.196118 | 0.05369 |
| 408 | GY | RM400A | 6 | 5.23506 | 0.02438 | 0.025686 | 0.05044 | - | - | - | - |
| 409 | IL | RM13A | 5 | 8.51803 | 0.00471 | 0.006947 | 0.10704 | 6.60331 | 0.061918 | 0.098806 | 0.08541 |
| 410 | IL | RM336D | 7 | - | - | - | - | 7.7362 | 0.060545 | 0.098715 | 0.05229 |
| 411 | IL | RM5428A | 8 | 9.02122 | 0.00354 | 0.005722 | 0.09625 | 6.71818 | 0.032218 | 0.056194 | 0.08644 |
| 412 | IL | RM101A | 12 | 12.46463 | 0.000561 | 0.002134 | 0.07907 | 11.11896 | 0.06778 | 0.103745 | 0.07676 |
| 413 | IL | RM3823A | 9 | 6.42347 | 0.01268 | 0.015114 | 0.05409 | - | - | - | - |
| 414 | IL | RM334A | 5 | 4.2405 | 0.04272 | 0.04272 | 0.05021 | 2.50957 | 0.201321 | 0.228773 | 0.05665 |
| 415 | LC | RM3825D | 1 | 23.42337 | 3.6E-06 | 7.07E-05 | 0.14923 | - | - | - | - |
| 416 | LC | RM1216A | 1 | 11.02044 | 0.0011 | 0.00309 | 0.05828 | 8.73845 | 0.000382 | 0.001909 | 0.05488 |
| 417 | LC | RM3823C | 9 | 6.34044 | 0.01325 | 0.015635 | 0.0541 | - | - | - | - |
| 418 | LS | RM1216A | 1 | 11.61437 | 0.000816 | 0.002603 | 0.06275 | 11.44726 | 0.012132 | 0.025998 | 0.0625 |
| 419 | MP | RM5428A | 8 | 14.63476 | 0.000254 | 0.001496 | 0.14726 | 7.81197 | 5.21E-06 | 5.58E-05 | 0.07769 |
| 420 | MP | RM570C | 3 | 23.5257 | 3.26E-06 | 7.69E-05 | 0.14426 | 8.54695 | 4.14E-06 | 5.18E-05 | 0.06228 |
| 421 | MP | RM13B | 5 | 8.71093 | 0.00428 | 0.006559 | 0.1021 | - | - | - | - |
| 422 | MP | RM178A | 5 | 8.77056 | 0.00396 | 0.00623 | 0.09254 | - | - | - | - |
| 423 | MP | RM16B | 3 | 12.32326 | 0.00062 | 0.002216 | 0.08804 | - | - | - | - |
| 424 | MP | RM338B | 3 | 16.03117 | 8.95E-05 | 0.000754 | 0.0781 | - | - | - | - |
| 425 | MP | RM400C | 6 | 7.12897 | 0.00894 | 0.011853 | 0.07047 | - | - | - | - |
| 426 | MP | RM335A | 4 | 10.74623 | 0.0013 | 0.003264 | 0.06809 | 8.00136 | 0.000953 | 0.003761 | 0.05214 |
| 427 | MP | RM22B | 3 | 12.01484 | 0.000666 | 0.002182 | 0.06493 | - | - | - | - |
| 428 | MP | RM1335B | 7 | 7.63067 | 0.00667 | 0.009152 | 0.06058 | - | - | - | - |
| 429 | MP | RM5784B | 5 | 6.47829 | 0.01239 | 0.014919 | 0.05864 | 6.54418 | 0.023288 | 0.0426 | 0.05782 |
| 430 | MP | RM310B | 8 | 9.64802 | 0.00224 | 0.004405 | 0.05619 | - | - | - | - |
| 431 | MP | RM6292C | 1 | 9.5914 | 0.00229 | 0.00443 | 0.05421 | - | - | - | - |
| 432 | MP | RM334A | 5 | 4.27123 | 0.042 | 0.042359 | 0.05065 | - | - | - | - |
| 433 | MP | RM5392A | 10 | 5.54247 | 0.02042 | 0.022519 | 0.05006 | - | - | - | - |
| 434 | NT | RM426G | 3 | 12.69109 | 0.000513 | 0.002162 | 0.08818 | 9.8981 | 0.007764 | 0.018783 | 0.07148 |
| 435 | NT | RM81D | 3 | 15.86418 | 9.74E-05 | 0.000766 | 0.0781 | 15.23654 | 0.0002 | 0.001248 | 0.08094 |
| 436 | NT | RM334A | 5 | 6.20369 | 0.01482 | 0.017314 | 0.07181 | 4.59423 | 0.001091 | 0.004091 | 0.05523 |
| 437 | NT | RM6917A | 6 | 6.00782 | 0.01627 | 0.018822 | 0.06514 | 3.85893 | 0.0592 | 0.098667 | 0.05247 |
| 438 | NT | RM335A | 4 | 9.70818 | 0.00221 | 0.00442 | 0.06169 | - | - | - | - |
| 439 | NT | RM22B | 3 | 10.99919 | 0.00111 | 0.003046 | 0.05972 | - | - | - | - |
| 440 | NT | RM254A | 11 | 6.48572 | 0.01224 | 0.01489 | 0.05519 | - | - | - | - |
| 441 | NT | RM1350E | 3 | 4.95248 | 0.02873 | 0.029479 | 0.05465 | - | - | - | - |
| 442 | NT | RM518C | 4 | 8.00948 | 0.00532 | 0.00775 | 0.05263 | - | - | - | - |
| 443 | NT | RM5428A | 8 | 4.40215 | 0.03897 | 0.039642 | 0.05083 | - | - | - | - |
| 444 | PH | RM400C | 6 | 9.62816 | 0.00253 | 0.004456 | 0.08146 | - | - | - | - |
| 445 | PH | RM13C | 5 | - | - | - | - | 3.92046 | 0.015461 | 0.028989 | 0.0718 |
| 446 | PH | RM335A | 4 | 14.36029 | 0.00022 | 0.00144 | 0.07904 | 8.4574 | 5.59E-07 | 2.09E-05 | 0.05261 |
| 447 | PH | RM5529A | 2 | - | - | - | - | 6.54284 | 0.005392 | 0.014979 | 0.05658 |
| 448 | PH | RM5364A | 12 | 12.56858 | 0.000513 | 0.002242 | 0.06655 | 9.62552 | 0.20335 | 0.227631 | 0.05856 |
| 449 | PH | RM101A | 12 | 9.86256 | 0.00206 | 0.004191 | 0.05723 | 8.11508 | 0.116456 | 0.164796 | 0.05627 |
| 450 | PH | RM85D | 3 | 12.41268 | 0.000541 | 0.002126 | 0.0569 | 9.33208 | 6.98E-07 | 1.75E-05 | 0.0517 |
| 451 | PH | RM3452A | 8 | 12.12194 | 0.000623 | 0.002163 | 0.05631 | 10.89826 | 0.285324 | 0.297213 | 0.05844 |
| 452 | PH | RM1350B | 3 | 6.00602 | 0.01633 | 0.018708 | 0.05225 | - | - | - | - |
| 453 | PL | RM13C | 5 | 5.52625 | 0.02151 | 0.023074 | 0.07192 | 4.38112 | 0.002397 | 0.00817 | 0.07192 |
| 454 | PL | RM16200C | 3 | 9.93761 | 0.00196 | 0.00413 | 0.06326 | 10.90549 | 0.309906 | 0.318396 | 0.06328 |
| 455 | PL | RM5529A | 2 | 7.51767 | 0.007 | 0.009386 | 0.05605 | 6.87973 | 0.160135 | 0.200169 | 0.05605 |
| 456 | CT | RM5784C | 5 | 5.73604 | 0.01841 | 0.020888 | 0.05214 | 7.03662 | 0.013976 | 0.027585 | 0.05306 |

GLM-general linear model, MLM-mixed linear model, PDI-percent disease index, 7P- PDI of 7^th^ day, 14P-PDI 14^th^ day, 21P-PDI of 21^st^ day, 28P-PDI of 28^th^ day, MP-mean PDI, AUDPC-area under disease progress curve, AC- auricle colour, AP-apiculus colour, BC-basal leaf sheath colour, LC-ligule colour, LS-ligule shape, PH-plant height, PL-panicle length, NT-tiller number per plant, IL-internodal length, FL- flag leaf length, FW-flag leaf width, CT-culm thickness, GY- grain yield.

**Supplementary Table 10.** Marker loci highly significant association with sheath blight resistance over season.

| **QTL** | **Marker** | **Chromosome no** | **Position** | | **Year 1** | | | | | | | | **Year 2** | | | | | | | | | **Pooled** | | | | | | | |
| --- | --- | --- | --- | --- | --- | --- | --- | --- | --- | --- | --- | --- | --- | --- | --- | --- | --- | --- | --- | --- | --- | --- | --- | --- | --- | --- | --- | --- | --- |
|  |  |  | **SSR Start** | **SSR End** | **7**  **PDI** | **14**  **PDI** | **21**  **PDI** | **28**  **PDI** | **MP** | **AUDPC** | **BC** | **PH** | **7**  **PDI** | **14**  **PDI** | **21**  **PDI** | **28**  **PDI** | **MP** | **AUDPC** | **BC** | **PH** | **7**  **PDI** | | **14**  **PDI** | **21**  **PDI** | **28**  **PDI** | **MP** | **AUDPC** | **BC** | **PH** |
| **qShB.1-1** | **RM1216** | **1** | **32101663** | **32101690** | **-** | **-** | **-** | **-** | **-** | **-** | **-** | **-** | **+** | **-** | **+** | **-** | **+** | **+** | **-** | **-** | **-** | | **-** | **-** | **-** | **-** | **-** | **-** | **-** |
| **qShB.1-2** | **RM306** | **1** | **24447001** | **24447461** | **-** | **-** | **-** | **-** | **-** | **-** | **-** | **-** | **+** | **+** | **+** | **+** | **+** | **+** | **-** | **-** | **-** | | **-** | **-** | **+** | **-** | **-** | **-** | **-** |
| **qShB.1-3** | **RM3482** | **1** | **39713330** | **39713379** | **-** | **+** | **+** | **-** | **-** | **+** | **-** | **-** | **-** | **-** | **-** | **-** | **-** | **-** | **-** | **-** | **-** | | **-** | **-** | **-** | **-** | **-** | **-** | **-** |
| **qShB.2-1** | **RM250** | **2** | **32798753** | **32798806** | **-** | **+** | **+** | **-** | **-** | **+** | **-** | **-** | **-** | **-** | **-** | **-** | **-** | **-** | **-** | **-** | **-** | | **-** | **-** | **-** | **-** | **-** | **-** | **-** |
| **qShB.2-2** | **RM3857** | **2** | **31209378** | **31209427** | **-** | **-** | **-** | **-** | **-** | **-** | **-** | **-** | **+** | **-** | **-** | **-** | **-** | **-** | **-** | **-** | **-** | | **-** | **-** | **-** | **-** | **-** | **-** | **-** |
| **qph.2-1** | **RM5529** | **2** | **2219381** | **2219547** | **-** | **-** | **-** | **-** | **-** | **-** | **-** | **-** | **-** | **-** | **-** | **-** | **-** | **-** | **-** | **-** | **-** | | **-** | **-** | **-** | **-** | **-** | **-** | **+** |
| **qShB.3-1** | **RM1350** | **3** | **28479832** | **28479877** | **-** | **-** | **-** | **-** | **-** | **-** | **-** | **-** | **-** | **-** | **-** | **+** | **+** | **+** | **-** | **+** | **-** | | **-** | **-** | **+** | **-** | **-** | **-** | **-** |
| **qShB.3-2** | **RM16** | **3** | **23126064** | **23126256** | **-** | **-** | **-** | **-** | **-** | **-** | **-** | **-** | **-** | **+** | **+** | **+** | **+** | **+** | **-** | **-** | **-** | | **+** | **-** | **+** | **-** | **+** | **-** | **-** |
| **qShB.3-3** | **RM16200** | **3** | **35674153** | **35674176** | **-** | **-** | **-** | **-** | **-** | **-** | **-** | **-** | **+** | **-** | **-** | **-** | **-** | **-** | **-** | **-** | **-** | | **-** | **-** | **-** | **-** | **-** | **-** | **-** |
| **qShB.3-4** | **RM3117** | **3** | **3768739** | **3768781** | **-** | **-** | **-** | **-** | **-** | **-** | **-** | **-** | **-** | **-** | **-** | **-** | **-** | **-** | **-** | **-** | **-** | | **-** | **-** | **-** | **-** | **+** | **-** | **-** |
| **qShB.3-5** | **RM570** | **3** | **35381850** | **35381879** | **-** | **-** | **-** | **-** | **-** | **-** | **-** | **-** | **-** | **-** | **+** | **-** | **+** | **+** | **-** | **-** | **-** | | **-** | **-** | **-** | **+** | **-** | **-** | **-** |
| **qShB.3-6** | **RM85** | **3** | **36348147** | **36348202** | **-** | **-** | **-** | **-** | **-** | **-** | **-** | **-** | **-** | **-** | **-** | **-** | **-** | **-** | **-** | **-** | **-** | | **-** | **+** | **-** | **-** | **-** | **-** | **+** |
| **qBc.3-1** | **RM81** | **3** | **1927336** | **1927362** | **-** | **-** | **-** | **-** | **-** | **-** | **-** | **-** | **-** | **-** | **-** | **-** | **-** | **-** | **+** | **-** | **-** | | **-** | **-** | **-** | **-** | **+** | **+** | **-** |
| **qShB.4-1** | **RM335** | **4** | **688353** | **688453** | **-** | **-** | **-** | **-** | **-** | **-** | **-** | **-** | **-** | **-** | **-** | **-** | **-** | **-** | **-** | **-** | **-** | | **+** | **+** | **-** | **+** | **-** | **-** | **+** |
| **qShB.4-2** | **RM518** | **4** | **2030135** | **2030291** | **-** | **-** | **-** | **-** | **-** | **-** | **-** | **-** | **-** | **-** | **-** | **-** | **-** | **-** | **-** | **-** | **+** | | **-** | **-** | **-** | **-** | **-** | **-** | **-** |
| **qShB.5-1** | **RM13** | **5** | **8194923** | **8195062** | **-** | **-** | **-** | **-** | **-** | **-** | **-** | **-** | **+** | **+** | **+** | **-** | **+** | **+** | **-** | **-** | **-** | | **+** | **+** | **-** | **-** | **+** | **-** | **-** |
| **qShB.5-2** | **RM178** | **5** | **25101829** | **25101945** | **-** | **-** | **-** | **-** | **-** | **-** | **-** | **-** | **+** | **-** | **+** | **-** | **+** | **+** | **-** | **-** | **-** | | **-** | **+** | **-** | **-** | **-** | **-** | **-** |
| **qShB.5-3** | **RM334** | **5** | **28547326** | **28547583** | **-** | **-** | **-** | **-** | **-** | **-** | **-** | **-** | **+** | **-** | **-** | **+** | **+** | **-** | **+** | **-** | **-** | | **+** | **+** | **+** | **-** | **+** | **+** | **-** |
| **qShB.5-4** | **RM5784** | **5** | **27637500** | **27637556** | **-** | **-** | **-** | **-** | **-** | **-** | **-** | **-** | **-** | **-** | **-** | **-** | **-** | **-** | **-** | **-** | **+** | | **-** | **-** | **-** | **+** | **-** | **-** | **-** |
| **qPh.5-1** | **RM3286** | **5** | **29323329** | **29323354** | **-** | **-** | **-** | **-** | **-** | **-** | **-** | **-** | **-** | **-** | **-** | **-** | **-** | **-** | **-** | **+** | **-** | | **-** | **-** | **-** | **-** | **-** | **-** | **-** |
| **qsShB.6.1** | **RM400** | **6** | **28049016** | **28049204** | **-** | **-** | **+** | **-** | **-** | **-** | **-** | **-** | **-** | **-** | **-** | **-** | **-** | **-** | **-** | **+** | **-** | | **-** | **-** | **-** | **-** | **-** | **+** | **-** |
| **qPh.6-1** | **RM6917** | **6** | **4079415** | **4079489** | **-** | **-** | **-** | **-** | **-** | **-** | **-** | **+** | **-** | **-** | **-** | **-** | **-** | **-** | **-** | **-** | **-** | | **-** | **-** | **-** | **-** | **-** | **-** | **-** |
| **qShB.8-1** | **RM310** | **8** | **5110450** | **5110493** | **-** | **-** | **-** | **-** | **-** | **-** | **-** | **-** | **-** | **-** | **-** | **-** | **+** | **-** | **-** | **-** | **-** | | **-** | **-** | **-** | **-** | **-** | **-** | **-** |
| **qShB.8-2** | **RM5428** | **8** | **3252017** | **3252247** | **-** | **-** | **-** | **-** | **+** | **-** | **-** | **-** | **-** | **-** | **-** | **-** | **-** | **-** | **-** | **-** | **-** | | **+** | **-** | **-** | **+** | **+** | **-** | **-** |
| **qPh.8-1** | **RM3452** | **8** | **24776783** | **24776995** | **-** | **-** | **-** | **-** | **-** | **-** | **-** | **-** | **-** | **-** | **-** | **-** | **-** | **-** | **-** | **-** | **-** | | **-** | **-** | **-** | **-** | **-** | **-** | **+** |
| **qShB.9.1** | **RM257** | **9** | **17666088** | **17666147** | **-** | **+** | **+** | **-** | **-** | **-** | **-** | **-** | **-** | **-** | **-** | **-** | **-** | **-** | **-** | **-** | **-** | | **+** | **+** | **-** | **-** | **-** | **-** | **-** |
| **qBc.9-1** | **RM3823** | **9** | **21841165** | **21841206** | **-** | **-** | **-** | **-** | **-** | **-** | **-** | **-** | **-** | **-** | **-** | **-** | **-** | **-** | **+** | **-** | **-** | | **-** | **-** | **-** | **-** | **+** | **-** | **-** |
| **qShB.10-1** | **RM5392** | **10** | **17303525** | **17303875** | **-** | **-** | **-** | **-** | **+** | **-** | **-** | **-** | **-** | **-** | **-** | **-** | **-** | **-** | **+** | **-** | **-** | | **-** | **-** | **-** | **-** | **-** | **+** | **-** |
| **qShB.10-2** | **RM8015** | **10** | **7162790** | **7162833** | **-** | **-** | **-** | **-** | **+** | **-** | **-** | **-** | **-** | **-** | **-** | **-** | **-** | **-** | **-** | **-** | **-** | | **-** | **-** | **-** | **-** | **-** | **-** | **-** |
| **qPh.12-1** | **RM101** | **12** | **8826555** | **8826855** | **-** | **-** | **-** | **-** | **-** | **-** | **-** | **-** | **-** | **-** | **-** | **-** | **-** | **-** | **-** | **-** | **-** | | **-** | **-** | **-** | **-** | **-** | **-** | **+** |

14PDI- PDI of 14^th^ day, 21PDI-PDI of 21^st^ day, 28PDI- PDI of 28day, 7PDI-PDI of 7^th^ day, AUDPC, BC- Basal leaf sheath colour, MP- mean PDI, PH- Plant height.

**Supplementary Table 11.** Favorable and non-favorable allelic variation of 30 QTLs regulating sheath blight resistance in the panel population.

| **QTL** | **Marker** | **Chromosome no** | **Alleles** | **7PDI** | **14PDI** | **21PDI** | **28PDI** | **Mean PDI** | **AUDPC** | **BC** | **PH** |
| --- | --- | --- | --- | --- | --- | --- | --- | --- | --- | --- | --- |
|  |  |  |  | **FA* and N-FA (Mean ± SD)** | **FA* and N-FA**  **(Mean ± SD)** | **FA* and N-FA**  **(Mean ± SD)** | **FA* and N-FA**  **(Mean ± SD)** | **FA* and N-FA**  **(Mean ± SD)** | **FA* and N-FA**  **(Mean ± SD)** | **FA* and N-FA (Mean ± SD)** | **FA* and N-FA (Mean ± SD)** |
| **qShB.1-1** | **RM1216** | **1** | **A** | **12.29±2.15 (S2)*** | **-** | **30.65±8.11 (S2)*** | **-** | **26.56±5.18 (S2)*** | **770.76±168.66 (S2)*** | **-** | **-** |
|  |  |  | **B** | **11.66±1.47 (S2)** | **-** | **28.65±8.82 (S2)** | **-** | **24.34±6.24 (S2)** | **709.90±195.36 (S2)** | **-** | **-** |
|  |  |  | **C** | **11.88±1.98 (S2)** | **-** | **31.36±8.79 (S2)** | **-** | **26.33±6.26 (S2)** | **770.64±200.93 (S2)** | **-** | **-** |
|  |  |  | **D** | **12.04±2.56 (S2)** | **-** | **31.29±8.74 (S2)** | **-** | **26.08±7.06 (S2)** | **761.78±207.77 (S2)** | **-** | **-** |
| **qShB.1-2** | **RM306** | **1** | **A** | **12.18±2.27 (S2)** | **18.65±6.23 (S2)** | **29.41±10.11 (S2)** | **41.29±9.52 (P) 41.29±9.52 (S2)** | **24.74±7.15 (S2)** | **725.48±213.34 (S2)** | **-** | **-** |
|  |  |  | **B** | **11.80±1.91 (S2)*** | **21.21±8.27 (S2)*** | **30.48±8.51 (S2)*** | **38.74±12.58 (P)* 38.74±12.58 (S)*** | **26.19±6.34 (S2)*** | **769.81±203.00 (S2)*** | **-** | **-** |
| **qShB.1-3** | **RM3482** | **1** | **A** | **-** | **22.35±8.52 (S1)** | **33.63±9.21 (S1)** | **-** | **-** | **440.27±128.54 (S1)** | **-** | **-** |
|  |  |  | **B** | **-** | **28.07±7.74 (S1)*** | **39.77±7.73 (S1)*** | **-** |  | **532.16±114.11 (S1)*** | **-** | **-** |
|  |  |  | **C** | **-** | **22.26±5.97 (S1)** | **32.56±6.86 (S1)** | **-** | **-** | **434.34±89.34 (S1)** | **-** | **-** |
|  |  |  | **D** | **-** | **20.56±6.08 (S1)** | **31.77±4.59 (S1)** | **-** | **-** | **408.86±79.25 (S1)** | **-** | **-** |
|  |  |  | **E** | **-** | **21.37±5.38 (S1)** | **31.01±6.38 (S1)** | **-** | **-** | **418.25±68.97 (S1)** | **-** | **-** |
| **qShB.2-1** | **RM250** | **2** | **A** | **-** | **23.83±7.30 (S1)** | **34.53±7.86 (S1)** | **-** | **-** | **457.46±110.50 (S1)** | **-** | **-** |
|  |  |  | **B** | **-** | **22.70±6.95 (S1)** | **33.09±7.0(S1)** | **-** | **-** | **441.40±99.95 (S1)** | **-** | **-** |
|  |  |  | **C** | **-** | **19.02±5.95 (S1)*** | **29.41±9.11 (S1)*** | **-** | **-** | **379.43±88.86 (S1)*** | **-** | **-** |
| **qShB.2-2** | **RM3857** | **2** | **A** | **11.95±1.76 (S2)*** | **-** | **-** | **-** | **-** | **-** | **-** | **-** |
|  |  |  | **B** | **11.89±1.94 (S2)** | **-** | **-** | **-** | **-** | **-** | **-** | **-** |
| **qPh.2-1** | **RM5529** | **2** | **A** | **-** | **-** | **-** | **-** | **-** | **-** | **-** | **112.35±11.57 (P)*** |
|  |  |  | **B** | **-** | **-** | **-** | **-** | **-** | **-** | **-** | **100.90±17.44 (P)** |
| **qShB.3-1** | **RM1350** | **3** | **A** | **-** | **-** | **-** | **41.65±9.29 (P) 41.65±9.29 (S2)** | **26.07±6.46 (S2)** | **762.25±208.09 (S2)** | **-** | **-** |
|  |  |  | **B** | **-** | **-** | **-** | **35.46±7.89 (P)* 35.69±7.89 (S2)*** | **22.35±4.91 (S2)*** | **647.28±151.02 (S2)*** | **-** | **-** |
|  |  |  | **C** | **-** | **-** | **-** | **49.17±10.98 (P) 49.17±11.30 (S2)** | **30.19±7.18 (S2)** | **876.62±220.16 (S2)** | **-** | **-** |
|  |  |  | **D** | **-** | **-** | **-** | **41.30±20.90 (P) 50.42±20.90 (S2)** | **27.12±7.47 (S2)** | **747.15±197.62 (S2)** | **-** | **-** |
| **qShB.3-2** | **RM16** | **3** | **A** | **-** | **24.42±7.36 (P) 24.31±11.66 (S2)** | **32.83±10.46 (S2)** | **43.85±12.10 (P) 43.85±12.10 (S2)** | **28.10±8.34 (S2)** | **849.26±188.52 (P) 833.26±266.69 (S2)** | **-** | **-** |
|  |  |  | **B** | **-** | **20.38±5.32 (P)* 17.96±6.08 (S2)*** | **28.11±7.84 (S2)*** | **38.04±10.72 (P)* 38.04±10.72 (S2)*** | **24.02±5.81 (S2)*** | **754.94±148.53 (P)* 700.64±173.79 (S2)*** | **-** | **-** |
|  |  |  | **C** | **-** | **25.46±3.56 (P) 25.50±7.00 (S2)** | **38.91±11.60 (S2)** | **52.65±8.12 (P) 52.65±8.12 (S2)** | **32.25±6.43 (S2)** | **914.47±146.34 (P) 944.32±214.65 (S2)** | **-** | **-** |
| **qShB.3-3** | **RM16200** | **3** | **A** | **11.72±1.68 (S2)** | **-** | **-** | **-** | **-** | **-** | **-** | **-** |
|  |  |  | **B** | **11.73±1.65 (S2)** | **-** | **-** | **-** | **-** | **-** | **-** | **-** |
|  |  |  | **C** | **11.11±0.00 (S2)** | **-** | **-** | **-** | **-** | **-** | **-** | **-** |
|  |  |  | **D** | **15.09±3.83 (S2)*** | **-** | **-** | **-** | **-** | **-** | **-** | **-** |
| **qShB.3-4** | **RM3117** | **3** | **A** | **-** | **-** | **-** | **-** | **-** | **760.26±120.14 (P)*** | **-** | **-** |
|  |  |  | **B** | **-** | **-** | **-** | **-** | **-** | **779.89±141.53 (P)** | **-** | **-** |
|  |  |  | **C** | **-** | **-** | **-** | **-** | **-** | **837.44±156.48 (P)** | **-** | **-** |
| **qShB.3-5** | **RM570** | **3** | **A** | **-** | **-** | **28.28±7.97 (S2)** | **-** | **25.53±4.66 (P) 24.10±5.31 (S2)** | **699.07±163.66 (S2)** | **-** | **-** |
|  |  |  | **B** | **-** | **-** | **29.07±7.89 (S2)** | **-** | **26.41±4.56 (P) 25.00±5.54 (S2)** | **726.09±177.21 (S2)** | **-** | **-** |
|  |  |  | **C** | **-** | **-** | **37.80±8.89 (S2)*** | **-** | **30.47±5.29 (P)* 31.21±7.01 (S2)*** | **926.53±220.11 (S2)*** | **-** | **-** |
|  |  |  | **D** | **-** | **-** | **33.75±11.96 (S2)** | **-** | **28.54±6.57 (P) 27.95±8.86 (S2)** | **832.71±293.52 (S2)** | **-** | **-** |
| **qShB.3-6** | **RM85** | **3** | **A** | **-** | **-** | **31.08±5.35 (P)** | **-** | **-** | **-** | **-** | **-** |
|  |  |  | **B** | **-** | **-** | **30.30±5.29 (P)** | **-** | **-** | **-** | **-** | **-** |
|  |  |  | **C** | **-** | **-** | **30.52±6.52 (P)** | **-** | **-** | **-** | **-** | **-** |
|  |  |  | **D** | **-** | **-** | **35.73±6.86 (P)*** | **-** | **-** | **-** | **-** | **-** |
| **qBc.3-1** | **RM81** | **3** | **A** | **-** | **-** | **-** | **-** | **-** | **-** | **1.00±0.00 (P) (S2)** | **-** |
|  |  |  | **B** | **-** | **-** | **-** | **-** | **-** | **-** | **1.18±0.53 (P) (S2)** | **-** |
|  |  |  | **C** | **-** | **-** | **-** | **-** | **-** | **-** | **1.75±1.50 (P) (S2)*** | **-** |
| **qShB.4-1** | **RM335** | **4** | **A** | **-** | **20.20±5.20 (P)* 21.96±6.80 (S1)*** | **30.49±5.94 (P)* 32.93±6.96 (S1)*** | **-** | **25.38±4.77 (P)*** | **-** | **-** | **-** |
|  |  |  | **B** | **-** | **23.79±6.28 (P) 27.20±7.46 (S1)** | **35.45±6.67 (P) 38.04±8.71 (S1)** | **-** | **29.39±6.09 (P)** | **-** | **-** | **-** |
| **qShB.4-2** | **RM518** | **4** | **A** | **11.54±0.92 (P)*** | **-** | **-** | **-** | **-** | **-** | **-** | **-** |
|  |  |  | **B** | **12.52±2.37 (P)** | **-** | **-** | **-** | **-** | **-** | **-** | **-** |
|  |  |  | **C** | **12.16±1.40 (P)** | **-** | **-** | **-** | **-** | **-** | **-** | **-** |
| **qShB.5-1** | **RM13** | **5** | **A** | **11.11±0.00 (S2)** | **18.52±3.76 (P) 18.15±8.94 (S2)** | **31.57±2.64 (P) 29.81±4.47 (S2)** | **-** | **23.56±3.53 (S2)** | **730.05±66.03 (P) 704.45±129.22 (S2)** | **-** | **-** |
|  |  |  | **B** | **11.67±1.60 (S2)*** | **18.47±4.28 (P)* 17.19±6.20 (S2)*** | **30.04±5.13 (P)* 27.99±7.78 (S2)*** | **-** | **24.23±4.55 (S2)*** | **732.44±103.37 (P)* 696.34±151.71 (S2)*** | **-** | **-** |
|  |  |  | **C** | **11.90±1.93 (S2)** | **23.75±5.80 (P) 24.77±10.20 (S2)** | **34.88±5.19 (P) 36.42±9.30 (S2)** | **-** | **30.04±7.08 (S2)** | **865.40±141.28 (P) 890.56±230.69 (S2)** | **-** | **-** |
|  |  |  | **D** | **14.06±3.97 (S2)** | **24.66±5.01 (P) 23.28±9.02** | **36.94±5.48 (P) 35.94±8.04 (S2)** | **-** | **29.44±7.72 (S2)** | **896.32±152.93 (P) 875.88±229.11 (S2)** | **-** | **-** |
| **qShB.5-2** | **RM178** | **5** | **A** | **11.53±1.43 (S2)*** | **-** | **30.68±5.18 (P)* 28.33±7.33 (S2)*** | **-** | **24.42±4.97 (S2)*** | **707.48±156.79 (S2)*** | **-** | **-** |
|  |  |  | **B** | **12.43±2.67 (S2)** | **-** | **33.71±6.28 (P) 33.37±99 (S2)** | **-** | **27.08±6.35 (S2)** | **801.00±203.27 (S2)** | **-** | **-** |
| **qShB.5-3** | **RM334** | **5** | **A** | **11.59±1.08 (S2)*** | **20.92±2.82 (P)*** | **29.48±3.89 (P)*** | **32.21±7.67 (P)* 32.21±7.67 (S2)*** | **21.62±5.17 (S2)*** | **724.41±89.34 (P)*** | **-** | **-** |
|  |  |  | **B** | **12.12±1.96 (S2)** | **21.61±4.17 (P)** | **32.29±4.99 (P)** | **42.97±10.32 (P) 42.97±11.16 (S2)** | **27.52±7.05 (S2)** | **800.61±121.74 (P)** | **-** | **-** |
|  |  |  | **C** | **12.08±2.33 (S2)** | **21.77±5.99 (P)** | **32.60±6.52 (P)** | **41.81±10.51 (P) 41.81±10.51 (S2)** | **26.17±7.12 (S2)** | **802.62±161.25 (P)** | **-** | **-** |
|  |  |  | **D** | **11.83±2.12 (S2)** | **21.69±6.82 (P)** | **31.70±8.02 (P)** | **42.26±12.52 (P) 42.26±12.52 (S2)** | **26.77±8.47 (S2)** | **795.49±191.44 (P)** | **-** | **-** |
|  |  |  | **E** | **11.57±1.13 (S2)** | **17.70±3.57 (P)** | **27.02±4.43 (P)** | **33.92±9.03 (P) 33.92±9.03 (S2)** | **21.40±5.11 (S2)** | **669.62±99.46 (P)** | **-** | **-** |
| **qShB.5-4** | **RM5784** | **5** | **A** | **12.55±2.64 (P)** | **-** | **-** | **-** | **26.91±6.19 (P)** | **-** | **-** | **-** |
|  |  |  | **B** | **11.30±0.79 (P)*** | **-** | **-** | **-** | **25.22±3.13 (P)*** | **-** | **-** | **-** |
|  |  |  | **C** | **12.00±1.27 (P)** | **-** | **-** | **-** | **26.84±5.40 (P)** | **-** | **-** | **-** |
| **qPh.5-1** | **RM3286** | **5** |  | **-** | **-** | **-** | **-** | **-** | **-** | **-** | **110.17±16.94*** |
|  |  |  |  | **-** | **-** | **-** | **-** | **-** | **-** | **-** | **117.28±23.48** |
| **qsShB.6.1** | **RM400** | **6** | **A** | **-** | **-** | **35.45±9.49 (S1)** | **-** | **-** | **-** | **-** | **-** |
|  |  |  | **B** | **-** | **-** | **32.32±7.81 (S1)*** | **-** | **-** | **-** | **-** | **-** |
|  |  |  | **C** | **-** | **-** | **33.47±6.94 (S1)** | **-** | **-** | **-** | **-** | **-** |
|  |  |  | **D** | **-** | **-** | **35.65±6.16 (S1)** | **-** | **-** | **-** | **-** | **-** |
|  |  |  | **E** | **-** | **-** | **35.56±3.14 (S1)** | **-** | **-** | **-** | **-** | **-** |
| **qPh.6-1** | **RM6917** | **6** | **A** | **-** | **-** | **-** | **-** | **-** | **-** | **-** | **108.50±15.11 (S1)** |
|  |  |  | **B** | **-** | **-** | **-** | **-** | **-** | **-** | **-** | **114.34±16.34 (S1)** |
|  |  |  | **C** | **-** | **-** | **-** | **-** | **-** | **-** | **-** | **110.91±11.59 (S1)*** |
| **qShB.8-1** | **RM310** | **8** | **A** | **-** | **-** | **-** | **-** | **26.87±7.62 (S2)** | **-** | **-** | **-** |
|  |  |  | **B** | **-** | **-** | **-** | **-** | **24.14±5.85 (S2)*** | **-** | **-** | **-** |
|  |  |  | **C** | **-** | **-** | **-** | **-** | **25.38±6.22 (S2)** | **-** | **-** | **-** |
| **qShB.8-2** | **RM5428** | **8** | **A** | **-** | **20.49±5.47 (P)*** | **-** | **-** | **25.64±5.05 (P)* 23.15±5.16 (S1)*** | **766.28±148.92 (P)*** | **-** | **-** |
|  |  |  | **B** | **-** | **24.17±6.51 (P)** | **-** | **-** | **29.65±5.19 (P) 22.77±3.15 (S1)** | **870.20±149.79 (P)** | **-** | **-** |
| **qPh.8-1** | **RM3452** | **8** | **A** | **-** | **-** | **-** | **-** | **-** | **-** | **-** | **111.18±16.34 (P)*** |
|  |  |  | **B** | **-** | **-** | **-** | **-** | **-** | **-** | **-** | **107.12±13.45 (P)** |
|  |  |  | **C** | **-** | **-** | **-** | **-** | **-** | **-** | **-** | **110.22±11.24 (P)** |
|  |  |  | **D** | **-** | **-** | **-** | **-** | **-** | **-** | **-** | **111.73±11.54 (P)** |
| **qShB.9.1** | **RM257** | **9** | **A** | **-** | **20.07±5.03 (P)** | **30.93±5.01 (P)** | **-** | **-** | **-** | **-** | **-** |
|  |  |  | **B** | **-** | **19.49±4.79 (P)** | **29.73±7.38 (P)** | **-** | **-** | **-** | **-** | **-** |
|  |  |  | **C** | **-** | **23.45±6.57 (P)*** | **33.84±8.38 (P)*** | **-** | **-** | **-** | **-** | **-** |
|  |  |  | **D** | **-** | **20.63±3.81 (P)** | **34.03±6.18 (P)** | **-** | **-** | **-** | **-** | **-** |
| **qBc.9-1** | **RM3823** | **9** | **A** | **-** | **-** | **-** | **-** | **-** | **-** | **1.05±0.22 (P) 1.05±0.22 (S2)** | **-** |
|  |  |  | **B** | **-** | **-** | **-** | **-** | **-** | **-** | **1.06±0.24 (P) 1.06±0.24 (S2)** | **-** |
|  |  |  | **C** | **-** | **-** | **-** | **-** | **-** | **-** | **1.38±0.72 (P)* 1.38±0.72 (S2)*** | **-** |
| **qShB.10-1** | **RM5392** | **10** | **A** | **-** | **-** | **-** | **-** | **24.28±4.63 (S1)*** | **-** | **-** | **-** |
|  |  |  | **B** | **-** | **-** | **-** | **-** | **22.10±5.565 (S1)** | **-** | **-** | **-** |
| **qShB.10-2** | **RM8015** | **10** | **A** | **-** | **-** | **-** | **-** | **24.47±4.12 (S1)*** | **-** | **-** | **-** |
|  |  |  | **B** | **-** | **-** | **-** | **-** | **22.91±4.87 (S1)** | **-** | **-** | **-** |
| **qPh.12-1** | **RM101** | **12** | **A** | **-** | **-** | **-** | **-** | **-** | **-** | **-** | **109.58±12.99 (P)*** |
|  |  |  | **B** | **-** | **-** | **-** | **-** | **-** | **-** | **-** | **118.42±23.18 (P)** |

14P- PDI of 14^th^ day, 21P-PDI of 21^st^ day, 28P- PDI of 28day, 7P-PDI of 7^th^ day, AU- AUDPC, BC- Basal leaf sheath color, MP- mean PDI, PH- Plant height. FA*-favourble allele, N-FA – non favourable allele, S1-dry season 2019, S2- wet season 2019, P-pooled data (S1+S2), SD-standard deviation.

**Supplementary Table 12.** Favorable and non-favorable allelic variation of 30 QTLs regulating sheath blight resistance in the panel population.

| **Sl. No.** | **Marker Associated** | **QTL** | **Rap DB** | **MSU ID** | **Description** | **Chromosome** | **QTL location** | **Trait associated** |
| --- | --- | --- | --- | --- | --- | --- | --- | --- |
| 8 | RM16200 | qShB.3-3 | Os03g0848700 | LOC_Os03g63150 | BPH14: Coiled-coil, nucleotide-binding, and leucine-rich repeat (CC-NB-LRR) protein, Resistance to brown planthopper, (Nipponbare: BPH-susceptible) | chr03 | 0.02 Mb left of QTL | 7PDI |
| 13 | RM85 | qShB.3-6 | Os03g0860100 | LOC_Os03g64260 | OsERF83: Ethylene response factor, Transcriptional activator, Positive regulation of disease resistance | chr03 | 0.05 Mb right of QTL | 21PDI |
| 18 | RM3286 | qPh.5-1 | Os05g0578900 | LOC_Os05g50270 | NL1, nl1, OsGATA15, SNFL1: GAGA-type zinc finger transcription factor, Organogenesis, Regulation of leaf development | chr05 | 0.50 Mb left of QTL | PH |
| 19 | RM334 | qShB.5-3 | Os05g0572000 | LOC_Os05g49700 | Pathogenesis-related transcriptional factor and ERF domain containing protein.  OsRPH1: ethylene response factor 52, APETALA2/ethylene-responsive element binding protein 53, Reduced Plant Height 1 | chr05 | 0.05 Mb right of QTL | 7PDI, 14PDI, 21PDI, 28PDI, MP and AUDPC |
| 24 | RM3452 | qPh.8-1 | Os08g0504700 | LOC_Os08g39450 | SAP11, OsDOG, OsSAP11: Gibberellin-induced A20/AN1 zinc-finger protein, Negative regulation of GA (gibberellin) -mediated cell elongation | chr08 | 0.17 Mb right of QTL | PH |
| 25 | RM5428 | qShB.8-2 | Os08g0155900 | LOC_Os08g05960 | OsDR10: Similar to Pathogen-induced defense-responsive protein. | chr08 | 0.01 Mb right of QTL | 14PDI, MP and AUDPC |
