# Supplementary material for: Dissecting genomic regions and underlying sheath blight resistance traits in rice ( Oryza sativa L.) using a genome‐wide association study
Source: Plant Direct. 2023 Nov 23;7(11):e540. doi: 10.1002/pld3.540 (PMC10667636; doi:10.1002/pld3.540)
Supplement: Supplementary file 1 — Data S1. Peer Review [file PLD3-7-e540-s007.pdf]

Plant Direct
Open Access

Dissecting genomic regions and underlying sheath blight resistance traits in rice (*Oryza sativa* L.) using a genome-wide association study

Naveenkumar Ramasamy[1,2,3,†], Annamalai Anandan[1,4,*†], Prabhukarthikeyan S.R[5], Mahender Anumalla[6], Sangeetha Ganesan[7], Vaish Shyam Saran[2], Singh P.K.[2], Waseem Hussain[6], and Jauhar Ali[6]*

DECISION LETTER – Round 1

September 9, 2023

Dr. Jauhar Ali
International Rice Research Institute
Rice Breeding Innovation Department
Los Banos
Laguna
Los Banos 4031
Philippines

RE: Dissecting genomic regions and underlying sheath blight resistance traits in rice (Oryza sativa L.) using a genome-wide association study

Dear Dr. Ali:

Thank you for submitting to Plant Direct. First, let me apologize for the delay. There were difficulties securing reviewers. All required reviews have been returned and we have now finished our evaluation of your manuscript. The reviewers were supportive of the work but minor revisions are needed before the paper can be accepted for publication in Plant Direct.

Please view the reviewers' comments below and use their suggestions as a guide while you work on your revision.

When uploading the revised version of this article, please be sure to include the following:

-A word document that contains your response to the reviewers. You should respond to each reviewer comment and note the changes made to the manuscript. If you do not agree with a reviewer's comment and choose not to make a suggested revision, please explain why. Please try to provide as complete an answer as possible to each reviewer's criticisms.
-A tracked changes document with each change highlighted
- A clean version of the latest version of the manuscript

To upload your revision, please click the link below.
https://plantdirect.msubmit.net/cgi-bin/main.plex?el=A1Lr2bN3A5oHN1I7A9ftdF1EymlxfQyyjUFgMxadk6gZ

In order to provide as timely a service as possible, we ask that your revision is resubmitted within three months after receipt of this request. If an extension is needed, please send a request, along with a brief explanation, to the editorial office at plantdirect@wiley.com .

Please note that, in addition to publishing reviewer comments, the author's responses to review comments will also be published alongside the final version of the paper. If you would not like the author's responses to be published, please contact the editorial office at plantdirect@wiley.com .

Thank you very much for giving us an opportunity to review your work. I look forward to receiving the next version.

Sincerely,

Brian Kvitko

Hsou-min Li

--- Reviewer comments:
Reviewer #1:

Manuscript tile:
Dissecting genomic regions and underlying sheath blight resistance traits in rice (Oryza sativa L.) using a genome-wide association study
Manuscript No:. 2023-01211-TW
Sheath blight (ShB), caused by Rhizoctonia solani Kühn (R. solani), is one of the most destructive diseases causing severe yield loses in rice. The effective and economical way for managing the disease is developing ShB-resistant rice cultivars which is also considered as environmentally sound strategy. In this study, a core population of 192 rice accessions of cultivated varieties and land races of indica and aus were used in the genome wide association studies. This study also identified promising genomic regions on 10 of 12 chromosomes associated with ShB which would be useful in developing rice varieties with durable disease resistance. However, the following points may be considered to improve the manuscript.
1. It was indicated that from 330 rice diverse genotypes, a core population of 192 accessions were selected representing the whole diversity but it is not mentioned on what basis a core was constituted?
2. In materials and methods, it was not clearly mentioned about the phenotyping of 330 rice accessions? Details about the statistical design used and no. of replicates used and traits recorded may be provided.
3. How it was concluded that all the agro morphological traits mentioned like plant height to grain

yield, were related to Sheath blight? (line no. 545 to 548)
4. On what basis the accessions viz., IC 283139, IC22 277248, Sivappuchithirai Kar, and Bowalia were concluded as novel donors ?

Reviewer #2:

It was very happy reading this manuscript. It is a good piece of work. Manuscript is nicely drafted and covered all the aspects. Methodology adopted and statistical analysis performed is appropriate.

• Title of manuscript: Dissecting genomic regions and underlying sheath blight resistance traits in rice (Oryza sativa L.) using a genome-wide association study
• Manuscript ID: 2023-01211-TW • It was very happy reading this manuscript. It is a good piece of work.

Manuscript is nicely drafted and covered all the aspects. Methodology adopted and statistical analysis performed is appropriate. Results obtained are satisfactory and useful. The manuscript contains useful information for the readers and researchers. My only concern is that the number of genotypes used (192) and number of SSR marker used (133) are very less for Genome Wide Association Study. If these parameters suit the journal criteria then the MS could be accepted for publication. Rice sheath blight is one of the most economically significant rice diseases worldwide. This disease causes significant grain yield and quality losses. Yield losses of up to 50% have been reported under most conducive environments. Sheath blight is a soil borne disease caused by the fungus Rhizoctonia solani AG1-IA. The fungus belongs to the phylum Basidiomycota, family Ceratobasidiaceae. Authors have evaluated a set of 330 traditional landraces and improved rice varieties for ShB resistance and created a core panel of 192 accessions for the GWAS. The core panel of rice accessions were evaluated for morphological and ShB diseaseresistance traits over the two seasons. Furthermore the panel was genotyped with 133 SSR microsatellite markers for association mapping for the ShB disease-resistance. Authors have performed several bio-statistical analyses from the agro-morphological traits/ disease score and SSR based molecular data. They have employed GLM and MLM (Q+K) models of GWAS and identified 30 markertrait associations for 18 targeted traits over seasons. Further, they have identified eight QTLs claimed them to be novel. The novel QTLs were located on 2, 4, 8, 10, and 12 chromosomes, which explained phenotypic variation ranging from 5% to 15%. Further they have identified six candidate genes, and three of them Os05t0566400, Os08t0155900, and Os09t0567300 were associated with defense mechanisms against ShB. In addition, they identified novel donors like IC 283139, IC 277248, Sivappuchithirai Kar, and Bowalia. The manuscript contains some useful information for the readers and researchers working for development of ShB resistant rice varieties. In the era of millions of SNPs, 133 SSR markers for used for genotyping of rice genotypes are very less especially for association mapping. Further, less number of SSRs would generate lower LD which restricted the high resolution mapping. The MTA or QTLs identified through GWAS are putative right now and it needs further validation for its confirmation in another population. However, statistical analysis performed from the agro-morphological data & molecular data by the authors are commendable. Furthermore, interpretation of the results with the help of tables, figures and graphs are also appreciable.

I have few queries to the authors :
• Is a panel of 192 sufficient for GWAS?
• Are a set of 133 SSRs sufficient for GWAS? Will they produce sufficient LD for high resolution mapping?
• Have you noticed any seasonal difference in the agro-morphological traits and disease reaction

while screening the core panel in two different seasons i.e. Kharif and Rabi? Because both the seasons have completely different climatic conditions in terms of temperature, sunshine hours and humidity.

• Is there any plan to validate the outcomes of the current study especially the novel MTA? How it could be done?

• What was basis for claiming the novelty of few MTAs? Have you used any database for it?

Attachment: Reviewer 1 Review Attachment 1 - 09-09-2023 10:16:13

Attachment: Reviewer 2 Review Attachment 1 - 09-09-2023 10:16:13

DECISION LETTER – Round 2

October 4, 2023

Dr. Jauhar Ali
International Rice Research Institute
Rice Breeding Innovation Department
Los Banos
Laguna
Los Banos 4031
Philippines

MSID: 2023-01211-TWR1
MS TITLE: Dissecting genomic regions and underlying sheath blight resistance traits in rice (Oryza sativa L.) using a genome-wide association study

Dear Dr. Jauhar Ali:

I have reviewed your responses to the reviewers and associated minor revisions and am pleased to inform you that your manuscript "Dissecting genomic regions and underlying sheath blight resistance traits in rice (Oryza sativa L.) using a genome-wide association study" has been accepted for publication in Plant Direct.

Your article will appear online in the next available issue of Plant Direct. To ensure your article gets published as quickly as possible, please pay attention to the steps detailed below. We have found that most of the delays happen at this stage, especially at the payment stage, so please respond as quickly as possible when prompted.

License Agreement: Once your article has been accepted it will move to Production and undergo admin and file checking - you may receive an email with any queries we have at this stage. When all required items are received by the publisher and queries resolved, the corresponding author will receive an email from Wiley's Author Services system which will ask them to log on at https://authorservices.wiley.com/bauthor and will present them with the appropriate license for completion. Your article cannot be published until both the signed license agreement and payment of the article fee have been received.

Payment of the Open Access Article Publication Fee: All articles published in Plant Direct are fully open access: immediately and freely available to read, download and share. Plant Direct charges a

publication fee to cover the publication costs. The corresponding author for this manuscript should have already received a quote from the payments team (cs-openaccess@wiley.com) with the estimated article publication fee; please email cs-openaccess@wiley.com if this has not been received. The corresponding author should log on to the Wiley Author Services site, where the publication fee can be paid by credit card or an invoice. Pro Forma can also be requested. Payment of the publication charge must be received before the article will be published online.

Proofs: You will have the opportunity to look over your paper once more when you receive the author proofs for your article. The proofs will be with you in approximately two weeks. Please note that, in addition to publishing reviewer comments, the author's responses to review comments will also be published alongside the final version of the paper. If you would not like the author's responses to be published, please contact the editorial office at plantdirect@wiley.com .

Promotion of your article: You can help your research get the attention it deserves! Check out Wiley's free Promotion Guide for best-practice recommendations for promoting your work at www.wileyauthors.com/eeo/guide. And learn more about Wiley Editing Services which offers professional video, design, and writing services to create shareable video abstracts, infographics, conference posters, lay summaries, and research news stories for your research at www.wileyauthors.com/eeo/promotion.

Thank you again for your contribution to Plant Direct. If you have any questions, feel free to contact the editorial office at plantdirect@wiley.com .

Sincerely,

Brian Kvitko

Hsou-min Li

Editor, Plant Direct

AUTHORS' RESPONSE

Dear Editorial Board Member

Plant Direct

Thank you for allowing us to submit the revised version of this manuscript, "Dissecting genomic regions and underlying sheath blight resistance traits in rice (*Oryza sativa* L.) using a genome-wide association study (Plant Direct: 2023-01211-TW)". We are grateful to the reviewers for the critical review and thoughtful comments on this manuscript and appreciate their time and effort in providing valuable feedback and insightful comments to improve the manuscript. We have revised the manuscript by adding the reviewer-suggested information in track-change mode in a Word file and included point-by-point responses to all comments below in blue font.

Reviewer 1

Sheath blight (ShB), caused by Rhizoctonia solani Kühn (R. solani), is one of the most destructive diseases causing severe yield loses in rice. The effective and economical way for managing the disease

is developing ShB-resistant rice cultivars which is also considered as environmentally sound strategy. In this study, a core population of 192 rice accessions of cultivated varieties and land races of indica and aus were used in the genome wide association studies. This study also identified promising genomic regions on 10 of 12 chromosomes associated with ShB which would be useful in developing rice varieties with durable disease resistance. However, the following points may be considered to improve the manuscript.

Query 1: It was indicated that from 330 rice diverse genotypes, a core population of 192 accessions were selected, representing the whole diversity but it is not mentioned on what basis a core was constituted.

Response: Thanks for the nice comments and critical review. We have included detailed information on phenotypic screening methodology to create a core selection of the 192 accessions in the Materials and Method section (Line numbers 547 to 562), also the creation of these subsets of lines was provided in detailed information in line number 139 to 149, based on the R reaction to *R. solani* (Fig. 2).

Query 2: In materials and methods, it was not clearly mentioned about the phenotyping of 330 rice accessions. Details about the statistical design used and no. of replicates used and traits recorded may be provided.

Response: We have provided brief experimental details and phenotypic assessment of these accessions in Lines 547 to 559. An initial statistical analysis approach was used to the "hclust and PCA" function in R program to identify a set of different rice accessions against to response of sheath blight. Details of downstream statistical analysis are provided in Line number 666 to 672.

Query 3. How it was concluded that all the agro morphological traits mentioned, like plant height to grain yield, were related to Sheath blight? (line no. 545 to 548)

Response: Thanks for your careful review and comments. According to the two seasons of phenotypic trait correlation studies, traits having association with PDI and AUPDC were identified as contributing traits (Line number 190 to 200). Hence, they were used for further studies.

Query 4. On what basis the accessions viz., IC 283139, IC22 277248, Sivappuchithirai Kar, and Bowalia were concluded as novel donors?

Response: With the critical assessment of the R reaction to *R. solani* from the two seasons experiment, these genotypes showed high resistance, based on the percentage disease index score, basal leaf sheath color and disease reaction as compared to known checks (Tapaswini and Pusa Basmati-1) (Detailed information were provided in Table 6), also these lines possess more than 6 different QTL combinations located on chromosome 1, 2, 4, 5, 9, 10, and 12. These novel sources of sheath blight resistance can be used in breeding programs to develop high resistance and grain yield improvement.

Reviewer #2:

It was very happy reading this manuscript. It is a good piece of work. Manuscript is nicely drafted and covered all the aspects. Methodology adopted and statistical analysis performed is appropriate.

Response: Thanks for your valuable suggestions and appreciate your feedback and positive comments to improve our manuscript. We have provided each of the comment's answers below.

• Title of manuscript: Dissecting genomic regions and underlying sheath blight resistance traits in rice (Oryza sativa L.) using a genome-wide association study

• Manuscript ID: 2023-01211-TW • It was very happy reading this manuscript. It is a good piece of work.

Manuscript is nicely drafted and covered all the aspects. Methodology adopted and statistical analysis performed is appropriate. Results obtained are satisfactory and useful. The manuscript contains useful information for the readers and researchers. My only concern is that the number of genotypes used (192) and number of SSR marker used (133) are very less for Genome Wide Association Study. If these parameters suit the journal criteria then the MS could be accepted for publication. Rice sheath blight is one of the most economically significant rice diseases worldwide. This disease causes significant grain yield and quality losses. Yield losses of up to 50% have been reported under most conducive environments. Sheath blight is a soil borne disease caused by the fungus Rhizoctonia solani AG1-IA. The fungus belongs to the phylum Basidiomycota, family Ceratobasidiaceae. Authors have evaluated a set of 330 traditional landraces and improved rice varieties for ShB resistance and created a core panel of 192 accessions for the GWAS. The core panel of rice accessions were evaluated for morphological and ShB disease resistance traits over the two seasons. Furthermore the panel was genotyped with 133 SSR microsatellite markers for association mapping for the ShB disease-resistance. Authors have performed several bio-statistical analyses from the agro-morphological traits/ disease score and SSR based molecular data. They have employed GLM and MLM (Q+K) models of GWAS and identified 30 marker trait associations for 18 targeted traits over seasons. Further, they have identified eight QTLs claimed them to be novel. The novel QTLs were located on 2, 4, 8, 10, and 12 chromosomes, which explained phenotypic variation ranging from 5% to 15%. Further they have identified six candidate genes, and three of them Os05t0566400, Os08t0155900, and Os09t0567300 were associated with defense mechanisms against ShB. In addition, they identified novel donors like IC 283139, IC 277248, Sivappuchithirai Kar, and Bowalia. The manuscript contains some useful information for the readers and researchers working for development of ShB resistant rice varieties. In the era of millions of SNPs, 133 SSR markers for used for genotyping of rice genotypes are very less especially for association mapping. Further, less number of SSRs would generate lower LD which restricted the high resolution mapping. The MTA or QTLs identified through GWAS are putative right now and it needs further validation for its confirmation in another population. However, statistical analysis performed from the agro-morphological data & molecular data by the authors are commendable. Furthermore, interpretation of the results with the help of tables, figures and graphs are also appreciable.

I have a few queries to the authors :

Query 1. Is a panel of 192 sufficient for GWAS?

Response: Thanks for the nice comments. In this study, we have followed a "step-by-step" approach to obtain enough population structure based on the admixture, correlated allele frequency, and molecular variance components within and between the selected populations. It provided a maximum variation (87%) found among the individuals. These panels are sufficient for GWAS to decrease false positive and high significant power to detect the trait-associations.

Several studies have been conducted even with 70 accessions. However, the population should have sufficient diversity, population structure, genetic variance, and allele frequency. Also, considering

global ancestry proportions or top principal components is enough to capture the significant variants. For example, in rice *(70 lines –Prakash et al. 2023 (Plant Physiology), Wheat (166 lines-Tong et al., 2022 (Front Plant Sci; doi: 10.3389/fpls.2022.840614), Rice (100 accessions-Ashfaq et al., 2023 (Genes) ; https://doi.org/10.3390/genes14051089*; 127 diverse rice genotypes - *Anandan et al., 2022 (Scientific Report); https://doi.org/10.1038/s41598-022-07781-5*; 119 diverse rice genotypes -*Pawar et al., 2021; PLOS ONE; https://doi.org/10.1371/journal.pone.0246232*); (John Witte, 2014-10.1146/annurev.publhealth.012809.103723; Alquadah et al., 2019 - https://doi.org/10.1016/j.jare.2019.10.013; Brachi et al., 2011; https://doi.org/10.1186/gb-2011-12-10-232

Query 2. Are a set of 133 SSRs sufficient for GWAS? Will they produce sufficient LD for high-resolution mapping?

Response: A total of 133 (103 for sheath blight-trait linked markers and 30 SSRs) SSRs were selected from the comprehensive literature survey and public domain database (http://gramene.org/) and used for genotyping of the population (Supplementary Table 2).-The markers were selected based on positions covering all the chromosomes to represent the maximum genome coverage and sufficient to capture the diversity and identify the polymorphic loci among the rice germplasms. On average, 11 markers per chromosome ranging from 6-15 were taken for the study, and the average distance of each marker was 3-4 Mb so that individual chromosomes could be covered.

The advantages of these trait-linked markers can sufficiently be captured within the LD block regions based on significant trait-SNP association, and recent evidence showed that trait-informative markers are strongly associated with GWAS studies (Chen et al., 2023). These markers are highly likely to detect the high SNP variants with significant associations. For GWAS, it is more important that evenly distributed markers across the chromosomes than the number of SNPs used.

Recent studies have used trait-linked markers for GWAS, considered to have high precision in identifying population structure and diversity (78 trait-linked markers: *Scientific Reports: Anandan et al., 2022*; 53 markers (47 SSR and 4 Gene-specific markers: *PLOS ONE: Pawar et al., 2021*; 58 markers (56 SSR and 2 trait-linked markers: *Front Plant Sci: Pandit et al, 2017*; 38 trait-linked markers; *PLOS ONE: Anandan et al., 2016*)

Query 3. Have you noticed any seasonal difference in the agro-morphological traits and disease reaction while screening the core panel in two different seasons i.e. Kharif and Rabi? Because both the seasons have completely different climatic conditions in terms of temperature, sunshine hours and humidity.

Response: Thanks for the insights. Yes, we have found small differences in disease reactions over the seasons. Few genotypes that showed a susceptible reaction in rabi (Fig S1) exhibited high susceptibility (HS) reaction in kharif (FigS2). We have already observed and reported weather parameters' role in disease establishment in our recent paper *(Naveen kumar et al. 2022 (Physiological and Molecular Plant Pathology)).* However, Sclerotia of R. solani remains in the soil even during hot sunny days. Additionally, we inoculated the sclerotia into the leaf sheaths of the rice plants where the water level was maintained in the field so that the pathogen received sufficient moisture for disease establishment.

Query 3. Is there any plan to validate the outcomes of the current study especially the novel MTA? How it could be done?

Response: Thanks for having positive expectations from us. We will take novel donors and MTA (marker-trait associations) into future breeding strategies to understand the genomic regions for dissecting the molecular and physiological pathways in pathogen defense mechanisms. Last year, we initiated a crossing block using novel donors into different breeding programs to introduce the identified QTLs. Additionally, transcriptome and QTL seq studies are in progress with the tolerant donors.

Query 4. What was basis for claiming the novelty of few MTAs? Have you used any database for it?

Response: The major criteria used to classify the few MTAs are novel based on the research evidence from the comprehensive literature survey on sheath blight resistance and Rice Research databases. The significant MTAs are located on different chromosomes 2, 4, 8, 10, and 12, which explains the phenotypic variation ranging from 5% to 15%, among them, novel MTAs (*qPh.2-1, qShB.4-1, qShB.4-2, qPh.8-1, qShB.8-2, qShB.10-1, qShB.10-2, and qPh.12-1*) are significantly associated with sheath blight traits, which is not reported so far.

We have used various databases to narrow down the candidate gene list, gene annotation, and gene network databases (RAP-DB, RGAP, Rice SNP-Seek Database, FRIEND database) to understand the known genomic regions that are associated with biotic disease and co-expression pattern of gene networks (Supplementary Figure 5. & Supplementary Table 10)
